# Highly pathogenic avian influenza A(H5N1) virus infections on fur farms connected to mass mortalities of black-headed gulls, Finland, July to October 2023

**DOI:** 10.2807/1560-7917.ES.2024.29.25.2400063

**Published:** 2024-06-20

**Authors:** Lauri Kareinen, Niina Tammiranta, Ari Kauppinen, Bianca Zecchin, Ambra Pastori, Isabella Monne, Calogero Terregino, Edoardo Giussani, Riikka Kaarto, Veera Karkamo, Tanja Lähteinen, Hanna Lounela, Tuija Kantala, Ilona Laamanen, Tiina Nokireki, Laura London, Otto Helve, Sohvi Kääriäinen, Niina Ikonen, Jari Jalava, Laura Kalin-Mänttäri, Anna Katz, Carita Savolainen-Kopra, Erika Lindh, Tarja Sironen, Essi M Korhonen, Kirsi Aaltonen, Monica Galiano, Alice Fusaro, Tuija Gadd

**Affiliations:** 1 Finnish Food Authority (FFA), Helsinki, Finland; 2 Istituto Zooprofilattico Sperimentale delle Venezie (IZSVe), Legnaro, Italy; 3 Finnish Food Authority, Seinäjoki, Finland; 4 Finnish Institute for Health and Welfare (THL), Department of Health Security, Helsinki, Finland; 5 University of Helsinki, Department of Veterinary Biosciences, Helsinki, Finland; 6 Worldwide Influenza Centre, Francis Crick Institute, London, United Kingdom

**Keywords:** Highly Pathogenic Avian Influenza A(H5N1), Fur animal, Finland, Mutation, black-headed gull, mink, fox, silver fox, raccoon dog

## Abstract

Highly pathogenic avian influenza (HPAI) has caused widespread mortality in both wild and domestic birds in Europe 2020–2023. In July 2023, HPAI A(H5N1) was detected on 27 fur farms in Finland. In total, infections in silver and blue foxes, American minks and raccoon dogs were confirmed by RT-PCR. The pathological findings in the animals include widespread inflammatory lesions in the lungs, brain and liver, indicating efficient systemic dissemination of the virus. Phylogenetic analysis of Finnish A(H5N1) strains from fur animals and wild birds has identified three clusters (Finland I-III), and molecular analyses revealed emergence of mutations known to facilitate viral adaptation to mammals in the PB2 and NA proteins. Findings of avian influenza in fur animals were spatially and temporally connected with mass mortalities in wild birds. The mechanisms of virus transmission within and between farms have not been conclusively identified, but several different routes relating to limited biosecurity on the farms are implicated. The outbreak was managed in close collaboration between animal and human health authorities to mitigate and monitor the impact for both animal and human health.

**Key public health message**

**What did you want to address in this study and why?**

From July to October 2023, Finland experienced an outbreak of highly pathogenic avian influenza (HPAI) H5N1 on fur farms. We analysed the outbreak to identify the source(s) of the infection and possible transmission routes. We outlined the measures taken to mitigate the impact on human health and to prevent future outbreaks.

**What have we learnt from this study?**

We detected the HPAI H5N1 virus on 27 fur farms and showed that the virus belonged to the genotype circulating in wild birds in the same area. Thus, the outbreak was likely caused by direct contact with infected wild birds followed by virus transmission within and between farms, as suggested by genomic and epidemiological data. This finding highlighted the need to improve biosecurity measures on fur farms.

**What are the implications of your findings for public health?**

This outbreak demonstrated the vulnerability of fur farms to pathogens that can have severe human health implications. The virus spread efficiently in the farmed animals, creating many opportunities for spillover to humans. Strict biosecurity measures to cut transmission routes and robust surveillance in animals and exposed people for early detection of infections are required for safe fur farming practices.

## Background

Highly pathogenic avian influenza (HPAI) H5 viruses of clade 2.3.4.4b have dominated among influenza virus infections in wild and domestic avian species in Europe since October 2020 [1], with several reported spillover events to wild mammals, mostly carnivores [2-4]. In autumn 2021, HPAI A(H5N1) caused deaths in reared pheasants and wild mammals in southern Finland [5]. Since then, outbreaks of HPAI subtype A(H5N1) among wild birds and wild mammals have been reported in multiple regions in Finland (updated online map of Finnish HPAI outbreaks available at the Finnish Food Authority (FFA), www.ruokavirasto.fi). In October 2022, HPAI A(H5N1) 2.3.4.4b infection was detected in Spain in a single fur farm housing 50,000 animals. The outbreak was contained by culling all animals on the affected facility [6].

Finland is a major producer of furs (1.5–2 million animals annually), and the species commercially farmed for fur include the arctic (blue) fox (*Vulpes lagopus*), the red (silver) fox (*Vulpes vulpes*), fox crossbreeds, the mink (*Neovison vison*), the raccoon dog (*Nyctereutes procyonoides*) and the sable (*Martes zibellina*) (https://fifur.fi/). Half of the animals reared are foxes, 41% are minks, 9% are raccoon dogs and < 0.1% sables. Most farms breed more than one species and according to the Central Database for Animal Keepers and Establishments maintained by the FFA, there are ca 450 fur animal holdings (accessed: 15 October 2023). Finland has a national plan for animal disease surveillance and in 2023, passive surveillance for HPAI in fur animals was added to the plan [7].

## Outbreak detection

Episodes of acute illness and mortality among farmed foxes, minks and raccoon dogs in Finland were reported to the FFA in July 2023 in the regions of South and Central Ostrobothnia. Following autopsy and real-time (RT)-PCR, the causative agent was identified as HPAI H5N1 with the first case confirmed on 13 July 2023 [8]. A case was defined as a farm where a positive H5N1 RT-PCR result from a swab, organ or faecal sample, even from a single animal, was obtained. Animals owned by different operators, but kept in the same establishment, were considered as a single epidemiological unit and managed as a single case.

This report describes the source of the viruses infecting the fur animals, the intra-farm virus evolution and virus adaptations to the mammalian hosts based on the full genome characterisation of A(H5N1) viruses collected from the infected fur farms and wild birds in the regions. Moreover, we provide information on the results of the epidemiological investigations, key pathological findings and control measures on fur farms.

## Methods

### Pathology

At the onset of the outbreak in early July, the pathology unit of the FFA Seinäjoki autopsied minks and foxes to investigate elevated mortality on the farms. Approximately 80% of the affected animals were young, born in 2023, and the animals were either found dead or had presented gastrointestinal or neurological symptoms. In the following weeks, 43 farms in the same region were sampled due to symptoms or contact with a positive farm, and follow-up samples were collected from 19 of the 27 HPAI-positive farms. We performed complete autopsies on 62 animals from 12 of the earliest affected farms, with organ sampling (lung, heart, liver, kidney, spleen, brain, trachea, urinary bladder, pancreas, lymph nodes (intestinal, subcapsular, mandibular, inguinal), small and large intestine) for histological, bacteriological and virological analyses and oropharyngeal swabs for virological analyses. For histology, organ samples from 33 animals from the 12 farms were fixed in 10% neutral buffered formalin, processed routinely, embedded in paraffin, cut into 4 µm sections and stained with haematoxylin and eosin (HE).

### Virus detection and whole-genome sequencing

For virus detection, we analysed serum, oropharyngeal or rectal swabs and/or tissue (lung, brain, kidney, intestine and spleen) samples. Not all sample types were taken from all 33 animals. We performed influenza A virus detection with RT-PCR and genes for haemagglutinin (H) and neuraminidase (N) with gel-based RT-PCRs, followed by Sanger sequencing for pathogenicity [5]. Given the uncertain distribution of the virus within the fur animal tissues, we performed RT-PCR for all available sample types. Sequencing and whole genome assembly details are presented in Supplementary File 1.

### Phylogenetic, network and evolutionary analyses

Consensus sequences of the eight gene segments were aligned with the most related sequences available in GISAID using MAFFT v.7 [9], as is shown in Supplementary Table S1. Maximum likelihood (ML) phylogenetic trees were obtained for each gene using IQTREE v1.6.6, performing ultrafast bootstrap resampling analysis (1,000 replications) and using the best-fitted nucleotide (nt) substitution model selected by ModelFinder [10-12]. Genotype assignment was based on the phylogenetic tree topology [13].

To assess the genetic relationship among the 115 highly related non-reassortant A(H5N1) viruses belonging to the phylogenetic group Finland-I, a phylogenetic network was generated using the Median Joining (MJ) method implemented in NETWORK 10.2.0.0 [14] for the eight segments. The MJ network uses a maximum parsimony approach to reconstruct the connection among viral genomes based on their genetic similarity, consisting of nodes and links connecting the nodes. The nodes can be either isolated viral strains or median vectors, representing hypothesised sequences used to connect the existing viral genomes in the most parsimonious way.

Evolutionary and discrete phylogeographic analyses of the concatenated genome of the HPAI A(H5N1) viruses, based on the date of sampling, were performed using BEAST v1.10.4 in combination with the BEAGLE library [15]. The dataset consists of 156 non-reassortant viruses of the EA-2022-BB (H5N1-A/Herring_gull/France/22P015977/2022-like) [13] genotype: 19 viruses from Europe, highly related to the viruses collected in Finland, 41 viruses collected from wild birds and 96 viruses collected from 24 fur farms in Finland. The dataset was analysed using the uncorrelated lognormal relaxed molecular clock, the SRD06 substitution model [16] and a coalescent constant population size model as tree prior [17-19].

To explore the spatial diffusion of the virus among wild birds and different farms, a discrete phylogeographic analysis was employed using 26 different traits: Europe, representing all the A(H5N1) viruses collected outside Finland, wild birds, containing all viruses collected from wild birds in Finland, and one trait for each fur farm (farm A to farm AA) [20]. We assumed an asymmetric non-reversible transition model and incorporated Bayesian stochastic search variable selection [20]. The MCMC chains were run for 100 million iterations, and convergence was assessed using Tracer v1.7.1 [16,21-23]. Spread D3 v0.9.6 [24] was used to assess Bayes factor (BF) supports and its Posterior Probability (PP) for each transition between discrete traits. We interpreted the strength of statistical support as strong for 20 < BF < 250 and very strong for BF > 250. The maximum clade credibility (MCC) tree was summarised using TreeAnnotator v1.10.4 and visualised in FigTree v1.4.4 (http://beast.bio.ed.ac.uk/TreeAnnotator/, http://tree.bio.ed.ac.uk/software/figtree/).

### Epidemiological investigation

Coordinated by the FFA, the fur farm operators were approached by the veterinary authorities in the Regional State Administrative Agencies (RSAA) for epidemiological investigation and interviewed using a questionnaire made for outbreak investigations on fur farms. The questionnaire covered mortality and clinical symptoms, patterns of disease, biosecurity measures and contacts with other animals or farms. The aim of the investigation was to identify the time and source of the infection and trace possible contact farms. Questions about personal protective equipment were also included and the names of the people in contact with sick animals were collected for public health authorities. In addition, information obtained about feed delivery routes, carcass collection and feed processing practices from feed operators were used in epidemiological investigation.

## Results

### Description of cases

By 1 October 2023, the FFA had analysed 1,451 animals from 43 farms using influenza A RT-PCR. The virus was detected from samples from 162 animals on 27 farms. Kaustinen and Kauhava were the most affected municipalities with 17 and 5 cases (farms with positive H5N1 RT-PCR result from at least one swab, organ or faecal sample), respectively. The HPAI-positive farms and their species distributions are shown in Table 1.

**Table 1:** Characteristics of farms with highly pathogenic avian influenza A(H5N1) virus infections in fur animals, Finland, July–October 2023 (n = 27)

Farm code	Municipality	Sampling date^a^	Number of animals			Initial sampling						Follow-up sampling (all species)		Clinical symptoms^c^
			Mink	Fox^b^	Raccoon dog	Mink		Fox^a^		Raccoon dog		Positive	Tested	
						Positive	Tested	Positive	Tested	Positive	Tested			
A	Kauhava	3 Jul	5,000–15,000	> 15,000	< 5,000	3	6	2	6	NT		25	96	Diarrhoea, neurological symptoms, mortality
B	Kauhava	6 Jul	0	> 15,000	0	NA		2	3	NA		5	117	Anorexia, neurological symptoms, mortality
D	Kaustinen	10 Jul	0	< 5,000	< 5,000	NA		4	4	NT		NT		Anorexia, apathy, mortality
E	Halsua	10 Jul	> 15,000	> 15,000	0	NT		3	5	NA		21	71	Anorexia, neurological symptoms, mortality
F	Kaustinen	14 Jul	0	< 5,000	0	NA		4	5	NA		1	30	Anorexia, apathy, neurological symptoms, mortality
G	Kaustinen	14 Jul	0	< 5,000	0	NA		4	4	NA		11	39	Diarrhoea, neurological symptoms, respiratory symptoms, mortality
H	Kaustinen	14 Jul	0	< 5,000	0	NA		3	5	NA		1	20	Anorexia, neurological symptoms, mortality
I	Evijärvi	17 Jul	0	< 5,000	< 5,000	NA		2	2	2	2	0	120	Anorexia, neurological symptoms, mortality
J	Kaustinen	17 Jul	0	< 5,000	0	NA		2	2	NA		0	35	Neurological symptoms, mortality
K	Kaustinen	17 Jul	0	< 5,000	0	NA		2	3	NA		1	35	Neurological symptoms, mortality
L	Kaustinen	18 Jul	0	< 5,000	0	NA		4	4	NA		0	20	Anorexia, apathy, neurological symptoms, mortality
M	Kauhava	18 Jul	0	5,000–15,000	0	NA		6	6	NA		0	130	Neurological symptoms, mortality
N	Kaustinen	20 Jul	0	< 5,000	0	NA		4	4	NA		NT		Apathy, mortality
O	Kaustinen	20 Jul	< 5,000	< 5,000	0	NT		3	3	NA		2	75	Anorexia, neurological symptoms (mink), respiratory symptoms, mortality
P	Kaustinen	10 Jul	0	< 5,000	0	NA		2	3	NA		NT		Anorexia, apathy, diarrhoea, mortality
Q	Kauhava	20 Jul	0	> 15,000	0	NA		3	4	NA		NT		Neurological symptoms
R	Kaustinen	24 Jul	0	< 5,000	0	NA		2	2	NA		NT		Anorexia, mortality
S	Kaustinen	24 Jul	0	< 5,000	0	NA		3	3	NA		1	90	Anorexia, apathy, neurological symptoms, mortality
T	Kaustinen	24 Jul	0	< 5,000	< 5,000	NA		4	4	3	3	NT		Mortality
U	Vöyri	25 Jul	0	> 15,000	0	NA		6	8	NA		0	120	Neurological symptoms, mortality
V	Kaustinen	27 Jul	0	< 5,000	0	NA		2	5	NA		NT		Mortality
W	Kauhava	31 Jul	5,000–15,000	5,000–15,000	0	0	3	1	5	NA		0	50	Anorexia, diarrhoea, neurological symptoms
X	Kannus	31 Jul	0	5,000–15,000	5,000–15,000	NA		7	8	NT		0	50	Anorexia, neurological symptoms, mortality
Y	Kaustinen	1 Aug	0	< 5,000	0	NA		7	7	NA		NT		Anorexia
Z	Kaustinen	21 Aug	0	< 5,000	0	NA		1	5	NA		0	30	No symptoms observed, normal mortality
AA	Alavieska	20 Sep	5,000–15,000	< 5,000	0	4	4	NA		NA		0	60	Anorexia, mortality

NA: not applicable; NT: not tested.

^a^ Date of sampling of the first positive sample.

^b^ All fox species (arctic fox, silver fox and crossbreds) included.

^c^ As reported by the farm owner.

### Pathological findings

**Figure 1:**
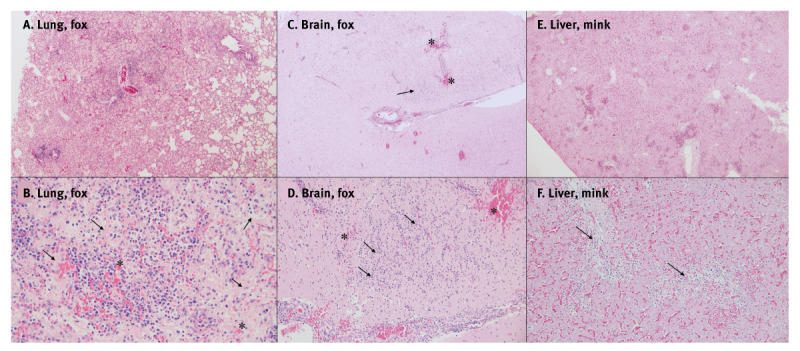
Histopathological findings of fur animals infected with highly pathogenic avian influenza A(H5N1) virus, Finland, July–October 2023 (n = 3)

Of the 33 animals whose organs were sampled, 27 exhibited a multifocal to diffuse necrosuppurative bronchointerstitial pneumonia and had multifocal areas with large amounts of activated alveolar macrophages, plump highly activated type II pneumocytes and alveolar hyaline membranes consistent with diffuse alveolar damage (Figure 1). Six animals had also diffuse acute fibrinoid pleuritis, and 14 of the 19 sampled brains had multifocal lesions in the cerebellar grey and white matter, occurring as mild to moderate necrotising meningoencephalitis, mononuclear perivascular cuffing and small haemorrhages. The livers sampled from 24 of 33 infected animals exhibited mild to severe multifocal to coalescing periportal to midzonal necrosis with occasional mineralisation.

Tissue section stained with haematoxylin and eosin under light microscopy.

A. Lung: large areas of consolidation with severe subacute focally extensive bronchointerstitial pneumonia and marked alveolar oedema (4X magnification).

B. Lung: necrotic area of the lung with infiltration of alveolar macrophages, lymphocytes and neutrophils, formation of hyaline membranes with intra-alveolar fibrin (arrows) and haemorrhage (asterisks) (40X magnification).

C. Brain, cerebral cortex: area of necrosis (arrow) and sulcus meninges with multifocal haemorrhages (asterisks) and increased cellularity (4X magnification).

D. Brain, cerebral cortex: focal cerebral necrosis showing severe subacute gliosis, multifocal haemorrhages (asterisks) and scattered necrotic cellular debris (arrows). Meninges in the sulcus display moderate mononuclear cell infiltration with multiple small haemorrhages and a few occasional neutrophils (20X magnification).

E. Liver: severe multifocal random haemorrhages and necrosis. (4X magnification).

F Liver: close-up showing severe periportal necrosis (arrows) and haemorrhage with infiltration of mononuclear cells and neutrophils (20X magnification).

Details of histopathological findings in different fur animals are presented in Table 2 and Supplementary File 1.

**Table 2:** Key pathological findings of histological examinations of fur animals infected with highly pathogenic avian influenza A(H5N1) virus, Finland, July–October 2023 (n = 33)

Animal		Histopathological finding				
Number	Species	Bronchointerstitial pneumonia	Meningoencephalitis	Liver necrosis	Peritonitis	Splenic lymphoid depletion
1	Mink	No	NA	Yes	No	Yes
2	Mink	Yes	NA	Yes	No	No
3	Mink	Yes	NA	Yes	No	Yes
4	Mink	Yes	NA	Yes	No	Yes
5	Mink	Yes	Yes	Yes	No	No
6	Silver fox	No	NA	Yes	No	Yes
7	Arctic fox	Yes	NA	Yes	Yes	No
8	Arctic fox	No	NA	No	Autolysis	Autolysis
9	Arctic fox	No	NA	No	Autolysis	Autolysis
10	Arctic fox	No	Yes	No	Yes	Yes
11	Arctic fox	Yes	Yes	No	Yes	No
12	Arctic fox	Yes	Yes	No	Yes	No
13	Arctic fox	Yes	No	Yes	No	No
14	Arctic fox	Yes	Yes	Yes	Yes	No
15	Arctic fox	Yes	Yes	Yes	No	Yes
16	Arctic fox	Yes	No	Yes	Yes	No
17	Arctic fox	Yes	NA	Yes	Yes	No
18	Arctic fox	Yes	Yes	Yes	No	Yes
19	Arctic fox	Yes	Yes	Yes	Yes	No
20	Arctic fox	Yes	Yes	Yes	Yes	No
21	Arctic fox	Yes	Yes	Yes	Yes	Yes
22	Arctic fox	Yes	Yes	Yes	Yes	Yes
23	Arctic fox	Yes	NA	Yes	Yes	Yes
24	Arctic fox	Yes	NA	Yes	Yes	No
25	Arctic fox	Yes	NA	No	Yes	No
26	Arctic fox	Yes	No	Yes	Yes	Yes
27	Arctic fox	Yes	No	Autolysis	Autolysis	Autolysis
28	Arctic fox	No	No	No	Yes	No
29	Arctic fox	Yes	Yes	Yes	No	No
30	Arctic fox	Yes	NA	Yes	No	Yes
31	Arctic fox	Yes	NA	Yes	Yes	No
32	Arctic fox	Yes	Yes	Yes	Yes	Yes
33	Arctic fox	Yes	Yes	No	Yes	Yes

NA: not applicable, no brain sample.

### Virological findings

**Figure 2:**
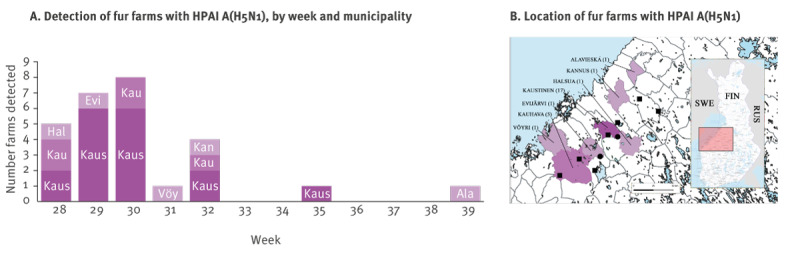
Timeline and location of fur farms with highly pathogenic avian influenza A(H5N1) virus infection, Finland, July–October 2023 (n = 27)

In addition to fur animals, we detected HPAI in seven wild birds (six black-headed gulls (*Chroicocephalus ridibundus*) and a common gull (*Larus ganus*)) and two wild mammals (an otter (*Lutra lutra*) and a fox (*Vulpes vulpes*)) from the same region. The timeline and a map of the confirmed HPAI cases in the affected area is presented in Figure 2.

Ala: Alavieska; Evi: Evijärvi; H: hemagglutinin; Hal: Halsua; HPAI: highly pathogenic avian influenza; Kan: Kannus; Kau: Kauhava; Kaus: Kaustinen; N: neuraminidase; Vöy: Vöyri.

Panel B: The number of cases per municipality is presented in parentheses. Rectangles represent findings of HPAI in wild birds and circles findings in wild mammals: an otter (*Lutra lutra*) in Halsua and a fox (*Vulpes vulpes*) in Evijärvi.

### Genetic analyses

The causative agent in the wild birds and the fur animals was confirmed as clade 2.3.4.4b. Phylogenetic analyses of the eight gene segments revealed that the viruses identified from all the fur animal samples and from 45 wild bird samples belonged to genotype EA-2022-BB, A/Herring_gull/France/22P015977/2022-like [13] and clustered together with A(H5N1) viruses of the same genotype that had circulated in Europe 2022–2023. The A(H5N1) viruses collected from wild birds of the EA-2022-BB genotype form three well-supported clusters (Finland-I, Finland-II and Finland-III), presented in Supplementary File 1.

**Figure 3:**
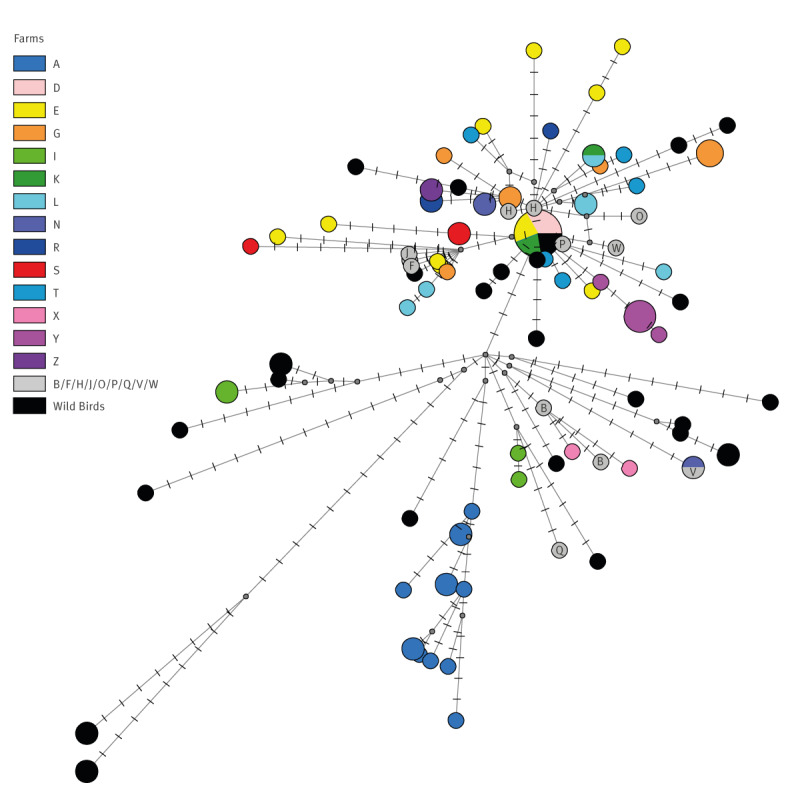
Phylogenetic network for the eight concatenated gene segments of non-reassortant highly pathogenic avian influenza A(H5N1) viruses from fur animals and wild birds belonging to genotype EA-2022-BB, cluster Finland-I, Finland, July–October 2023 (n = 141)^a^

^a^ The network is generated using the Median Joining method implemented in NETWORK 10.2.0.

Except for the A(H5N1) from farm AA, belonging to cluster Finland-II, all viruses from the fur farms clustered (similarity of 99.6–100%) within Finland-I. Network analyses performed on the complete genome sequences (concatenated genes) of viruses of cluster Finland-I (Figure 3) shows that viruses collected from farms D, E, F, G, H, J, K, L, M, N, O, P, R, S, T, W and Y were closely related to each other. Analysis of the intra-farm genetic diversity showed that viruses collected from farms E, G, I, L, S and T did not show a genetic clustering by farm. In contrast, samples collected from farms A, Y and AA formed well-defined clusters, strongly suggesting a single virus introduction followed by a within-farm virus evolution (Figure 3), Supplementary File 1. Sequencing of the fur animal samples revealed several mammalian adaptations, and the full list of the detected mutations is presented in Supplementary Table S1.

Circles: viral types; the size is proportional to the number of viruses sharing the same type; the length of the branches is proportional to the number of nucleotide differences.

### Evolutionary analysis

**Figure 4:**
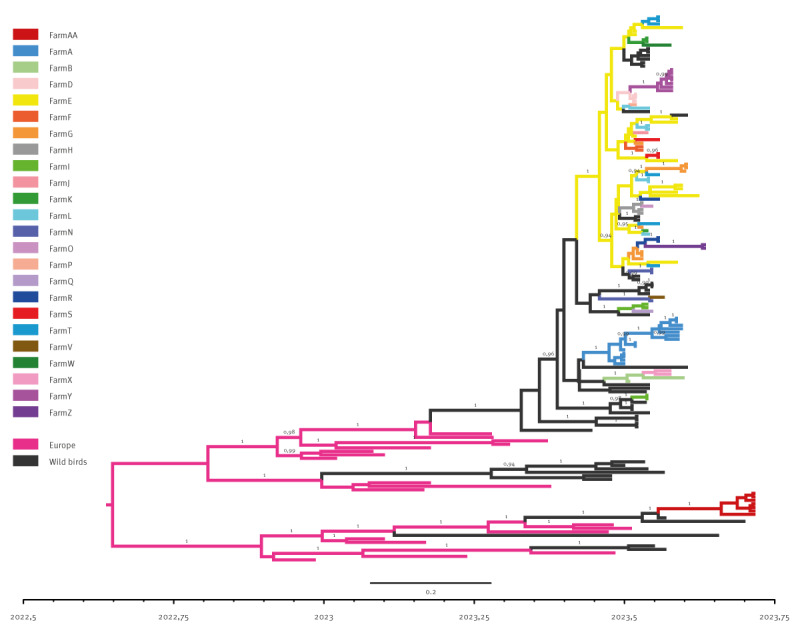
Discrete phylogeographic analysis based on the complete genome of highly pathogenic avian influenza A(H5N1) viruses, belonging to genotype EA-2022-BB from fur animals and wild birds, Finland, July–October 2023 (n = 141)

The discrete phylogeographic analysis (Figure 4) indicates at least five separate viral incursions to Finland from other European countries (BF = 72879,5, PP = 0.99), likely during spring or summer 2023.

Posterior probability values higher than 0.9 are indicated above the branches.

Based on this analysis, wild birds acted as an important virus source for the affected fur farms. Similarly to the sequence analysis, the phylogeographic analysis also suggests a single virus incursion into farms A, Y and AA followed by intra-farm transmission and evolution. Data also indicate virus transitions from farm E to farms G, L, T, S, H, F, R, K and D, as well as back to the wild birds (Figure 4) and Supplementary File 1.

### Epidemiological findings

Increased mortality was reported by 23 farmers, and on two fox farms this was the only symptom reported (Table 1). There was considerable variation in the way the mortalities were recorded by the farmers, but excess daily mortality was estimated by the farmers to range from none to nearly 10 times the normal seasonal average. The pattern of disease within the farms varied. Twelve farms reported only scattered cases, while six had observed sequential cases confined to a few shadow houses, whereas nine reported a progression of cases across multiple houses.

Fur animals were kept in shadow houses, in which two rows of metal wire cages, elevated 30–50 cm from the ground, are kept under a roof with an access corridor between the two rows, doorways at both ends and no solid walls. The affected farms were situated in an area with several lakes hosting populations of black-headed gulls. Wild bird mortalities in these areas were considered caused by HPAI. The gulls fly widely in the area and regularly feed on fur farms. While 16 of the 27 farms had structural or active deterrents against birds, these were typically only partially implemented, e.g. not used in all shadow houses and 11 reported having no measures in use against birds. Thus, the birds had easy access to animal feed inside the shadow houses and under the wire cages; as a consequence, direct contact with the fur animals represented the most likely transmission route to the farms.

When investigating connections between farms, some possibilities for between-farm transmission were identified. Dead and culled animals, which might have already been infected, were taken from the farms to be processed as feed for other fur animals. There were no biosecurity measures at the carcass collection point at about 5–35 km distance from most farms in Kaustinen and used by most of the farmers. This could have acted as a point of virus spread between farms via vehicles, clothing or wild birds feeding on the carcasses. Even though the processing method of the carcasses is adequate to inactivate the virus, the processed feed may have been contaminated by bird faeces after processing and before distribution. The feed trucks, which deliver feed to many farms along the same route, fill the feed silos either from outside or inside the farm premises, however, no biosecurity measures were taken at the farm entrances. Despite this, the pattern of disease incidents and timing of infections excludes a major role for feed delivery or contamination of feed at the processing plant or interactions at the carcass collection site. Human-driven transmission event is likely to have occurred between farms B and X, which are located 75 km apart. The farms have genetically closely clustering viruses and the same owner, who handled animals on both farms, did not change clothing between farms.

## Outbreak control measures

Immediately after the confirmed diagnosis of HPAI on 13 July 2023, the FFA notified the Ministry of Agriculture and Forestry (MAF) and the regional veterinary authorities in the RSAA. Due to the zoonotic aspect of the finding, the Finnish Institute of Health and Welfare was also informed. The first meeting with these authorities took place on 14 July 2023. A national disease control group was established, which included the above-mentioned authorities, the Ministry of Social Affairs and Health and the Finnish Institute of Occupational Health. The group acted as a forum for information sharing and insuring a common platform for discussion, policy formulation and communication activities. While the RSAA acted as a regional disease control centre, the FFA acted as a national control centre and co-operated closely with the European Union Reference Laboratory for Avian Influenza (Istituto Zooprofilattico Sperimentale delle Venezie, Italy) in analysing the outbreak data.

To provide a legal framework for veterinary authorities to control HPAI on fur farms and protect humans from infection, on 18 July 2023, the MAF reclassified HPAI in fur animals from notifiable diseases to other diseases to be combated (MAF 909/2023 https://www.finlex.fi/fi/laki/alkup/2023/20230909). This allowed the RSAA to impose restrictions on farms, such as forbidding animal movements to and from the affected farms, carry out epidemiological surveys and trace contact farms for sampling. In addition, the FFA had the legal authority to cull animals on infected farms and the owners were entitled to governmental compensations for the financial losses. The MAF issued a more detailed national regulation (MAF 1068/2023 https://www.finlex.fi/fi/laki/alkup/2023/20231068) on combating H5 avian influenza in fur animals on 8 December 2023. This regulation detailed a more specific framework for culling decisions and preconditions for restocking the affected farms. Weekly meetings were organised with the Finnish Fur Animal Association to ensure flow of communication and stakeholder support. The objective was to detect the infected farms as quickly as possible and prevent further transmission of the virus to fur farms, other animal farms or humans.

We initially conducted culling only in shadow houses with minks or other symptomatic fur animals. However, with more information available from virus sequencing and potential inter-species transmissions, the depopulation was extended to cover all fur animals on the infected farms. Culling was organised by the RSAA, and all the carcasses were taken to a rendering plant to be processed. Altogether ca 250,000 fur animals from the 27 infected farms were culled and processed.

## Discussion

Deficiencies in the biosecurity measures on fur farms allowing direct bird-mammal contact are a known risk factor for transmission of pathogens from wildlife to reared mammals [25]. Based on the findings from the genetic analyses, concurrent HPAI-related wild bird mass mortalities in areas surrounding the fur farms and the epidemiological investigations, the outbreak seems to be a direct consequence of the large-scale exposure of fur animals to infected wild birds.

During the outbreak, the A(H5N1) virus caused variable clinical symptoms in the animals, ranging from asymptomatic infections to fatal pneumonia and meningitis. The virus appeared neurotropic in fur animals, as the most common symptoms included neurological symptoms, such as tremors, disorientation and apathy, while respiratory symptoms were less frequent. The severe inflammatory lesions in the lungs and the brain are indicative of the severe effect the virus can have on the host and underline the serious risks associated with any possible human cases.

Features of the outbreak have also diagnostic implications. During the outbreak, we considered using oropharyngeal swabs as a scalable, easy-to-implement sampling method to rapidly assess the situation on the farms in the affected area. However, the virus was not consistently detected from swab samples and in some animals only the brain or lung tissue tested positive. Because of this, we recommend that any investigation of suspected HPAI in a fur animal should always include testing of tissue samples.

In our molecular analyses of the viruses collected from fur animals, we detected mutations in the PB2 and NA genes associated with adaptation to mammalian cells, with many viruses having multiple adaptations. The PB2-T271A increases the polymerase activity in mammalian cell lines and was detected in arctic foxes from three farms (B, X and E) [26]. The PB2-E627K, found in artic foxes and minks (farms A, I, S and AA), has been demonstrated to enhance pathogenicity and mortality in mammals, polymerase activity and virus replication [27-30]. Two samples from farms I and S had mixed 627E/K populations, which may indicate that the mutation emerged during the virus replication in the sampled animal or that two virus variants were co-circulating on the farm. A combination of NA-S369I and NA-I396V mutations, causing a disruption of the second sialic acid binding site (2SBS), was detected on farm A that also had PB2-E627K. Loss of the 2SBS may drive selection for changes in the receptor binding properties of HA, possibly resulting in increased binding to human-type receptors [31,32] This combination of mutations had not been previously identified in the 2022–2023 European A(H5N1) viruses. The NP-Y52N, present in all viruses analysed from fur farms, is associated with evasion of human BTN3A3, a potent inhibitor of avian but not human influenza A virus replication. Moreover, PB2-Q236K, detected on three farms (G, L and T), has been demonstrated to improve virus replication by ca 10–50-fold in differentiated human tracheo-bronchial epithelial cells [33]. Finally, PB2-A588T, found on one farm (I), was shown to increase the polymerase activity in human embryonic kidney 293T cells [34]. A thorough analysis using cell line experiments is warranted to fully understand the impact of these mutations to the pathogenicity of the virus variants.

Direct mammal-to-mammal or an indirect fomite driven transmission is implicated, and the genetic clustering of viruses on some of the analysed farms indicates that this may have happened. Within-farm virus evolution was particularly evident on farm A, from which sequences of viruses collected at two time points (3 July and 7 August) were available. All sequences from the farm cluster consistent with a single introduction followed by direct or indirect transmission. These viral sequences possess mutations not previously detected in birds and likely acquired after the introduction of the virus into the farms. Combined with the genetic data, our findings indicate that virus transmission between fur animals has most likely occurred, and this should be considered when planning strategies to prevent and combat HPAI in fur animals.

Phylogeographic analyses seem to indicate farm E as an important local hub in the outbreak, which contributed to the spread of the virus to other farms and back to wild birds. However, sampling bias cannot be excluded given (i) the higher number of available sequences of viruses collected from this farm (n = 12) compared with most of the other farms (1 < n < 14, mean 3.6 per farm) and (ii) the limited number of sequencing data from wild birds (n = 40). Although no direct connection from farm E to the other affected farms was identified by the epidemiological investigation, other routes of virus transmission, such as wild birds and human-related activities at the carcass collection point, cannot be ruled out. Farm E was among the largest facilities affected in this outbreak, with multiple incursions of highly related viruses from wild birds. Unfortunately, we have very few virus sequences from wild bird mass mortalities from the surrounding area, and therefore, the findings from farm E may reflect the virus diversity in the bird population rather than extensive intra-farm evolution and between-farm transmission.

Based on the available data, we cannot conclude how and to what extent the virus has been transmitted between farms. Results from the epidemiological investigation showed that many farm practices may enable the spread of the virus, such as the open shadow houses, infrequent use of gloves or hand sanitisers and not changing protective clothing. Poor biosecurity measures at the carcass collection point, contaminated feed or vehicles may all have played a role, as well as the possibility of the birds or small rodents acting as vectors. Several of the farms are close to one another, and some share equipment, favouring possible transmission across farms when biosecurity and good hygiene practices are not followed. Further data are required to better understand how the outbreak spread and why so many farms in the area were affected.

In controlling the outbreak, a close co-operation between national and international veterinary and public health authorities was implemented. Containing the outbreak, limiting any exposure risks to humans and preventing future epidemics are the key priorities.

## Conclusion

This large HPAI A(H5N1) outbreak demonstrates the vulnerability of densely farmed mammalian populations to pathogens present in the surrounding environment. To mitigate the continuing risk of novel outbreaks of avian influenza, efficient biosecurity measures should be implemented to minimise direct and indirect contact between farmed mammals and environmental sources, especially wild birds. In Finland, a national regulation on biosecurity requirements on all fur farms to prevent birds having contact with the animals was issued by MAF on 15 January 2024 (MAF 14/2024 https://www.finlex.fi/fi/laki/alkup/2024/20240014?search%5Btype%5D=pika&search%5Bpika%5D=14%2F2024). Although prevention of outbreaks is the main target, improving preparedness and response capacity of both fur farms and authorities is essential, as well as being alert for any increase in mortality on the farms. Virological surveillance designed for early detection of outbreaks is an essential part of disease control and we recommend active monitoring, especially when HPAI is found in bird populations in the vicinity of animal farms. The importance of implementing safer fur farming practices is highlighted by the observations of genetic changes during the outbreak associated with mammalian adaptation, which may increase the pandemic potential of the circulating avian influenza viruses.

## Supplementary Data

2166000 Supplementary Material 1

## Supplementary Data

84000 Supplementary Material 2
