# Supplementary material for: Highly pathogenic avian influenza A(H5N1) virus infections on fur farms connected to mass mortalities of black-headed gulls, Finland, July to October 2023
Source: Euro Surveill. 2024 Jun 20;29(25):2400063. doi: 10.2807/1560-7917.ES.2024.29.25.2400063 (PMC11191417; doi:10.2807/1560-7917.ES.2024.29.25.2400063)
Supplement: Supplementary Material 1 [file 24-00063_KAREINEN_SupplementaryFile1.pdf]

This supplementary material is hosted by Eurosurveillance as supporting information alongside the article “Highly Pathogenic Avian Influenza A(H5N1) Virus Infections in Fur Farms Connected to Mass Mortalities of Black-Headed Gulls in Finland, July-October 2023”, on behalf of the authors, who remain responsible for the accuracy and appropriateness of the content. The same standards for ethics, copyright, attributions and permissions as for the article apply. Supplements are not edited by Eurosurveillance and the journal is not responsible for the maintenance of any links or email addresses provided therein.

SEQUENCING AND GENOME ASSEMBLY

Complete influenza A virus genomes were sequenced with the Illumina MiSeq platform by FFA and Istituto Zooprofilattico Sperimentale delle Venezie (IZSVe) and MinION Sequencing device (Oxford Nanopore) platform by Finnish Institute for Health and Welfare (THL). PCR amplicons were produced using the Uni12/Inf1, Uni12/Inf3, Uni13/Inf1 primers [1,2]. For Illumina Miseq platform, sequencing libraries were obtained using the Nextera XT DNA Library Preparation Kit (Illumina) and quantified using Qubit (Invitrogen, USA). The average fragment length was determined using the Agilent High Sensitivity Bioanalyzer Kit. The indexed libraries were pooled in equimolar concentrations and sequenced using the MiSeq Reagent Micro Kit v2 (300-cycles, Illumina). For Oxford Nanopore sequencing, Rapid Barcoding Kit 96 (SQK-RBK110-96) was used with either 5 ul or 7,5 ul of sample. About 700 ng of library was loaded into a MinION Sequencing device and run for up to 24 hours. In addition, the sequencing at the IZSVe was performed on an Illumina MiSeq platform as previously described [3].

The whole genome assembly was performed at FFA, IZSVe and the Worldwide Influenza Centre (WIC), Francis Crick Institute (London, UK). At FFA, the sequence data was analysed using CLC Genomics Workbench (Qiagen). Raw reads were quality trimmed and adapter sequences were removed and trimmed reads were mapped against a reference sequence. The consensus sequences were generated with a minimum coverage depth of 6. At IZSVe, data was analysed using an in-house pipeline [1]. After cleaning and trimming, high quality reads were aligned against a reference genome using BWA v0.7.1229. Alignments were processed with Picard tools v2.1.0 (http://picard.sourceforge.net) and GATK v3.530–32 to correct potential errors, realign reads around indels and recalibrate base quality. Single nucleotide polymorphisms (SNPs) were called using LoFreq v2.1.233, and the outputs were used to generate consensus sequences. At the WIC, genome assembly was performed with the CDC IRMA pipeline, with a minimum depth of 6 [4].

1. Zhou B, Donnelly ME, Scholes DT, St George K, Hatta M, Kawaoka Y, et al. Single-reaction genomic amplification accelerates sequencing and vaccine production for classical and Swine origin human influenza A viruses. J Virol. 2009 Oct;83(19):10309-13. doi: 10.1128/JVI.01109-09. Epub 2009 Jul 15. PMID: 19605485; PMCID: PMC2748056.
2. Wüthrich D, Lang D, Müller NF, Neher RA, Stadler T, Egli A. Evaluation of two workflows for whole genome sequencing-based typing of influenza A viruses. J Virol Methods. 2019 Apr;266:30-33. doi: 10.1016/j.jviromet.2019.01.009. Epub 2019 Jan 21. PMID: 30677464.

3. Tammiranta N, Isomursu M, Fusaro A, Nylund M, Nokireki T, Giussani E, et al. Highly pathogenic avian influenza A (H5N1) virus infections in wild carnivores connected to mass mortalities of pheasants in Finland. Infect Genet Evol. 2023 Jul:111:105423. doi: 10.1016/j.meegid.2023.105423. Epub 2023 Mar 6.
4. Shepard, S.S., Meno, S., Bahl, J. et al. Viral deep sequencing needs an adaptive approach: IRMA, the iterative refinement meta-assembler. BMC Genomics 17, 708 (2016). doi: 10.1186/s12864-016-3030-6

GROSS PATHOLOGY

Most of the infected animals had multifocal to coalescing, variably sized haemorrhagic areas extending from the pleural surface to deep lung parenchyma. Some had petechial haemorrhages on the surface of the lungs, bloody fluid or extensive mucus in the trachea and mild to moderate amounts of clear to bloody pleural or peritoneal effusion. Spleens were mildly to markedly enlarged and dark. In most animals the liver was either diffusely pale or mottled or there were some petechial haemorrhages on the capsule. Some animals had mildly to markedly congested meninges. Many animals had empty stomachs and some mucoid or loose contents in the intestines.

HISTOPATHOLOGY

LUNG

Majority (27/33) of both foxes and minks exhibited a multifocal to diffuse necrosuppurative bronchointerstitial pneumonia. 6 animals had also diffuse acute fibrinoid pleuritis. 26/33 of the animals had multifocal areas with large amounts of activated alveolar macrophages, plump highly activated type II pneumocytes and alveolar hyaline membranes consistent with diffuse alveolar damage. 7 animals had moderate amounts of secondary bacterial colonies in the alveoli. A few (8/33) animals additionally had multifocal small randomly scattered interstitial mineralizations and 12 animals had mild multifocal erosions of tracheal epithelium.

BRAIN

Out of 19 animals that had brain histologically sampled, 14 had brain lesions multifocally in the cerebellar grey and white matter. They exhibited a mild to moderate multifocal subacute necrotizing meningoencephalitis with multifocal mononuclear perivascular cuffing and scattered small hemorrhages.

LIVER

The livers of 24/33 infected animals had mild to severe multifocal to coalescing periportal to midzonal or occasional random necrosis with occasional variable sized multifocal mineralizations.

SPLEEN

In spleens, 11 foxes and 3 minks had mild to severe depletion of lymphoid tissue with replacement of myriad of macrophages and multifocal hemorrhages. Most foxes and minks had moderate to severe congestion in the spleen and the splenic tissue of minks was spared with moderate to marked increase in megakaryocytes.

Out of 7 animals that had lymph nodes histologically sampled, 5 had lymph nodes mildly to moderately considered as reactive with activated secondary lymphoid follicles, mildly increased amounts of plasma cells and a medullary influx of macrophages.

INTESTINE

19 foxes had a diffuse, acute fibrinoid peritonitis. 14/33 of all animals had mild multifocal cryptal epithelial cell necrosis in small or large intestine.

BACTERIOLOGY AND ADDITIONAL VIROLOGY

Most of the autopsied animals had variable bacterial growth in different organs. Detected bacteria (Streptococcus sp., Staphylococcus sp., Escherichia coli, Pasteurella sp. and Campylobacter sp.) are commonly found in fur animal samples in Finland.

Samples for canine distemper virus were taken as pooled samples per farm from the necropsied animals. Samples for parvovirus were taken from those animals that had diarrhea. Samples for canine adenovirus were taken from two foxes and SARS-CoV-2 samples were taken from the necropsied minks. All the virus assays for canine distemper virus, parvovirus, canine adenovirus and SARS-CoV-2 were negative.

EVOLUTIONARY ANALYSIS

Supplemental Table 1. The Bayesian factors and posterior probabilities of virus transmission events between the wild birds and farms that were identified in the evolutionary analysis.

From	To	Bayes factor	Posterior probability
Europe	Wild birds	72879,5	1,00
Wild birds	Farm I	4349,9	0,99
Wild birds	Farm E	67,3	0,73
Wild birds	Farm A	59,7	0,71
Wild birds	Farm B	41,1	0,63
Wild birds	Farm AA	25,7	0,51
Farm B	Farm X	158,1	0,87
Farm E	Farm G	6408,4	1,00
Farm E	Wild birds	2207,5	0,99
Farm E	Farm L	993,00	0,98
Farm E	Farm T	340,2	0,93
Farm E	Farm S	214,7	0,90
Farm E	Farm H	76,8	0,76
Farm E	Farm F	54,5	0,69
Farm E	Farm R	51,5	0,68
Farm E	Farm K	49,0	0,67
Farm E	Farm D	29,1	0,55
Farm H	Farm O	2461,1	0,99
Farm I	Farm Q	61,1	0,72

Farm N	Farm V	90,2	0,79

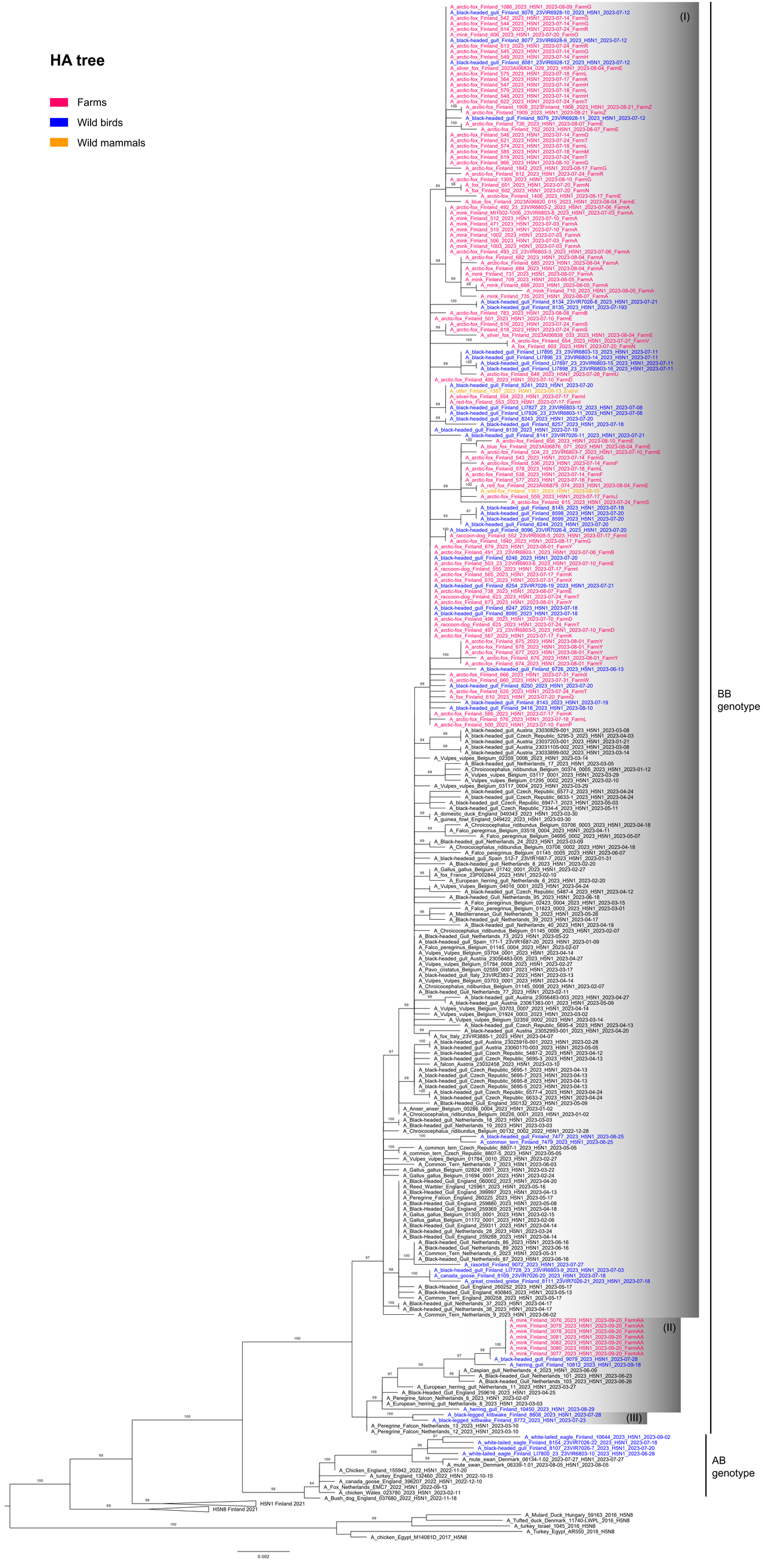
HA tree
Farms
Wild birds
Wild mammals
(I)
(II)
(III)
BB genotype
AB genotype
A_arctic-fox_Finland_1086_2023_H5N1_2023-08-09_FarmG
A_black-headed_gull_Finland_8078_23VIR6928-10_2023_H5N1_2023-07-12
A_arctic-fox_Finland_542_2023_H5N1_2023-07-14_FarmG
A_arctic-fox_Finland_544_2023_H5N1_2023-07-14_FarmG
A_arctic-fox_Finland_614_2023_H5N1_2023-07-24_FarmR
A_mink_Finland_606_2023_H5N1_2023-07-20_FarmO
A_black-headed_gull_Finland_8077_23VIR6928-9_2023_H5N1_2023-07-12
A_arctic-fox_Finland_613_2023_H5N1_2023-07-24_FarmR
A_arctic-fox_Finland_545_2023_H5N1_2023-07-14_FarmG
A_arctic-fox_Finland_549_2023_H5N1_2023-07-14_FarmH
A_black-headed_gull_Finland_8081_23VIR6928-12_2023_H5N1_2023-07-12
A_silver_fox_Finland_2023AI06834_029_2023_H5N1_2023-08-04_FarmE
A_arctic-fox_Finland_575_2023_H5N1_2023-07-18_FarmL
A_arctic-fox_Finland_564_2023_H5N1_2023-07-17_FarmK
A_arctic-fox_Finland_547_2023_H5N1_2023-07-14_FarmH
A_arctic-fox_Finland_579_2023_H5N1_2023-07-18_FarmL
A_arctic-fox_Finland_548_2023_H5N1_2023-07-14_FarmH
A_arctic-fox_Finland_622_2023_H5N1_2023-07-24_FarmT
A_arctic-fox_Finland_1908_2023Finland_1908_2023_H5N1_2023-08-21_FarmZ
A_arctic-fox_Finland_1909_2023_H5N1_2023-08-21_FarmZ
A_black-headed_gull_Finland_8079_23VIR6928-11_2023_H5N1_2023-07-12
A_arctic-fox_Finland_736_2023_H5N1_2023-08-07_FarmE
A_arctic-fox_Finland_752_2023_H5N1_2023-08-07_FarmE
A_arctic-fox_Finland_546_2023_H5N1_2023-07-14_FarmG
A_arctic-fox_Finland_621_2023_H5N1_2023-07-24_FarmT
A_arctic-fox_Finland_574_2023_H5N1_2023-07-18_FarmL
A_arctic-fox_Finland_585_2023_H5N1_2023-07-18_FarmM
A_arctic-fox_Finland_619_2023_H5N1_2023-07-24_FarmT
A_arctic-fox_Finland_966_2023_H5N1_2023-08-10_FarmG
A_arctic-fox_Finland_1842_2023_H5N1_2023-08-17_FarmG
A_arctic-fox_Finland_612_2023_H5N1_2023-07-24_FarmR
A_arctic-fox_Finland_1305_2023_H5N1_2023-08-10_FarmG
A_fox_Finland_601_2023_H5N1_2023-07-20_FarmN
A_fox_Finland_602_2023_H5N1_2023-07-20_FarmN
A_arctic-fox_Finland_1406_2023_H5N1_2023-08-17_FarmE
A_blue_fox_Finland_2023AI06820_015_2023_H5N1_2023-08-04_FarmE
A_arctic-fox_Finland_492_23_23VIR6803-2_2023_H5N1_2023-07-06_FarmA
A_mink_Finland_Mink_1006_23VIR6803-4_2023_H5N1_2023-07-03_FarmA
A_mink_Finland_512_2023_H5N1_2023-07-10_FarmA
A_mink_Finland_471_2023_H5N1_2023-07-03_FarmA
A_mink_Finland_515_2023_H5N1_2023-07-10_FarmA
A_mink_Finland_1002_2023_H5N1_2023-07-03_FarmA
A_mink_Finland_506_2023_H5N1_2023-07-03_FarmA
A_mink_Finland_1003_2023_H5N1_2023-07-03_FarmA
A_arctic-fox_Finland_493_23_23VIR6803-3_2023_H5N1_2023-07-06_FarmA
A_arctic-fox_Finland_682_2023_H5N1_2023-08-04_FarmA
A_arctic-fox_Finland_685_2023_H5N1_2023-08-04_FarmA
A_arctic-fox_Finland_684_2023_H5N1_2023-08-04_FarmA
A_mink_Finland_731_2023_H5N1_2023-08-07_FarmA
A_mink_Finland_709_2023_H5N1_2023-08-05_FarmA
A_mink_Finland_688_2023_H5N1_2023-08-05_FarmA
A_mink_Finland_710_2023_H5N1_2023-08-05_FarmA
A_mink_Finland_735_2023_H5N1_2023-08-07_FarmA
A_black-headed_gull_Finland_8134_23VIR7026-8_2023_H5N1_2023-07-21
A_black-headed_gull_Finland_8135_2023_H5N1_2023-07-193
A_arctic-fox_Finland_783_2023_H5N1_2023-08-08_FarmS
A_arctic-fox_Finland_501_2023_H5N1_2023-07-10_FarmE
A_arctic-fox_Finland_616_2023_H5N1_2023-07-24_FarmS
A_arctic-fox_Finland_618_2023_H5N1_2023-07-24_FarmS
A_silver_fox_Finland_2023AI06838_033_2023_H5N1_2023-08-04_FarmE
A_arctic-fox_Finland_664_2023_H5N1_2023-07-27_FarmV
A_fox_Finland_603_2023_H5N1_2023-07-20_FarmN
A_black-headed_gull_Finland_LI7895_23_23VIR6803-13_2023_H5N1_2023-07-11
A_black-headed_gull_Finland_LI7896_23_23VIR6803-14_2023_H5N1_2023-07-11
A_black-headed_gull_Finland_LI7897_23_23VIR6803-15_2023_H5N1_2023-07-11
A_black-headed_gull_Finland_LI7898_23_23VIR6803-16_2023_H5N1_2023-07-11
A_arctic-fox_Finland_548_2023_H5N1_2023-07-28_FarmU
A_arctic-fox_Finland_495_2023_H5N1_2023-07-10_FarmD
A_black-headed_gull_Finland_8241_2023_H5N1_2023-07-20
A_otter_Finland_1387_2023_H5N1_2023-08-13_EstrH
A_silver-fox_Finland_554_2023_H5N1_2023-07-17_FarmI
A_red-fox_Finland_553_2023_H5N1_2023-07-17_FarmI
A_black-headed_gull_Finland_LI7827_23_23VIR6803-12_2023_H5N1_2023-07-08
A_black-headed_gull_Finland_LI7826_23_23VIR6803-11_2023_H5N1_2023-07-08
A_black-headed_gull_Finland_8243_2023_H5N1_2023-07-20
A_black-headed_gull_Finland_8257_2023_H5N1_2023-07-18
A_black-headed_gull_Finland_8139_2023_H5N1_2023-07-19
A_black-headed_gull_Finland_8141_23VIR7026-11_2023_H5N1_2023-07-21
A_arctic-fox_Finland_856_2023_H5N1_2023-08-10_FarmE
A_blue_fox_Finland_2023AI06876_071_2023_H5N1_2023-08-04_FarmE
A_arctic-fox_Finland_504_23_23VIR6803-7_2023_H5N1_2023-07-10_FarmE
A_arctic-fox_Finland_543_2023_H5N1_2023-07-14_FarmG
A_arctic-fox_Finland_536_2023_H5N1_2023-07-14_FarmF
A_arctic-fox_Finland_578_2023_H5N1_2023-07-18_FarmL
A_arctic-fox_Finland_538_2023_H5N1_2023-07-14_FarmF
A_arctic-fox_Finland_577_2023_H5N1_2023-07-18_FarmL
A_red_fox_Finland_2023AI06879_074_2023_H5N1_2023-08-04_FarmE
A_wild-fox_Finland_1391_2023_H5N1_2023-08-10
A_arctic-fox_Finland_559_2023_H5N1_2023-07-17_FarmJ
A_arctic-fox_Finland_615_2023_H5N1_2023-07-24_FarmS
A_black-headed_gull_Finland_8145_2023_H5N1_2023-07-19
A_black-headed_gull_Finland_8598_2023_H5N1_2023-07-20
A_black-headed_gull_Finland_8599_2023_H5N1_2023-07-20
A_black-headed_gull_Finland_8244_2023_H5N1_2023-07-20
A_black-headed_gull_Finland_8096_23VIR7026-6_2023_H5N1_2023-07-20
A_raccoon-dog_Finland_552_23VIR6928-5_2023_H5N1_2023-07-17_FarmI
A_arctic-fox_Finland_1840_2023_H5N1_2023-08-17_FarmG
A_arctic-fox_Finland_679_2023_H5N1_2023-08-01_FarmY
A_arctic-fox_Finland_491_23_23VIR6803-1_2023_H5N1_2023-07-06_FarmB
A_black-headed_gull_Finland_8246_2023_H5N1_2023-07-20
A_arctic-fox_Finland_503_23_23VIR6803-6_2023_H5N1_2023-07-10_FarmE
A_raccoon-dog_Finland_555_2023_H5N1_2023-07-17_FarmI
A_arctic-fox_Finland_565_2023_H5N1_2023-07-17_FarmK
A_arctic-fox_Finland_670_2023_H5N1_2023-07-31_FarmX
A_black-headed_gull_Finland_8254_23VIR7026-19_2023_H5N1_2023-07-21
A_arctic-fox_Finland_738_2023_H5N1_2023-08-07_FarmE
A_raccoon-dog_Finland_623_2023_H5N1_2023-07-24_FarmT
A_arctic-fox_Finland_673_2023_H5N1_2023-08-01_FarmY
A_black-headed_gull_Finland_8247_2023_H5N1_2023-07-18
A_black-headed_gull_Finland_8095_2023_H5N1_2023-07-18
A_arctic-fox_Finland_496_2023_H5N1_2023-07-10_FarmD
A_raccoon-dog_Finland_625_2023_H5N1_2023-07-24_FarmT
A_arctic-fox_Finland_497_23_23VIR6803-5_2023_H5N1_2023-07-10_FarmD
A_arctic-fox_Finland_567_2023_H5N1_2023-07-17_FarmK
A_arctic-fox_Finland_675_2023_H5N1_2023-08-01_FarmY
A_arctic-fox_Finland_676_2023_H5N1_2023-08-01_FarmY
A_arctic-fox_Finland_677_2023_H5N1_2023-08-01_FarmY
A_arctic-fox_Finland_676_2023_H5N1_2023-08-01_FarmY
A_arctic-fox_Finland_674_2023_H5N1_2023-08-01_FarmY
A_black-headed_gull_Finland_6726_2023_H5N1_2023-06-13
A_arctic-fox_Finland_666_2023_H5N1_2023-07-31_FarmX
A_arctic-fox_Finland_660_2023_H5N1_2023-07-31_FarmW
A_black-headed_gull_Finland_8250_2023_H5N1_2023-07-20
A_arctic-fox_Finland_620_2023_H5N1_2023-07-24_FarmT
A_fox_Finland_610_2023_H5N1_2023-07-20_FarmO
A_black-headed_gull_Finland_8143_2023_H5N1_2023-07-19
A_black-headed_gull_Finland_9418_2023_H5N1_2023-08-10
A_arctic-fox_Finland_566_2023_H5N1_2023-07-17_FarmK
A_arctic-fox_Finland_576_2023_H5N1_2023-07-18_FarmL
A_arctic-fox_Finland_500_2023_H5N1_2023-07-10_FarmD
A_black-headed_gull_Austria_23030829-001_2023_H5N1_2023-03-08
A_black-headed_gull_Czech_Republic_5295-3_2023_H5N1_2023-04-03
A_black-headed_gull_Austria_23037203-001_2023_H5N1_2023-01-21
A_black-headed_gull_Austria_23031105-002_2023_H5N1_2023-03-08
A_black-headed_gull_Austria_23033899-002_2023_H5N1_2023-03-14
A_Vulpes_vulpes_Belgium_02359_0006_2023_H5N1_2023-03-14
A_Black-headed_gull_Netherlands_17_2023_H5N1_2023-03-05
A_Chroicocephalus_ridibundus_Belgium_00374_0005_2023_H5N1_2023-01-12
A_Vulpes_vulpes_Belgium_03117_0001_2023_H5N1_2023-03-29
A_Vulpes_vulpes_Belgium_01295_0002_2023_H5N1_2023-02-10
A_Vulpes_vulpes_Belgium_03117_0004_2023_H5N1_2023-03-29
A_black-headed_gull_Czech_Republic_6577-2_2023_H5N1_2023-04-24
A_black-headed_gull_Czech_Republic_6633-1_2023_H5N1_2023-04-24
A_black-headed_gull_Czech_Republic_6947-1_2023_H5N1_2023-05-03
A_black-headed_gull_Czech_Republic_7334-4_2023_H5N1_2023-05-11
A_domestic_duck_England_049343_2023_H5N1_2023-03-30
A_guinea_fowl_England_049422_2023_H5N1_2023-03-30
A_Chroicocephalus_ridibundus_Belgium_03706_0003_2023_H5N1_2023-04-18
A_Falco_peregrinus_Belgium_03518_0004_2023_H5N1_2023-04-11
A_Falco_peregrinus_Belgium_04695_0002_2023_H5N1_2023-05-07
A_Black-headed_gull_Netherlands_24_2023_H5N1_2023-03-09
A_Chroicocephalus_ridibundus_Belgium_03706_0002_2023_H5N1_2023-04-18
A_Falco_peregrinus_Belgium_01145_0005_2023_H5N1_2023-06-07
A_black-headed_gull_Spain_512-7_23VIR1687-7_2023_H5N1_2023-01-31
A_Black-headed_gull_Netherlands_8_2023_H5N1_2023-02-20
A_Gallus_gallus_Belgium_01742_0001_2023_H5N1_2023-02-27
A_fox_France_23P002844_2023_H5N1_2023-02-10
A_European_herring_gull_Netherlands_6_2023_H5N1_2023-02-20
A_Vulpes_Vulpes_Belgium_04016_0001_2023_H5N1_2023-04-24
A_black-headed_gull_Czech_Republic_5487-4_2023_H5N1_2023-04-12
A_Black-headed_Gull_Netherlands_95_2023_H5N1_2023-06-18
A_Falco_peregrinus_Belgium_02423_0004_2023_H5N1_2023-03-15
A_Falco_peregrinus_Belgium_01823_0003_2023_H5N1_2023-03-01
A_Mediterranean_Gull_Netherlands_3_2023_H5N1_2023-05-26
A_Black-headed_gull_Netherlands_39_2023_H5N1_2023-04-17
A_Black-headed_gull_Netherlands_40_2023_H5N1_2023-04-19
A_Chroicocephalus_ridibundus_Belgium_01145_0006_2023_H5N1_2023-02-07
A_Black-headed_Gull_Netherlands_73_2023_H5N1_2023-05-22
A_black-headed_gull_Spain_171-1_23VIR1687-20_2023_H5N1_2023-01-09
A_Falco_peregrinus_Belgium_01145_0004_2023_H5N1_2023-02-07
A_Vulpes_Vulpes_Belgium_03704_0001_2023_H5N1_2023-04-14
A_black-headed_gull_Austria_23056483-005_2023_H5N1_2023-04-27
A_Vulpes_vulpes_Belgium_01784_0008_2023_H5N1_2023-02-27
A_Pavo_cristatus_Belgium_02559_0001_2023_H5N1_2023-03-17
A_black-headed_gull_Italy_23VIR2383-2_2023_H5N1_2023-03-13
A_Vulpes_Vulpes_Belgium_03703_0001_2023_H5N1_2023-04-14
A_Chroicocephalus_ridibundus_Belgium_01145_0008_2023_H5N1_2023-02-07
A_Black-headed_Gull_Netherlands_77_2023_H5N1_2023-02-11
A_black-headed_gull_Austria_23056483-003_2023_H5N1_2023-04-27
A_black-headed_gull_Austria_23061383-001_2023_H5N1_2023-05-09
A_Vulpes_Vulpes_Belgium_03703_0007_2023_H5N1_2023-04-14
A_Vulpes_vulpes_Belgium_01924_0003_2023_H5N1_2023-03-02
A_Vulpes_vulpes_Belgium_02359_0002_2023_H5N1_2023-03-14
A_black-headed_gull_Czech_Republic_5695-4_2023_H5N1_2023-04-13
A_black-headed_gull_Austria_23052993-001_2023_H5N1_2023-04-20
A_fox_Italy_23VIR3885-1_2023_H5N1_2023-04-07
A_black-headed_gull_Austria_23025916-001_2023_H5N1_2023-02-28
A_black-headed_gull_Austria_23060170-003_2023_H5N1_2023-05-05
A_black-headed_gull_Czech_Republic_5487-2_2023_H5N1_2023-04-12
A_black-headed_gull_Czech_Republic_5695-3_2023_H5N1_2023-04-13
A_falcon_Austria_23032458_2023_H5N1_2023-03-10
A_black-headed_gull_Czech_Republic_5695-1_2023_H5N1_2023-04-13
A_black-headed_gull_Czech_Republic_5695-7_2023_H5N1_2023-04-13
A_black-headed_gull_Czech_Republic_5695-8_2023_H5N1_2023-04-13
A_black-headed_gull_Czech_Republic_5695-5_2023_H5N1_2023-04-13
A_black-headed_gull_Czech_Republic_6577-4_2023_H5N1_2023-04-24
A_black-headed_gull_Czech_Republic_6633-2_2023_H5N1_2023-04-24
A_Black-Headed_Gull_England_350132_2023_H5N1_2023-05-09
A_Anser_anser_Belgium_00286_0004_2023_H5N1_2023-01-02
A_Chroicocephalus_ridibundus_Belgium_00226_0001_2023_H5N1_2023-01-02
A_Black-headed_gull_Netherlands_18_2023_H5N1_2023-03-03
A_Black-headed_gull_Netherlands_19_2023_H5N1_2023-03-03
A_Chroicocephalus_ridibundus_Belgium_00132_0002_2022_H5N1_2022-12-28
A_black-headed_gull_Finland_7477_2023_H5N1_2023-06-25
A_common_tern_Finland_7479_2023_H5N1_2023-06-25
A_common_tern_Czech_Republic_8807-1_2023_H5N1_2023-05-05
A_common_tern_Czech_Republic_8807-5_2023_H5N1_2023-05-05
A_Vulpes_vulpes_Belgium_01784_0010_2023_H5N1_2023-02-27
A_Common_Tern_Netherlands_7_2023_H5N1_2023-06-03
A_Gallus_gallus_Belgium_02824_0001_2023_H5N1_2023-03-22
A_Gallus_gallus_Belgium_01694_0001_2023_H5N1_2023-02-24
A_Black-Headed_Gull_England_060002_2023_H5N1_2023-04-20
A_Reed_Warbler_England_125961_2023_H5N1_2023-05-16
A_Black-Headed_Gull_England_399997_2023_H5N1_2023-04-13
A_Peregrine_Falcon_England_260225_2023_H5N1_2023-05-17
A_Black-Headed_Gull_England_259880_2023_H5N1_2023-05-08
A_Black-Headed_Gull_England_259369_2023_H5N1_2023-04-18
A_Gallus_gallus_Belgium_01303_0001_2023_H5N1_2023-02-15
A_Gallus_gallus_Belgium_01172_0001_2023_H5N1_2023-02-06
A_Black-Headed_Gull_England_259311_2023_H5N1_2023-04-14
A_Black-headed_gull_Netherlands_28_2023_H5N1_2023-03-24
A_Black-Headed_Gull_England_259288_2023_H5N1_2023-04-14
A_Black-headed_Gull_Netherlands_86_2023_H5N1_2023-06-16
A_Black-headed_Gull_Netherlands_89_2023_H5N1_2023-06-16
A_Common_Tern_Netherlands_6_2023_H5N1_2023-05-31
A_Black-headed_gull_Netherlands_87_2023_H5N1_2023-06-16
A_raxorbill_Finland_9072_2023_H5N1_2023-07-27
A_black-headed_gull_Finland_LI7726_23_23VIR6803-9_2023_H5N1_2023-07-03
A_canada_goose_Finland_8109_23VIR7026-20_2023_H5N1_2023-07-18
A_great_crested_grebe_Finland_8111_23VIR7026-21_2023_H5N1_2023-07-18
A_Black-Headed_Gull_England_260252_2023_H5N1_2023-05-17
A_Black-Headed_Gull_England_400845_2023_H5N1_2023-05-13
A_Common_Tern_England_260258_2023_H5N1_2023-05-17
A_black-headed_gull_Netherlands_37_2023_H5N1_2023-04-17
A_black-headed_gull_Netherlands_38_2023_H5N1_2023-04-17
A_Common_Tern_Netherlands_9_2023_H5N1_2023-06-02
A_mink_Finland_3076_2023_H5N1_2023-09-20_FarmAA
A_mink_Finland_3079_2023_H5N1_2023-09-20_FarmAA
A_mink_Finland_3078_2023_H5N1_2023-09-20_FarmAA
A_mink_Finland_3081_2023_H5N1_2023-09-20_FarmAA
A_mink_Finland_3082_2023_H5N1_2023-09-20_FarmAA
A_mink_Finland_3080_2023_H5N1_2023-09-20_FarmAA
A_mink_Finland_3077_2023_H5N1_2023-09-20_FarmAA
A_black-headed_gull_Finland_9079_2023_H5N1_2023-07-28
A_herring_gull_Finland_10812_2023_H5N1_2023-09-18
A_Caspian_gull_Netherlands_4_2023_H5N1_2023-06-09
A_Black-headed_Gull_Netherlands_101_2023_H5N1_2023-06-23
A_Black-headed_Gull_Netherlands_103_2023_H5N1_2023-06-26
A_European_herring_gull_Netherlands_11_2023_H5N1_2023-03-27
A_Black-Headed_Gull_England_259616_2023_H5N1_2023-04-25
A_Peregrine_falcon_Netherlands_6_2023_H5N1_2023-02-07
A_European_herring_gull_Netherlands_8_2023_H5N1_2023-03-03
A_herring_gull_Finland_10450_2023_H5N1_2023-08-29
A_black-legged_kittiwake_Finland_8808_2023_H5N1_2023-07-28
A_black-legged_kittiwake_Finland_8772_2023_H5N1_2023-07-23
A_Peregrine_Falcon_Netherlands_13_2023_H5N1_2023-03-10
A_Peregrine_Falcon_Netherlands_12_2023_H5N1_2023-03-10
A_white-tailed_eagle_Finland_10644_2023_H5N1_2023-09-02
A_white-tailed_eagle_Finland_8154_23VIR7026-22_2023_H5N1_2023-07-18
A_black-headed_gull_Finland_8107_23VIR7026-7_2023_H5N1_2023-07-20
A_white-tailed_eagle_Finland_LI7800_23_23VIR6803-10_2023_H5N1_2023-06-28
A_mute_swan_Denmark_06134-1.02_2023-07-27_H5N1_2023-07-27
A_mute_swan_Denmark_06339-1.01_2023-08-05_H5N1_2023-08-05
A_Chicken_England_155942_2022_H5N1_2022-11-20
A_turkey_England_132460_2022_H5N1_2022-10-15
A_canada_goose_England_396207_2022_H5N1_2022-12-10
A_Fox_Netherlands_EMC7_2022_H5N1_2022-09-13
A_chicken_Wales_023780_2023_H5N1_2023-02-11
A_Bush_dog_England_037680_2022_H5N1_2022-11-18
H5N1 Finland 2021
H5N8 Finland 2021
A_Mulard_Duck_Hungary_59163_2016_H5N8
A_Tufted_duck_Denmark_11740-LWPL_2016_H5N8
A_turkey_Israel_1045_2016_H5N8
A_Turkey_Egypt_AR550_2018_H5N8
A_chicken_Egypt_M14081D_2017_H5N8
0.002

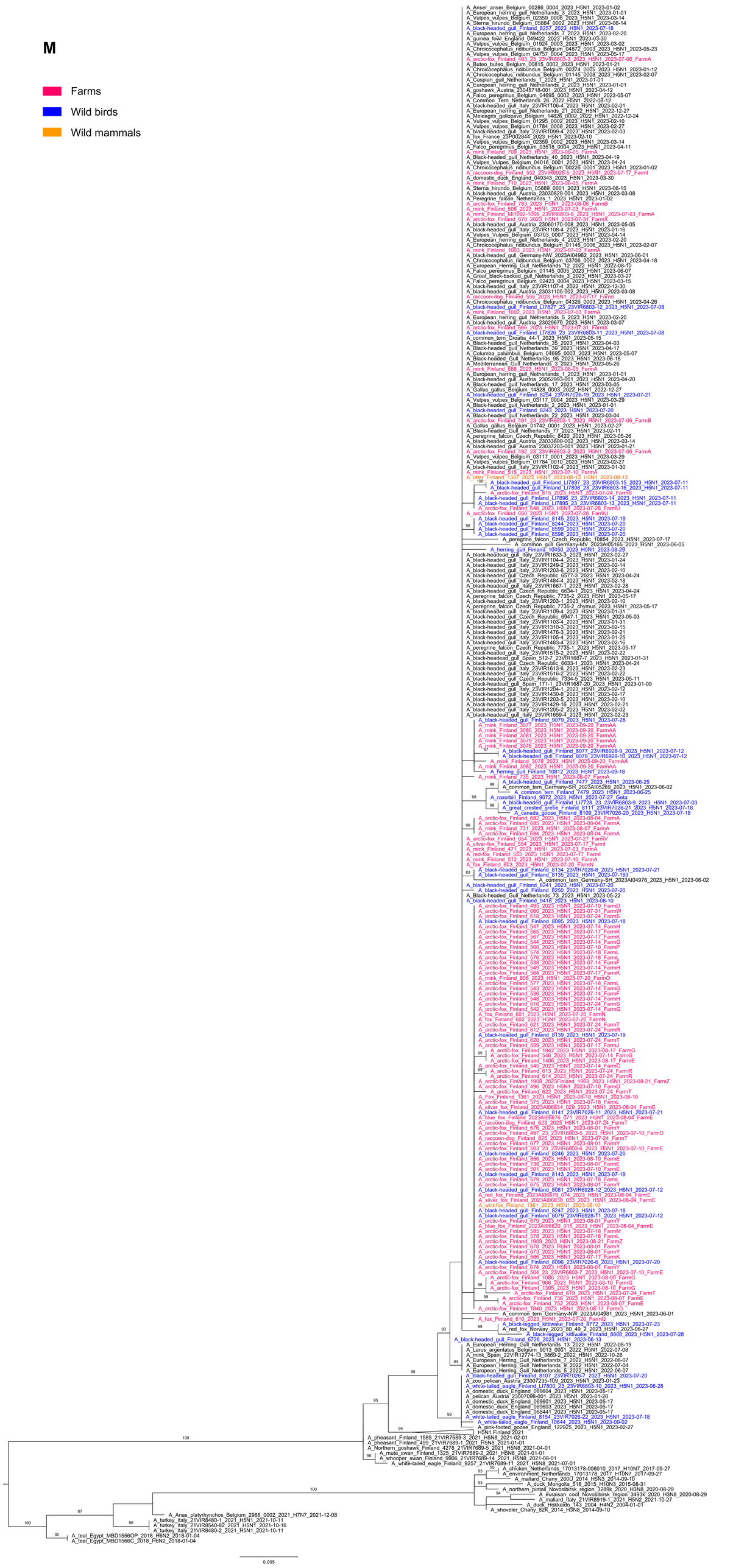
M
Farms
Wild birds
Wild mammals
A_Anser_anser_Belgium_00286_0004_2023_H5N1_2023-01-02
A_European_herring_gull_Netherlands_3_2023_H5N1_2023-01-01
A_Vulpes_vulpes_Belgium_02359_0006_2023_H5N1_2023-03-14
A_Sterna_hirundo_Belgium_05884_0002_2023_H5N1_2023-06-14
A_black-headed_gull_Finland_8257_2023_H5N1_2023-07-18
A_European_herring_gull_Netherlands_7_2023_H5N1_2023-02-20
A_guinea_fowl_England_049422_2023_H5N1_2023-03-30
A_Vulpes_vulpes_Belgium_01924_0003_2023_H5N1_2023-03-02
A_Choricocephalus_ridibundus_Belgium_04872_0003_2023_H5N1_2023-05-23
A_Vulpes_vulpes_Belgium_04757_0004_2023_H5N1_2023-05-17
A_arctic-fox_Finland_493_23_23VIR6843-5_2023_H5N1_2023-07-06_FarmA
A_Buteo_buteo_Belgium_00815_0002_2023_H5N1_2023-01-21
A_Choricocephalus_ridibundus_Belgium_00374_0005_2023_H5N1_2023-01-12
A_Choricocephalus_ridibundus_Belgium_01145_0008_2023_H5N1_2023-02-07
A_Caspian_gull_Netherlands_1_2023_H5N1_2023-01-01
A_European_herring_gull_Netherlands_2_2023_H5N1_2023-01-01
A_goshawk_Austria_23048718-001_2023_H5N1_2023-04-12
A_Falco_peregrinus_Belgium_04695_0002_2023_H5N1_2023-05-07
A_Common_Tern_Netherlands_26_2022_H5N1_2022-08-12
A_black-headed_gull_Italy_23VIR1106-4_2023_H5N1_2023-02-01
A_European_herring_gull_Netherlands_21_2022_H5N1_2022-12-27
A_Meleagris_gallopavo_Belgium_14828_0002_2022_H5N1_2022-12-24
A_Vulpes_vulpes_Belgium_01295_0002_2023_H5N1_2023-02-10
A_Vulpes_vulpes_Belgium_01784_0008_2023_H5N1_2023-02-27
A_black-headed_gull_Italy_23VIR1099-4_2023_H5N1_2023-02-03
A_fox_France_23P002844_2023_H5N1_2023-02-10
A_Vulpes_vulpes_Belgium_02359_0002_2023_H5N1_2023-03-14
A_Falco_peregrinus_Belgium_03518_0004_2023_H5N1_2023-04-11
A_mink_Finland_705_2023_H5N1_2023-08-05_FarmA
A_Black-headed_gull_Netherlands_40_2023_H5N1_2023-04-19
A_Vulpes_Vulpes_Belgium_04016_0001_2023_H5N1_2023-04-24
A_Choricocephalus_ridibundus_Belgium_00226_0001_2023_H5N1_2023-01-02
A_raccoon-dog_Finland_552_23VIR6928-5_2023_H5N1_2023-07-17_FarmI
A_domestic_duck_England_049343_2023_H5N1_2023-03-30
A_mink_Finland_710_2023_H5N1_2023-08-05_FarmA
A_Sterna_hirundo_Belgium_05889_0001_2023_H5N1_2023-06-15
A_black-headed_gull_Austria_23030829-001_2023_H5N1_2023-03-08
A_Peregrine_falcon_Netherlands_1_2023_H5N1_2023-01-02
A_arctic-fox_Finland_783_2023_H5N1_2023-08-08_FarmB
A_mink_Finland_506_2023_H5N1_2023-07-03_FarmA
A_mink_Finland_MI1002-1006_23VIR6803-8_2023_H5N1_2023-07-03_FarmA
A_arctic-fox_Finland_670_2023_H5N1_2023-07-31_FarmK
A_black-headed_gull_Austria_23060170-008_2023_H5N1_2023-05-05
A_black-headed_gull_Italy_23VIR1108-4_2023_H5N1_2023-01-16
A_Vulpes_Vulpes_Belgium_03703_0007_2023_H5N1_2023-04-14
A_European_herring_gull_Netherlands_4_2023_H5N1_2023-02-20
A_Choricocephalus_ridibundus_Belgium_01145_0006_2023_H5N1_2023-02-07
A_mink_Finland_1003_2023_H5N1_2023-07-03_FarmA
A_black-headed_gull_Germany-NW_2023AI04982_2023_H5N1_2023-06-01
A_Choricocephalus_ridibundus_Belgium_03706_0002_2023_H5N1_2023-04-18
A_European_Herring_Gull_Netherlands_12_2022_H5N1_2022-08-10
A_Falco_peregrinus_Belgium_01145_0005_2023_H5N1_2023-06-07
A_Great_black-backed_gull_Netherlands_3_2023_H5N1_2023-03-27
A_Falco_peregrinus_Belgium_02423_0004_2023_H5N1_2023-03-15
A_black-headed_gull_Italy_23VIR1107-4_2022_H5N1_2022-12-30
A_black-headed_gull_Austria_23031105-002_2023_H5N1_2023-03-08
A_raccoon-dog_Finland_555_2023_H5N1_2023-07-17_FarmI
A_Choricocephalus_ridibundus_Belgium_04326_0003_2023_H5N1_2023-04-28
A_black-headed_gull_Finland_LI7827_23_23VIR6803-12_2023_H5N1_2023-07-08
A_mink_Finland_1002_2023_H5N1_2023-07-03_FarmA
A_European_herring_gull_Netherlands_5_2023_H5N1_2023-02-20
A_black-headed_gull_Austria_23029679_2023_H5N1_2023-03-07
A_arctic-fox_Finland_666_2023_H5N1_2023-07-31_FarmX
A_black-headed_gull_Finland_LI7836_23_23VIR6803-11_2023_H5N1_2023-07-08
A_common_tern_Croatia_44-1_2023_H5N1_2023-05-15
A_Black-headed_gull_Netherlands_35_2023_H5N1_2023-04-03
A_Black-headed_gull_Netherlands_39_2023_H5N1_2023-04-17
A_Columba_palumbus_Belgium_04695_0003_2023_H5N1_2023-05-07
A_Black-headed_gull_Netherlands_95_2023_H5N1_2023-06-18
A_Mediterranean_Gull_Netherlands_3_2023_H5N1_2023-05-26
A_mink_Finland_888_2023_H5N1_2023-08-05_FarmA
A_European_herring_gull_Netherlands_1_2023_H5N1_2023-01-01
A_black-headed_gull_Austria_23052993-001_2023_H5N1_2023-04-20
A_Black-headed_gull_Netherlands_17_2023_H5N1_2023-03-05
A_Gallus_gallus_Belgium_14828_0003_2022_H5N1_2022-12-27
A_black-headed_gull_Finland_8254_23VIR7026-19_2023_H5N1_2023-07-21
A_Vulpes_vulpes_Belgium_03117_0004_2023_H5N1_2023-03-29
A_Black-headed_gull_Netherlands_2_2023_H5N1_2023-01-01
A_black-headed_gull_Finland_8243_2023_H5N1_2023-07-20
A_Black-headed_gull_Netherlands_22_2023_H5N1_2023-03-04
A_arctic-fox_Finland_491_23_23VIR6803-1_2023_H5N1_2023-07-06_FarmB
A_Gallus_gallus_Belgium_01742_0001_2023_H5N1_2023-02-27
A_Black-headed_Gull_Netherlands_77_2023_H5N1_2023-02-11
A_peregrine_falcon_Czech_Republic_8420_2023_H5N1_2023-05-26
A_black-headed_gull_Austria_23033899-002_2023_H5N1_2023-03-14
A_black-headed_gull_Austria_23037203-001_2023_H5N1_2023-01-21
A_arctic-fox_Finland_492_23_23VIR6803-2_2023_H5N1_2023-07-06_FarmA
A_Vulpes_vulpes_Belgium_03117_0001_2023_H5N1_2023-03-29
A_Vulpes_vulpes_Belgium_01784_0010_2023_H5N1_2023-02-27
A_black-headed_gull_Italy_23VIR1102-4_2023_H5N1_2023-01-30
A_mink_Finland_515_2023_H5N1_2023-07-10_FarmA
A_otter_Finland_1357_2023_H5N1_2023-09-13_H5N1_2023-08-13
A_black-headed_gull_Finland_LI7897_23_23VIR6803-15_2023_H5N1_2023-07-11
A_black-headed_gull_Finland_LI7898_23_23VIR6803-16_2023_H5N1_2023-07-11
A_arctic-fox_Finland_815_2023_H5N1_2023-07-24_FarmS
A_black-headed_gull_Finland_LI7896_23_23VIR6803-14_2023_H5N1_2023-07-11
A_black-headed_gull_Finland_LI7895_23_23VIR6803-13_2023_H5N1_2023-07-11
A_arctic-fox_Finland_848_2023_H5N1_2023-07-28_FarmU
A_arctic-fox_Finland_650_2023_H5N1_2023-07-28_FarmU
A_black-headed_gull_Finland_8145_2023_H5N1_2023-07-19
A_black-headed_gull_Finland_8244_2023_H5N1_2023-07-20
A_black-headed_gull_Finland_8599_2023_H5N1_2023-07-20
A_black-headed_gull_Finland_8598_2023_H5N1_2023-07-20
A_peregrine_falcon_Czech_Republic_10854_2023_H5N1_2023-07-17
A_common_gull_Germany-MV_2023AI05165_2023_H5N1_2023-06-05
A_herring_gull_Finland_10450_2023_H5N1_2023-08-09
A_black-headed_gull_Italy_23VIR1633-3_2023_H5N1_2023-02-27
A_black-headed_gull_Italy_23VIR1104-4_2023_H5N1_2023-01-24
A_black-headed_gull_Italy_23VIR1249-2_2023_H5N1_2023-02-14
A_black-headed_gull_Italy_23VIR1203-6_2023_H5N1_2023-02-10
A_black-headed_gull_Czech_Republic_6577-3_2023_H5N1_2023-04-24
A_black-headed_gull_Italy_23VIR1484-4_2023_H5N1_2023-02-18
A_black-headed_gull_Italy_23VIR1667-1_2023_H5N1_2023-02-28
A_black-headed_gull_Czech_Republic_6634-1_2023_H5N1_2023-04-24
A_peregrine_falcon_Czech_Republic_7735-2_2023_H5N1_2023-05-17
A_black-headed_gull_Italy_23VIR1203-1_2023_H5N1_2023-02-10
A_peregrine_falcon_Czech_Republic_7735-2_chymus_2023_H5N1_2023-05-17
A_black-headed_gull_Italy_23VIR1109-4_2023_H5N1_2023-01-31
A_black-headed_gull_Czech_Republic_6947-1_2023_H5N1_2023-05-03
A_black-headed_gull_Italy_23VIR1103-4_2023_H5N1_2023-01-31
A_black-headed_gull_Italy_23VIR1310-3_2023_H5N1_2023-02-15
A_black-headed_gull_Italy_23VIR1476-3_2023_H5N1_2023-02-21
A_black-headed_gull_Italy_23VIR1105-4_2023_H5N1_2023-01-25
A_black-headed_gull_Italy_23VIR1483-4_2023_H5N1_2023-02-16
A_peregrine_falcon_Czech_Republic_7736-1_2023_H5N1_2023-05-17
A_black-headed_gull_Italy_23VIR1515-2_2023_H5N1_2023-02-22
A_black-headed_gull_Spain_512-7_23VIR1687-7_2023_H5N1_2023-01-31
A_black-headed_gull_Czech_Republic_6633-1_2023_H5N1_2023-04-24
A_black-headed_gull_Italy_23VIR1613-8_2023_H5N1_2023-02-23
A_black-headed_gull_Italy_23VIR1516-2_2023_H5N1_2023-02-22
A_black-headed_gull_Czech_Republic_7334-5_2023_H5N1_2023-05-11
A_black-headed_gull_Spain_171-1_23VIR1687-20_2023_H5N1_2023-01-09
A_black-headed_gull_Italy_23VIR1204-1_2023_H5N1_2023-02-12
A_black-headed_gull_Italy_23VIR1430-8_2023_H5N1_2023-02-17
A_black-headed_gull_Italy_23VIR1203-5_2023_H5N1_2023-02-10
A_black-headed_gull_Italy_23VIR1429-16_2023_H5N1_2023-02-21
A_black-headed_gull_Italy_23VIR1205-2_2023_H5N1_2023-02-02
A_black-headed_gull_Italy_23VIR1659-4_2023_H5N1_2023-02-23
A_black-headed_gull_Finland_9079_2023_H5N1_2023-07-28
A_mink_Finland_3077_2023_H5N1_2023-09-20_FarmAA
A_mink_Finland_3080_2023_H5N1_2023-09-20_FarmAA
A_mink_Finland_3081_2023_H5N1_2023-09-20_FarmAA
A_mink_Finland_3079_2023_H5N1_2023-09-20_FarmAA
A_mink_Finland_3076_2023_H5N1_2023-09-20_FarmAA
A_black-headed_gull_Finland_8077_23VIR6928-9_2023_H5N1_2023-07-12
A_black-headed_gull_Finland_8078_23VIR6928-10_2023_H5N1_2023-07-12
A_mink_Finland_3078_2023_H5N1_2023-09-20_FarmAA
A_mink_Finland_3082_2023_H5N1_2023-09-20_FarmAA
A_herring_gull_Finland_10812_2023_H5N1_2023-09-18
A_mink_Finland_735_2023_H5N1_2023-08-07_FarmA
A_black-headed_gull_Finland_7477_2023_H5N1_2023-06-25
A_common_tern_Germany-SH_2023AI05269_2023_H5N1_2023-06-02
A_common_tern_Finland_7479_2023_H5N1_2023-06-25
A_raxorbill_Finland_9072_2023_H5N1_2023-07-27_Geta
A_black-headed_gull_Finland_LI7728_23_23VIR6803-9_2023_H5N1_2023-07-03
A_great_crested_grebe_Finland_8111_23VIR7026-21_2023_H5N1_2023-07-18
A_canada_goose_Finland_8109_23VIR7026-20_2023_H5N1_2023-07-18
A_arctic-fox_Finland_682_2023_H5N1_2023-08-04_FarmA
A_arctic-fox_Finland_685_2023_H5N1_2023-08-04_FarmA
A_mink_Finland_731_2023_H5N1_2023-08-07_FarmA
A_arctic-fox_Finland_684_2023_H5N1_2023-08-04_FarmA
A_arctic-fox_Finland_654_2023_H5N1_2023-07-27_FarmV
A_silver-fox_Finland_554_2023_H5N1_2023-07-17_FarmI
A_mink_Finland_471_2023_H5N1_2023-07-03_FarmA
A_red-fox_Finland_653_2023_H5N1_2023-07-17_FarmI
A_mink_Finland_512_2023_H5N1_2023-07-10_FarmA
A_fox_Finland_603_2023_H5N1_2023-07-20_FarmN
A_black-headed_gull_Finland_8134_23VIR7026-4_2023_H5N1_2023-07-21
A_black-headed_gull_Finland_8135_2023_H5N1_2023-07-193
A_common_tern_Germany-SH_2023AI04976_2023_H5N1_2023-06-02
A_black-headed_gull_Finland_8241_2023_H5N1_2023-07-20
A_black-headed_gull_Finland_8250_2023_H5N1_2023-07-20
A_Black-headed_Gull_Netherlands_73_2023_H5N1_2023-05-22
A_black-headed_gull_Finland_9418_2023_H5N1_2023-08-10
A_arctic-fox_Finland_495_2023_H5N1_2023-07-10_FarmD
A_arctic-fox_Finland_660_2023_H5N1_2023-07-31_FarmW
A_arctic-fox_Finland_618_2023_H5N1_2023-07-24_FarmS
A_black-headed_gull_Finland_8095_2023_H5N1_2023-07-18
A_arctic-fox_Finland_547_2023_H5N1_2023-07-14_FarmH
A_arctic-fox_Finland_565_2023_H5N1_2023-07-17_FarmK
A_arctic-fox_Finland_567_2023_H5N1_2023-07-17_FarmK
A_arctic-fox_Finland_544_2023_H5N1_2023-07-14_FarmG
A_arctic-fox_Finland_500_2023_H5N1_2023-07-10_FarmP
A_arctic-fox_Finland_574_2023_H5N1_2023-07-18_FarmL
A_arctic-fox_Finland_576_2023_H5N1_2023-07-18_FarmL
A_arctic-fox_Finland_536_2023_H5N1_2023-07-14_FarmF
A_arctic-fox_Finland_549_2023_H5N1_2023-07-14_FarmH
A_arctic-fox_Finland_564_2023_H5N1_2023-07-17_FarmK
A_mink_Finland_606_2023_H5N1_2023-07-20_FarmO
A_arctic-fox_Finland_577_2023_H5N1_2023-07-18_FarmL
A_arctic-fox_Finland_543_2023_H5N1_2023-07-14_FarmG
A_arctic-fox_Finland_536_2023_H5N1_2023-07-14_FarmF
A_arctic-fox_Finland_548_2023_H5N1_2023-07-14_FarmH
A_arctic-fox_Finland_616_2023_H5N1_2023-07-24_FarmS
A_arctic-fox_Finland_542_2023_H5N1_2023-07-14_FarmG
A_fox_Finland_601_2023_H5N1_2023-07-20_FarmN
A_fox_Finland_602_2023_H5N1_2023-07-20_FarmN
A_arctic-fox_Finland_621_2023_H5N1_2023-07-24_FarmT
A_arctic-fox_Finland_612_2023_H5N1_2023-07-24_FarmR
A_black-headed_gull_Finland_8139_2023_H5N1_2023-07-19
A_arctic-fox_Finland_620_2023_H5N1_2023-07-24_FarmT
A_arctic-fox_Finland_559_2023_H5N1_2023-07-17_FarmJ
A_arctic-fox_Finland_1842_2023_H5N1_2023-08-17_FarmG
A_arctic-fox_Finland_546_2023_H5N1_2023-07-14_FarmG
A_arctic-fox_Finland_1406_2023_H5N1_2023-08-17_FarmE
A_arctic-fox_Finland_545_2023_H5N1_2023-07-14_FarmG
A_arctic-fox_Finland_613_2023_H5N1_2023-07-24_FarmR
A_arctic-fox_Finland_614_2023_H5N1_2023-07-24_FarmR
A_arctic-fox_Finland_1908_2023Finland_1908_2023_H5N1_2023-08-21_FarmZ
A_arctic-fox_Finland_496_2023_H5N1_2023-07-10_FarmD
A_arctic-fox_Finland_622_2023_H5N1_2023-07-24_FarmT
A_Fox_Finland_1361_2023_H5N1_2023-08-10_H5N1_2023-08-10
A_arctic-fox_Finland_575_2023_H5N1_2023-07-18_FarmL
A_silver_fox_Finland_2023AI06834_029_2023_H5N1_2023-08-04_FarmE
A_black-headed_gull_Finland_8141_23VIR7026-11_2023_H5N1_2023-07-21
A_blue_fox_Finland_2023AI06878_071_2023_H5N1_2023-08-04_FarmE
A_raccoon-dog_Finland_623_2023_H5N1_2023-07-24_FarmT
A_arctic-fox_Finland_678_2023_H5N1_2023-08-01_FarmY
A_arctic-fox_Finland_497_23_23VIR6803-9_2023_H5N1_2023-07-10_FarmD
A_raccoon-dog_Finland_625_2023_H5N1_2023-07-24_FarmT
A_arctic-fox_Finland_677_2023_H5N1_2023-08-01_FarmY
A_arctic-fox_Finland_503_23_23VIR6803-4_2023_H5N1_2023-07-10_FarmE
A_black-headed_gull_Finland_8246_2023_H5N1_2023-07-20
A_arctic-fox_Finland_856_2023_H5N1_2023-08-10_FarmE
A_arctic-fox_Finland_738_2023_H5N1_2023-08-07_FarmE
A_arctic-fox_Finland_501_2023_H5N1_2023-07-10_FarmE
A_black-headed_gull_Finland_8143_2023_H5N1_2023-07-19
A_arctic-fox_Finland_579_2023_H5N1_2023-07-18_FarmL
A_arctic-fox_Finland_675_2023_H5N1_2023-08-01_FarmY
A_black-headed_gull_Finland_8081_23VIR6928-12_2023_H5N1_2023-07-12
A_red_fox_Finland_2023AI06879_074_2023_H5N1_2023-08-04_FarmE
A_silver_fox_Finland_2023AI06838_033_2023_H5N1_2023-08-04_FarmE
A_wild-fox_Finland_1361_2023_H5N1_2023-08-10
A_black-headed_gull_Finland_8247_2023_H5N1_2023-07-18
A_black-headed_gull_Finland_8079_23VIR6928-11_2023_H5N1_2023-07-12
A_arctic-fox_Finland_679_2023_H5N1_2023-08-01_FarmY
A_blue_fox_Finland_2023AI06820_015_2023_H5N1_2023-08-04_FarmE
A_arctic-fox_Finland_585_2023_H5N1_2023-07-18_FarmM
A_arctic-fox_Finland_576_2023_H5N1_2023-07-18_FarmL
A_arctic-fox_Finland_1909_2023_H5N1_2023-08-21_FarmZ
A_arctic-fox_Finland_678_2023_H5N1_2023-08-01_FarmY
A_arctic-fox_Finland_673_2023_H5N1_2023-08-01_FarmY
A_arctic-fox_Finland_566_2023_H5N1_2023-07-17_FarmK
A_black-headed_gull_Finland_8096_23VIR7026-6_2023_H5N1_2023-07-20
A_arctic-fox_Finland_674_2023_H5N1_2023-08-01_FarmY
A_arctic-fox_Finland_504_23_23VIR6803-7_2023_H5N1_2023-07-10_FarmE
A_arctic-fox_Finland_1086_2023_H5N1_2023-08-09_FarmG
A_arctic-fox_Finland_968_2023_H5N1_2023-08-10_FarmG
A_arctic-fox_Finland_1305_2023_H5N1_2023-08-10_FarmG
A_arctic-fox_Finland_619_2023_H5N1_2023-07-24_FarmT
A_arctic-fox_Finland_736_2023_H5N1_2023-08-07_FarmE
A_arctic-fox_Finland_752_2023_H5N1_2023-08-07_FarmE
A_arctic-fox_Finland_1840_2023_H5N1_2023-08-17_FarmG
A_common_tern_Germany-NW_2023AI04981_2023_H5N1_2023-06-01
A_fox_Finland_610_2023_H5N1_2023-07-20_FarmO
A_black-legged_kittiwake_Finland_8772_2023_H5N1_2023-07-23
A_red_fox_Norway_2023_80_49_2_2023_H5N1_2023-06-27
A_black-legged_kittiwake_Finland_8808_2023_H5N1_2023-07-28
A_black-headed_gull_Finland_6726_2023_H5N1_2023-06-13
A_European_Herring_Gull_Netherlands_13_2022_H5N1_2022-08-19
A_Larus_argentatus_Belgium_9013_0001_2022_H5N1_2022-07-08
A_mink_Spain_22VIR12774-13_3869-2_2022_H5N1_2022-10-26
A_European_Herring_Gull_Netherlands_7_2022_H5N1_2022-06-07
A_European_Herring_Gull_Netherlands_9_2022_H5N1_2022-07-04
A_European_Herring_Gull_Netherlands_5_2022_H5N1_2022-06-07
A_black-headed_gull_Finland_8107_23VIR7026-7_2023_H5N1_2023-07-20
A_zoo_pelican_Austria_23007235-109_2023_H5N1_2023-01-23
A_white-tailed_eagle_Finland_LI7800_23_23VIR6803-10_2023_H5N1_2023-06-28
A_domestic_duck_England_069604_2023_H5N1_2023-05-17
A_pelican_Austria_23007098-001_2023_H5N1_2023-01-20
A_domestic_duck_England_069601_2023_H5N1_2023-05-17
A_domestic_duck_England_069603_2023_H5N1_2023-05-17
A_domestic_duck_England_069441_2023_H5N1_2023-05-17
A_white-tailed_eagle_Finland_8154_23VIR7026-22_2023_H5N1_2023-07-18
A_white-tailed_eagle_Finland_10644_2023_H5N1_2023-09-02
A_pink-footed_goose_England_122925_2023_H5N1_2023-02-27
H5N1 Finland 2021
A_pheasant_Finland_1589_21VIR7689-3_2021_H5N8_2021-02-01
A_pheasant_Finland_499_21VIR7689-1_2021_H5N8_2021-01-01
A_Northern_goshawk_Finland_4278_21VIR7689-5_2021_H5N8_2021-04-01
A_mute_swan_Finland_1325_21VIR7689-2_2021_H5N8_2021-01-01
A_whooper_swan_Finland_9906_21VIR7689-14_2021_H5N8_2021-09-01
A_white-tailed_eagle_Finland_9257_21VIR7689-11_2021_H5N8_2021-07-01
A_chicken_Netherlands_17013178-006010_2017_H10N7_2017-09-27
A_environment_Netherlands_17013178_2017_H10N7_2017-09-27
A_mallard_Chany_260D_2014_H5N3_2014-09-10
A_duck_Mongolia_518_2015_H10N3_2015-08-31
A_northern_pintail_Novosibirsk_region_3289k_2020_H3N8_2020-08-29
A_eurasian_coot_Novosibirsk_region_3493k_2020_H3N8_2020-08-29
A_mallard_Italy_21VIR8919-1_2021_H5N2_2021-10-27
A_duck_Hokkaido_143_2004_H4N2_2004-01-01
A_shoveler_Chany_82K_2014_H3N8_2014-09-10
A_Anas_platyrhynchos_Belgium_2988_0002_2021_H7N7_2021-12-08
A_turkey_Italy_21VIR8480-1_2021_H5N1_2021-10-11
A_turkey_Italy_21VIR8540-82_2021_H5N1_2021-10-16
A_turkey_Italy_21VIR8480-2_2021_H5N1_2021-10-11
A_teal_Egypt_MBD1556OP_2018_H6N2_2018-01-04
A_teal_Egypt_MBD1566C_2018_H6N2_2018-01-04
0.005

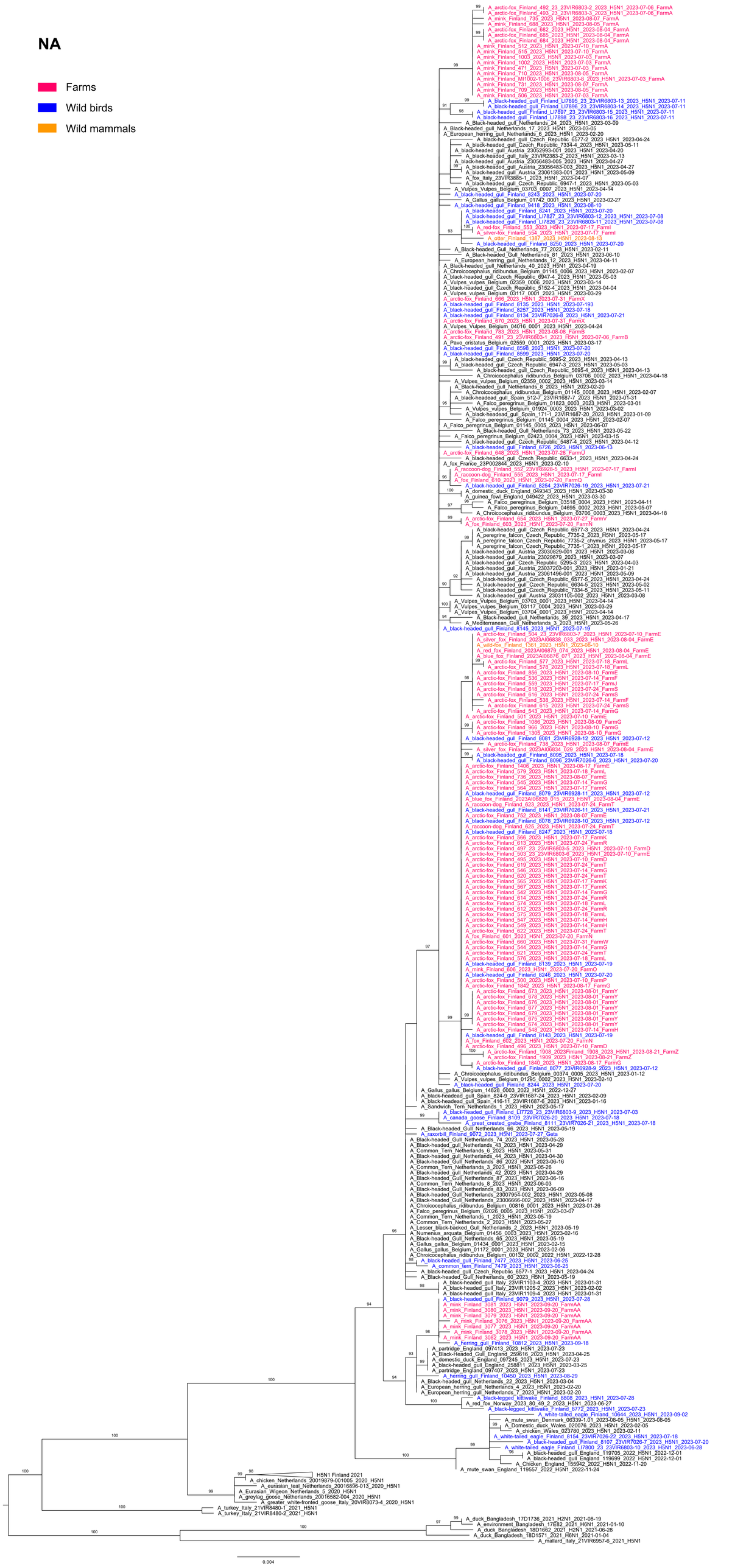
NA
Farms
Wild birds
Wild mammals
A_arctic-fox_Finland_492_23_23VIR6803-2_2023_H5N1_2023-07-06_FarmA
A_arctic-fox_Finland_493_23_23VIR6803-3_2023_H5N1_2023-07-06_FarmA
A_mink_Finland_735_2023_H5N1_2023-08-07_FarmA
A_mink_Finland_688_2023_H5N1_2023-08-05_FarmA
A_arctic-fox_Finland_682_2023_H5N1_2023-08-04_FarmA
A_arctic-fox_Finland_685_2023_H5N1_2023-08-04_FarmA
A_arctic-fox_Finland_684_2023_H5N1_2023-08-04_FarmA
A_mink_Finland_512_2023_H5N1_2023-07-10_FarmA
A_mink_Finland_515_2023_H5N1_2023-07-10_FarmA
A_mink_Finland_1003_2023_H5N1_2023-07-03_FarmA
A_mink_Finland_1002_2023_H5N1_2023-07-03_FarmA
A_mink_Finland_471_2023_H5N1_2023-07-03_FarmA
A_mink_Finland_710_2023_H5N1_2023-08-05_FarmA
A_mink_Finland_MI1002-1006_23VIR6803-8_2023_H5N1_2023-07-03_FarmA
A_mink_Finland_731_2023_H5N1_2023-08-07_FarmA
A_mink_Finland_709_2023_H5N1_2023-08-05_FarmA
A_mink_Finland_506_2023_H5N1_2023-07-03_FarmA
A_black-headed_gull_Finland_LI7895_23_23VIR6803-13_2023_H5N1_2023-07-11
A_black-headed_gull_Finland_LI7896_23_23VIR6803-14_2023_H5N1_2023-07-11
A_black-headed_gull_Finland_LI7897_23_23VIR6803-15_2023_H5N1_2023-07-11
A_black-headed_gull_Finland_LI7898_23_23VIR6803-16_2023_H5N1_2023-07-11
A_Black-headed_gull_Netherlands_24_2023_H5N1_2023-03-09
A_Black-headed_gull_Netherlands_17_2023_H5N1_2023-03-05
A_European_herring_gull_Netherlands_6_2023_H5N1_2023-02-20
A_black-headed_gull_Czech_Republic_6577-2_2023_H5N1_2023-04-24
A_black-headed_gull_Czech_Republic_7334-4_2023_H5N1_2023-05-11
A_black-headed_gull_Austria_23052993-001_2023_H5N1_2023-04-20
A_black-headed_gull_Italy_23VIR2383-2_2023_H5N1_2023-03-13
A_black-headed_gull_Austria_23056483-005_2023_H5N1_2023-04-27
A_black-headed_gull_Austria_23056483-003_2023_H5N1_2023-04-27
A_black-headed_gull_Austria_23061383-001_2023_H5N1_2023-05-09
A_fox_Italy_23VIR3885-1_2023_H5N1_2023-04-07
A_black-headed_gull_Czech_Republic_6947-1_2023_H5N1_2023-05-03
A_Vulpes_Vulpes_Belgium_03703_0007_2023_H5N1_2023-04-14
A_black-headed_gull_Finland_8243_2023_H5N1_2023-07-20
A_Gallus_gallus_Belgium_01742_0001_2023_H5N1_2023-02-27
A_black-headed_gull_Finland_9418_2023_H5N1_2023-08-10
A_black-headed_gull_Finland_8241_2023_H5N1_2023-07-20
A_black-headed_gull_Finland_LI7827_23_23VIR6803-12_2023_H5N1_2023-07-08
A_black-headed_gull_Finland_LI7826_23_23VIR6803-11_2023_H5N1_2023-07-08
A_red-fox_Finland_553_2023_H5N1_2023-07-17_FarmI
A_silver-fox_Finland_554_2023_H5N1_2023-07-17_FarmI
A_otter_Finland_1387_2023_H5N1_2023-08-15
A_black-headed_gull_Finland_8250_2023_H5N1_2023-07-20
A_Black-headed_Gull_Netherlands_77_2023_H5N1_2023-02-11
A_Black-headed_Gull_Netherlands_81_2023_H5N1_2023-06-10
A_European_herring_gull_Netherlands_12_2023_H5N1_2023-04-11
A_Black-headed_gull_Netherlands_40_2023_H5N1_2023-04-19
A_Chroicocephalus_ridibundus_Belgium_01145_0006_2023_H5N1_2023-02-07
A_black-headed_gull_Czech_Republic_6947-4_2023_H5N1_2023-05-03
A_Vulpes_vulpes_Belgium_02359_0006_2023_H5N1_2023-03-14
A_black-headed_gull_Czech_Republic_5152-4_2023_H5N1_2023-04-04
A_Vulpes_vulpes_Belgium_03117_0001_2023_H5N1_2023-03-29
A_arctic-fox_Finland_666_2023_H5N1_2023-07-31_FarmX
A_black-headed_gull_Finland_8135_2023_H5N1_2023-07-193
A_black-headed_gull_Finland_8257_2023_H5N1_2023-07-18
A_black-headed_gull_Finland_8134_23VIR7026-8_2023_H5N1_2023-07-21
A_arctic-fox_Finland_670_2023_H5N1_2023-07-31_FarmX
A_Vulpes_Vulpes_Belgium_04016_0001_2023_H5N1_2023-04-24
A_arctic-fox_Finland_783_2023_H5N1_2023-08-08_FarmB
A_arctic-fox_Finland_491_23_23VIR6803-1_2023_H5N1_2023-07-06_FarmB
A_Pavo_cristatus_Belgium_02559_0001_2023_H5N1_2023-03-17
A_black-headed_gull_Finland_8598_2023_H5N1_2023-07-20
A_black-headed_gull_Finland_8599_2023_H5N1_2023-07-20
A_black-headed_gull_Czech_Republic_5695-2_2023_H5N1_2023-04-13
A_black-headed_gull_Czech_Republic_6947-3_2023_H5N1_2023-05-03
A_black-headed_gull_Czech_Republic_5695-4_2023_H5N1_2023-04-13
A_Chroicocephalus_ridibundus_Belgium_03706_0002_2023_H5N1_2023-04-18
A_Vulpes_vulpes_Belgium_02359_0002_2023_H5N1_2023-03-14
A_Black-headed_gull_Netherlands_8_2023_H5N1_2023-02-20
A_Chroicocephalus_ridibundus_Belgium_01145_0008_2023_H5N1_2023-02-07
A_black-headead_gull_Spain_512-7_23VIR1687-7_2023_H5N1_2023-01-31
A_Falco_peregrinus_Belgium_01823_0003_2023_H5N1_2023-03-01
A_Vulpes_vulpes_Belgium_01924_0003_2023_H5N1_2023-03-02
A_black-headead_gull_Spain_171-1_23VIR1687-20_2023_H5N1_2023-01-09
A_Falco_peregrinus_Belgium_01145_0004_2023_H5N1_2023-02-07
A_Falco_peregrinus_Belgium_01145_0005_2023_H5N1_2023-06-07
A_Black-headed_Gull_Netherlands_73_2023_H5N1_2023-05-22
A_Falco_peregrinus_Belgium_02423_0004_2023_H5N1_2023-03-15
A_black-headed_gull_Czech_Republic_5487-4_2023_H5N1_2023-04-12
A_black-headed_gull_Finland_6726_2023_H5N1_2023-06-13
A_arctic-fox_Finland_648_2023_H5N1_2023-07-28_FarmJ
A_black-headed_gull_Czech_Republic_6633-1_2023_H5N1_2023-04-24
A_fox_France_23P002844_2023_H5N1_2023-02-10
A_raccoon-dog_Finland_552_23VIR6928-5_2023_H5N1_2023-07-17_FarmI
A_raccoon-dog_Finland_555_2023_H5N1_2023-07-17_FarmI
A_fox_Finland_610_2023_H5N1_2023-07-20_FarmQ
A_black-headed_gull_Finland_8264_23VIR7026-19_2023_H5N1_2023-07-21
A_domestic_duck_England_049343_2023_H5N1_2023-03-30
A_guinea_fowl_England_049422_2023_H5N1_2023-03-30
A_Falco_peregrinus_Belgium_03518_0004_2023_H5N1_2023-04-11
A_Falco_peregrinus_Belgium_04695_0002_2023_H5N1_2023-05-07
A_Chroicocephalus_ridibundus_Belgium_03706_0003_2023_H5N1_2023-04-18
A_arctic-fox_Finland_654_2023_H5N1_2023-07-27_FarmV
A_fox_Finland_603_2023_H5N1_2023-07-20_FarmN
A_black-headed_gull_Czech_Republic_6577-3_2023_H5N1_2023-04-24
A_peregrine_falcon_Czech_Republic_7735-2_2023_H5N1_2023-05-17
A_peregrine_falcon_Czech_Republic_7735-2_chymus_2023_H5N1_2023-05-17
A_peregrine_falcon_Czech_Republic_7735-1_2023_H5N1_2023-05-17
A_black-headed_gull_Austria_23030829-001_2023_H5N1_2023-03-08
A_black-headed_gull_Austria_23029679_2023_H5N1_2023-03-07
A_black-headed_gull_Czech_Republic_5295-3_2023_H5N1_2023-04-03
A_black-headed_gull_Austria_23037203-001_2023_H5N1_2023-01-21
A_black-headed_gull_Austria_23061496-001_2023_H5N1_2023-05-09
A_black-headed_gull_Czech_Republic_6577-5_2023_H5N1_2023-04-24
A_black-headed_gull_Czech_Republic_6634-5_2023_H5N1_2023-05-02
A_black-headed_gull_Czech_Republic_7334-5_2023_H5N1_2023-05-11
A_black-headed_gull_Austria_23031105-002_2023_H5N1_2023-03-08
A_Vulpes_Vulpes_Belgium_03703_0001_2023_H5N1_2023-04-14
A_Vulpes_vulpes_Belgium_03117_0004_2023_H5N1_2023-03-29
A_Vulpes_Vulpes_Belgium_03704_0001_2023_H5N1_2023-04-14
A_Black-headed_gull_Netherlands_39_2023_H5N1_2023-04-17
A_Mediterranean_Gull_Netherlands_3_2023_H5N1_2023-05-26
A_black-headed_gull_Finland_8145_2023_H5N1_2023-07-19
A_arctic-fox_Finland_504_23_23VIR6803-7_2023_H5N1_2023-07-10_FarmE
A_silver_fox_Finland_2023AI06838_033_2023_H5N1_2023-08-04_FarmE
A_wild-fox_Finland_1361_2023_H5N1_2023-08-10
A_red_fox_Finland_2023AI06879_074_2023_H5N1_2023-08-04_FarmE
A_blue_fox_Finland_2023AI06876_071_2023_H5N1_2023-08-04_FarmE
A_arctic-fox_Finland_577_2023_H5N1_2023-07-18_FarmL
A_arctic-fox_Finland_578_2023_H5N1_2023-07-18_FarmL
A_arctic-fox_Finland_856_2023_H5N1_2023-08-10_FarmE
A_arctic-fox_Finland_536_2023_H5N1_2023-07-14_FarmF
A_arctic-fox_Finland_559_2023_H5N1_2023-07-17_FarmJ
A_arctic-fox_Finland_618_2023_H5N1_2023-07-24_FarmS
A_arctic-fox_Finland_616_2023_H5N1_2023-07-24_FarmS
A_arctic-fox_Finland_538_2023_H5N1_2023-07-14_FarmF
A_arctic-fox_Finland_615_2023_H5N1_2023-07-24_FarmS
A_arctic-fox_Finland_543_2023_H5N1_2023-07-14_FarmG
A_arctic-fox_Finland_501_2023_H5N1_2023-07-10_FarmE
A_arctic-fox_Finland_1086_2023_H5N1_2023-08-09_FarmG
A_arctic-fox_Finland_966_2023_H5N1_2023-08-10_FarmG
A_arctic-fox_Finland_1305_2023_H5N1_2023-08-10_FarmO
A_black-headed_gull_Finland_8081_23VIR6928-12_2023_H5N1_2023-07-12
A_arctic-fox_Finland_738_2023_H5N1_2023-08-07_FarmE
A_silver_fox_Finland_2023AI06834_029_2023_H5N1_2023-08-04_FarmE
A_black-headed_gull_Finland_8095_2023_H5N1_2023-07-18
A_black-headed_gull_Finland_8096_23VIR7026-6_2023_H5N1_2023-07-20
A_arctic-fox_Finland_1406_2023_H5N1_2023-08-17_FarmE
A_arctic-fox_Finland_579_2023_H5N1_2023-07-18_FarmL
A_arctic-fox_Finland_736_2023_H5N1_2023-08-07_FarmE
A_arctic-fox_Finland_545_2023_H5N1_2023-07-14_FarmG
A_arctic-fox_Finland_564_2023_H5N1_2023-07-17_FarmK
A_black-headed_gull_Finland_8079_23VIR6928-11_2023_H5N1_2023-07-12
A_blue_fox_Finland_2023AI06820_015_2023_H5N1_2023-08-04_FarmE
A_raccoon-dog_Finland_623_2023_H5N1_2023-07-24_FarmT
A_black-headed_gull_Finland_8141_23VIR7026-11_2023_H5N1_2023-07-21
A_arctic-fox_Finland_752_2023_H5N1_2023-08-07_FarmE
A_black-headed_gull_Finland_8078_23VIR6928-10_2023_H5N1_2023-07-12
A_raccoon-dog_Finland_625_2023_H5N1_2023-07-24_FarmT
A_black-headed_gull_Finland_8247_2023_H5N1_2023-07-18
A_arctic-fox_Finland_566_2023_H5N1_2023-07-17_FarmK
A_arctic-fox_Finland_613_2023_H5N1_2023-07-24_FarmR
A_arctic-fox_Finland_497_23_23VIR6803-5_2023_H5N1_2023-07-10_FarmD
A_arctic-fox_Finland_503_23_23VIR6803-6_2023_H5N1_2023-07-10_FarmE
A_arctic-fox_Finland_495_2023_H5N1_2023-07-10_FarmD
A_arctic-fox_Finland_619_2023_H5N1_2023-07-24_FarmT
A_arctic-fox_Finland_546_2023_H5N1_2023-07-14_FarmG
A_arctic-fox_Finland_620_2023_H5N1_2023-07-24_FarmT
A_arctic-fox_Finland_565_2023_H5N1_2023-07-17_FarmK
A_arctic-fox_Finland_567_2023_H5N1_2023-07-17_FarmK
A_arctic-fox_Finland_542_2023_H5N1_2023-07-14_FarmG
A_arctic-fox_Finland_614_2023_H5N1_2023-07-24_FarmR
A_arctic-fox_Finland_574_2023_H5N1_2023-07-18_FarmL
A_arctic-fox_Finland_612_2023_H5N1_2023-07-24_FarmR
A_arctic-fox_Finland_575_2023_H5N1_2023-07-18_FarmL
A_arctic-fox_Finland_547_2023_H5N1_2023-07-14_FarmH
A_arctic-fox_Finland_549_2023_H5N1_2023-07-14_FarmH
A_arctic-fox_Finland_622_2023_H5N1_2023-07-24_FarmT
A_fox_Finland_601_2023_H5N1_2023-07-20_FarmN
A_arctic-fox_Finland_660_2023_H5N1_2023-07-31_FarmW
A_arctic-fox_Finland_544_2023_H5N1_2023-07-14_FarmG
A_arctic-fox_Finland_621_2023_H5N1_2023-07-24_FarmT
A_arctic-fox_Finland_576_2023_H5N1_2023-07-18_FarmL
A_black-headed_gull_Finland_8139_2023_H5N1_2023-07-19
A_mink_Finland_606_2023_H5N1_2023-07-20_FarmO
A_black-headed_gull_Finland_8246_2023_H5N1_2023-07-20
A_arctic-fox_Finland_500_2023_H5N1_2023-07-10_FarmP
A_arctic-fox_Finland_1842_2023_H5N1_2023-08-17_FarmG
A_arctic-fox_Finland_673_2023_H5N1_2023-08-01_FarmY
A_arctic-fox_Finland_678_2023_H5N1_2023-08-01_FarmY
A_arctic-fox_Finland_676_2023_H5N1_2023-08-01_FarmY
A_arctic-fox_Finland_677_2023_H5N1_2023-08-01_FarmY
A_arctic-fox_Finland_679_2023_H5N1_2023-08-01_FarmY
A_arctic-fox_Finland_675_2023_H5N1_2023-08-01_FarmY
A_arctic-fox_Finland_674_2023_H5N1_2023-08-01_FarmY
A_arctic-fox_Finland_548_2023_H5N1_2023-07-14_FarmH
A_black-headed_gull_Finland_8143_2023_H5N1_2023-07-19
A_fox_Finland_602_2023_H5N1_2023-07-20_FarmN
A_arctic-fox_Finland_496_2023_H5N1_2023-07-10_FarmD
A_arctic-fox_Finland_1908_2023Finland_1908_2023_H5N1_2023-08-21_FarmZ
A_arctic-fox_Finland_1909_2023_H5N1_2023-08-21_FarmZ
A_arctic-fox_Finland_1840_2023_H5N1_2023-08-17_FarmG
A_black-headed_gull_Finland_8077_23VIR6928-9_2023_H5N1_2023-07-12
A_Chroicocephalus_ridibundus_Belgium_00374_0005_2023_H5N1_2023-01-12
A_Vulpes_vulpes_Belgium_01295_0002_2023_H5N1_2023-02-10
A_black-headed_gull_Finland_8244_2023_H5N1_2023-07-20
A_Gallus_gallus_Belgium_14828_0003_2022_H5N1_2022-12-27
A_black-headead_gull_Spain_824-9_23VIR1687-24_2023_H5N1_2023-02-09
A_black-headead_gull_Spain_416-11_23VIR1687-6_2023_H5N1_2023-01-16
A_Sandwich_Tern_Netherlands_1_2023_H5N1_2023-05-17
A_black-headed_gull_Finland_LI7728_23_23VIR6803-9_2023_H5N1_2023-07-03
A_canada_goose_Finland_8109_23VIR7026-20_2023_H5N1_2023-07-18
A_great_crested_grebe_Finland_8111_23VIR7026-21_2023_H5N1_2023-07-18
A_Black-headed_Gull_Netherlands_66_2023_H5N1_2023-05-19
A_raxorbill_Finland_9072_2023_H5N1_2023-07-27_Geta
A_Black-headed_Gull_Netherlands_74_2023_H5N1_2023-05-28
A_Black-headed_gull_Netherlands_43_2023_H5N1_2023-04-29
A_Common_Tern_Netherlands_6_2023_H5N1_2023-05-31
A_Black-headed_gull_Netherlands_44_2023_H5N1_2023-04-30
A_Black-headed_Gull_Netherlands_86_2023_H5N1_2023-06-16
A_Common_Tern_Netherlands_3_2023_H5N1_2023-05-26
A_Black-headed_gull_Netherlands_42_2023_H5N1_2023-04-29
A_Black-headed_Gull_Netherlands_87_2023_H5N1_2023-06-16
A_Common_Tern_Netherlands_8_2023_H5N1_2023-06-03
A_Black-headed_Gull_Netherlands_83_2023_H5N1_2023-06-09
A_Black-headed_Gull_Netherlands_23007954-002_2023_H5N1_2023-05-08
A_Black-headed_Gull_Netherlands_23006666-002_2023_H5N1_2023-04-17
A_Chroicocephalus_ridibundus_Belgium_00816_0001_2023_H5N1_2023-01-26
A_Falco_peregrinus_Belgium_02026_0005_2023_H5N1_2023-03-07
A_Common_Tern_Netherlands_1_2023_H5N1_2023-05-19
A_Common_Tern_Netherlands_2_2023_H5N1_2023-05-27
A_Lesser_black-backed_Gull_Netherlands_2_2023_H5N1_2023-05-19
A_Numenius_arquata_Belgium_01456_0003_2023_H5N1_2023-02-16
A_Black-headed_Gull_Netherlands_65_2023_H5N1_2023-05-19
A_Gallus_gallus_Belgium_01434_0001_2023_H5N1_2023-02-15
A_Gallus_gallus_Belgium_01172_0001_2023_H5N1_2023-02-06
A_Chroicocephalus_ridibundus_Belgium_00132_0002_2022_H5N1_2022-12-28
A_black-headed_gull_Finland_7477_2023_H5N1_2023-06-25
A_common_tern_Finland_7479_2023_H5N1_2023-06-25
A_black-headed_gull_Czech_Republic_6577-1_2023_H5N1_2023-04-24
A_Black-headed_Gull_Netherlands_60_2023_H5N1_2023-05-19
A_black-headed_gull_Italy_23VIR1103-4_2023_H5N1_2023-01-31
A_black-headed_gull_Italy_23VIR1205-2_2023_H5N1_2023-02-02
A_black-headed_gull_Italy_23VIR1109-4_2023_H5N1_2023-01-31
A_black-headed_gull_Finland_9079_2023_H5N1_2023-07-28
A_mink_Finland_3081_2023_H5N1_2023-09-20_FarmAA
A_mink_Finland_3080_2023_H5N1_2023-09-20_FarmAA
A_mink_Finland_3079_2023_H5N1_2023-09-20_FarmAA
A_mink_Finland_3076_2023_H5N1_2023-09-20_FarmAA
A_mink_Finland_3077_2023_H5N1_2023-09-20_FarmAA
A_mink_Finland_3078_2023_H5N1_2023-09-20_FarmAA
A_mink_Finland_3082_2023_H5N1_2023-09-20_FarmAA
A_herring_gull_Finland_10812_2023_H5N1_2023-09-18
A_partridge_England_097413_2023_H5N1_2023-07-23
A_Black-headed_Gull_England_259816_2023_H5N1_2023-04-25
A_domestic_duck_England_097245_2023_H5N1_2023-07-23
A_black-headed_gull_England_258811_2023_H5N1_2023-03-25
A_partridge_England_097407_2023_H5N1_2023-07-23
A_herring_gull_Finland_10450_2023_H5N1_2023-08-29
A_Black-headed_gull_Netherlands_22_2023_H5N1_2023-03-04
A_European_herring_gull_Netherlands_4_2023_H5N1_2023-02-20
A_European_herring_gull_Netherlands_7_2023_H5N1_2023-02-20
A_black-legged_kittiwake_Finland_8808_2023_H5N1_2023-07-28
A_red_fox_Norway_2023_80_49_2_2023_H5N1_2023-06-27
A_black-legged_kittiwake_Finland_8772_2023_H5N1_2023-07-23
A_white-tailed_eagle_Finland_10844_2023_H5N1_2023-09-02
A_mute_swan_Denmark_06339-101_2023-08-05_H5N1_2023-08-05
A_Domestic_duck_Wales_020076_2023_H5N1_2023-02-05
A_chicken_Wales_023780_2023_H5N1_2023-02-11
A_white-tailed_eagle_Finland_8154_23VIR7026-22_2023_H5N1_2023-07-18
A_black-headed_gull_Finland_8107_23VIR7026-7_2023_H5N1_2023-07-20
A_white-tailed_eagle_Finland_LI7800_23_23VIR6803-10_2023_H5N1_2023-06-28
A_black-headed_gull_England_119705_2022_H5N1_2022-12-01
A_black-headed_gull_England_119699_2022_H5N1_2022-12-01
A_Chicken_England_155942_2022_H5N1_2022-11-20
A_mute_swan_England_119557_2022_H5N1_2022-11-24
H5N1 Finland 2021
A_chicken_Netherlands_20019879-001005_2020_H5N1
A_eurasian_teal_Netherlands_20016896-013_2020_H5N1
A_Eurasian_Wigeon_Netherlands_5_2020_H5N1
A_greylag_goose_Netherlands_20016582-004_2020_H5N1
A_greater_white-fronted_goose_Italy_20VIR8073-4_2020_H5N1
A_turkey_Italy_21VIR8480-1_2021_H1N1
A_turkey_Italy_21VIR8480-2_2021_H5N1
A_duck_Bangladesh_17D1736_2021_H2N1_2021-08-19
A_environment_Bangladesh_17E82_2021_H6N1_2021-01-10
A_duck_Bangladesh_18D1662_2021_H2N1_2021-06-28
A_duck_Bangladesh_18D1571_2021_H6N1_2021-01-04
A_mallard_Italy_21VIR9957-6_2021_H5N1
0.004

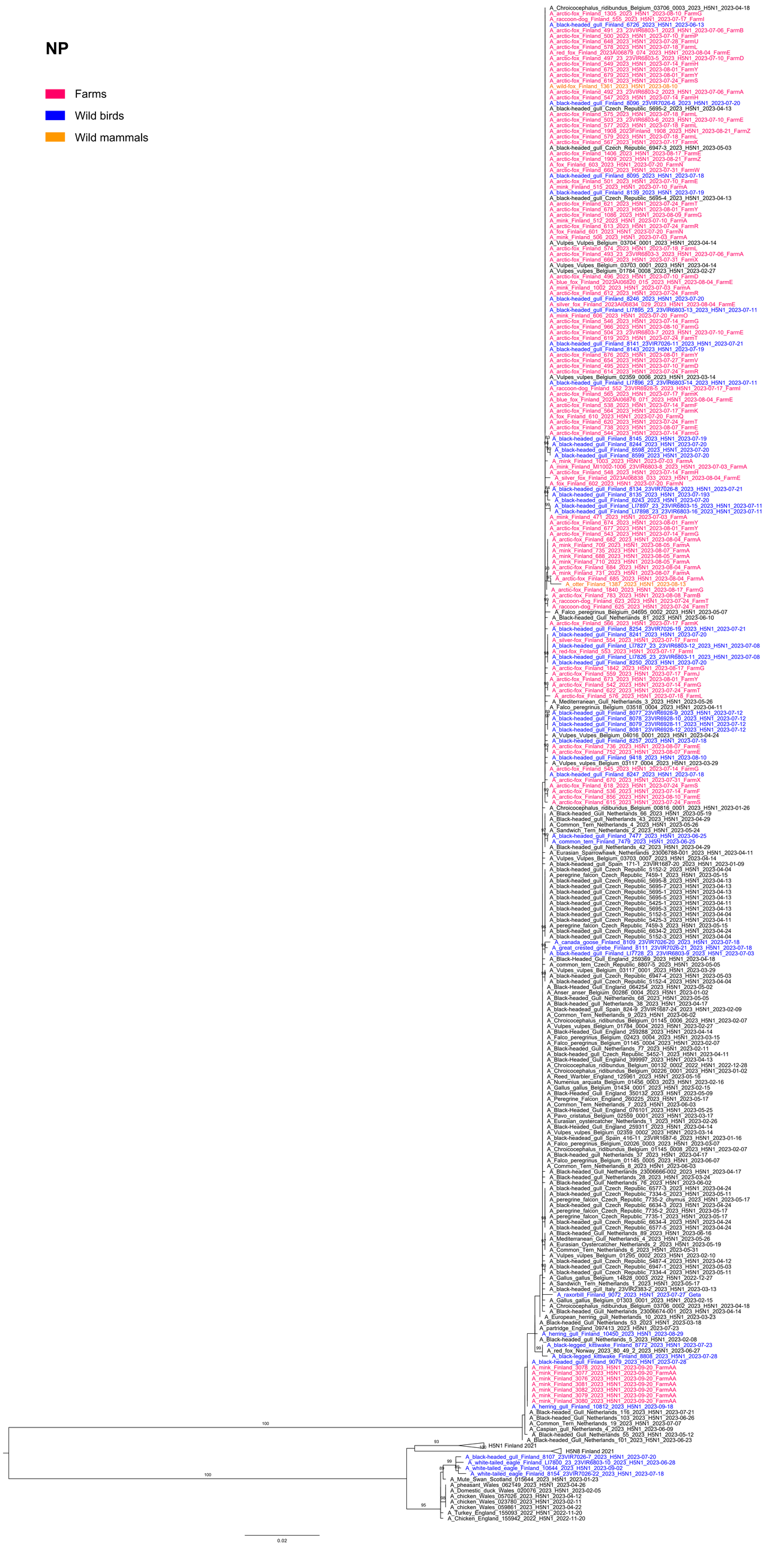
NP
Farms
Wild birds
Wild mammals
A_Chroicocephalus_ridibundus_Belgium_03706_0003_2023_H5N1_2023-04-18
A_arctic-fox_Finland_1305_2023_H5N1_2023-08-10_FarmG
A_raccoon-dog_Finland_555_2023_H5N1_2023-07-17_FarmI
A_black-headed_gull_Finland_6726_2023_H5N1_2023-06-13
A_arctic-fox_Finland_491_23_23VIR6803-1_2023_H5N1_2023-07-06_FarmB
A_arctic-fox_Finland_500_2023_H5N1_2023-07-10_FarmP
A_arctic-fox_Finland_648_2023_H5N1_2023-07-28_FarmU
A_arctic-fox_Finland_578_2023_H5N1_2023-07-18_FarmL
A_red_fox_Finland_2023AI06879_074_2023_H5N1_2023-08-04_FarmE
A_arctic-fox_Finland_497_23_23VIR6803-5_2023_H5N1_2023-07-10_FarmD
A_arctic-fox_Finland_549_2023_H5N1_2023-07-14_FarmH
A_arctic-fox_Finland_675_2023_H5N1_2023-08-01_FarmY
A_arctic-fox_Finland_679_2023_H5N1_2023-08-01_FarmY
A_arctic-fox_Finland_616_2023_H5N1_2023-07-24_FarmS
A_wild-fox_Finland_1361_2023_H5N1_2023-08-10
A_arctic-fox_Finland_492_23_23VIR6803-2_2023_H5N1_2023-07-06_FarmA
A_arctic-fox_Finland_547_2023_H5N1_2023-07-14_FarmH
A_black-headed_gull_Finland_8096_23VIR7026-6_2023_H5N1_2023-07-20
A_black-headed_gull_Czech_Republic_5695-2_2023_H5N1_2023-04-13
A_arctic-fox_Finland_575_2023_H5N1_2023-07-18_FarmL
A_arctic-fox_Finland_503_23_23VIR6803-6_2023_H5N1_2023-07-10_FarmE
A_arctic-fox_Finland_577_2023_H5N1_2023-07-18_FarmL
A_arctic-fox_Finland_1908_2023Finland_1908_2023_H5N1_2023-08-21_FarmZ
A_arctic-fox_Finland_579_2023_H5N1_2023-07-18_FarmL
A_arctic-fox_Finland_587_2023_H5N1_2023-07-17_FarmK
A_black-headed_gull_Czech_Republic_6947-3_2023_H5N1_2023-05-03
A_arctic-fox_Finland_1406_2023_H5N1_2023-08-17_FarmE
A_arctic-fox_Finland_1909_2023_H5N1_2023-08-21_FarmZ
A_fox_Finland_603_2023_H5N1_2023-07-20_FarmN
A_arctic-fox_Finland_660_2023_H5N1_2023-07-31_FarmW
A_black-headed_gull_Finland_8095_2023_H5N1_2023-07-18
A_arctic-fox_Finland_501_2023_H5N1_2023-07-10_FarmE
A_mink_Finland_515_2023_H5N1_2023-07-10_FarmA
A_black-headed_gull_Finland_8139_2023_H5N1_2023-07-19
A_black-headed_gull_Czech_Republic_5695-4_2023_H5N1_2023-04-13
A_arctic-fox_Finland_821_2023_H5N1_2023-07-24_FarmT
A_arctic-fox_Finland_678_2023_H5N1_2023-08-01_FarmY
A_arctic-fox_Finland_1086_2023_H5N1_2023-08-09_FarmG
A_mink_Finland_512_2023_H5N1_2023-07-10_FarmA
A_arctic-fox_Finland_613_2023_H5N1_2023-07-24_FarmR
A_fox_Finland_601_2023_H5N1_2023-07-20_FarmN
A_mink_Finland_506_2023_H5N1_2023-07-03_FarmA
A_Vulpes_Vulpes_Belgium_03704_0001_2023_H5N1_2023-04-14
A_arctic-fox_Finland_574_2023_H5N1_2023-07-18_FarmL
A_arctic-fox_Finland_493_23_23VIR6803-3_2023_H5N1_2023-07-06_FarmA
A_arctic-fox_Finland_666_2023_H5N1_2023-07-31_FarmX
A_Vulpes_Vulpes_Belgium_03703_0001_2023_H5N1_2023-04-14
A_Vulpes_vulpes_Belgium_01784_0008_2023_H5N1_2023-02-27
A_arctic-fox_Finland_496_2023_H5N1_2023-07-10_FarmD
A_blue_fox_Finland_2023AI06820_015_2023_H5N1_2023-08-04_FarmE
A_mink_Finland_1002_2023_H5N1_2023-07-03_FarmA
A_arctic-fox_Finland_612_2023_H5N1_2023-07-24_FarmR
A_black-headed_gull_Finland_8246_2023_H5N1_2023-07-20
A_silver_fox_Finland_2023AI06834_029_2023_H5N1_2023-08-04_FarmE
A_black-headed_gull_Finland_LI7895_23_23VIR6803-13_2023_H5N1_2023-07-11
A_mink_Finland_606_2023_H5N1_2023-07-20_FarmO
A_arctic-fox_Finland_546_2023_H5N1_2023-07-14_FarmG
A_arctic-fox_Finland_966_2023_H5N1_2023-08-10_FarmG
A_arctic-fox_Finland_504_23_23VIR6803-7_2023_H5N1_2023-07-10_FarmE
A_arctic-fox_Finland_619_2023_H5N1_2023-07-24_FarmT
A_black-headed_gull_Finland_8141_23VIR7026-11_2023_H5N1_2023-07-21
A_black-headed_gull_Finland_8143_2023_H5N1_2023-07-19
A_arctic-fox_Finland_676_2023_H5N1_2023-08-01_FarmY
A_arctic-fox_Finland_654_2023_H5N1_2023-07-27_FarmY
A_arctic-fox_Finland_495_2023_H5N1_2023-07-10_FarmD
A_arctic-fox_Finland_614_2023_H5N1_2023-07-24_FarmR
A_Vulpes_vulpes_Belgium_02359_0006_2023_H5N1_2023-03-14
A_black-headed_gull_Finland_LI7896_23_23VIR6803-14_2023_H5N1_2023-07-11
A_raccoon-dog_Finland_552_23VIR6926-5_2023_H5N1_2023-07-17_FarmI
A_arctic-fox_Finland_565_2023_H5N1_2023-07-17_FarmK
A_blue_fox_Finland_2023AI06876_071_2023_H5N1_2023-08-04_FarmE
A_arctic-fox_Finland_538_2023_H5N1_2023-07-14_FarmF
A_arctic-fox_Finland_564_2023_H5N1_2023-07-17_FarmK
A_fox_Finland_610_2023_H5N1_2023-07-20_FarmQ
A_arctic-fox_Finland_620_2023_H5N1_2023-07-24_FarmT
A_arctic-fox_Finland_738_2023_H5N1_2023-08-07_FarmE
A_arctic-fox_Finland_544_2023_H5N1_2023-07-14_FarmG
A_black-headed_gull_Finland_8145_2023_H5N1_2023-07-19
A_black-headed_gull_Finland_8244_2023_H5N1_2023-07-20
A_black-headed_gull_Finland_8598_2023_H5N1_2023-07-20
A_black-headed_gull_Finland_8599_2023_H5N1_2023-07-20
A_mink_Finland_1003_2023_H5N1_2023-07-03_FarmA
A_mink_Finland_MI1002-1006_23VIR6803-8_2023_H5N1_2023-07-03_FarmA
A_arctic-fox_Finland_548_2023_H5N1_2023-07-14_FarmH
A_silver_fox_Finland_2023AI06836_033_2023_H5N1_2023-08-04_FarmE
A_fox_Finland_602_2023_H5N1_2023-07-20_FarmN
A_black-headed_gull_Finland_8134_23VIR7026-8_2023_H5N1_2023-07-21
A_black-headed_gull_Finland_8135_2023_H5N1_2023-07-193
A_black-headed_gull_Finland_8243_2023_H5N1_2023-07-20
A_black-headed_gull_Finland_LI7897_23_23VIR6803-15_2023_H5N1_2023-07-11
A_black-headed_gull_Finland_LI7898_23_23VIR6803-16_2023_H5N1_2023-07-11
A_mink_Finland_471_2023_H5N1_2023-07-03_FarmA
A_arctic-fox_Finland_674_2023_H5N1_2023-08-01_FarmY
A_arctic-fox_Finland_677_2023_H5N1_2023-08-01_FarmY
A_arctic-fox_Finland_543_2023_H5N1_2023-07-14_FarmG
A_arctic-fox_Finland_682_2023_H5N1_2023-08-04_FarmA
A_mink_Finland_709_2023_H5N1_2023-08-05_FarmA
A_mink_Finland_735_2023_H5N1_2023-08-07_FarmA
A_mink_Finland_688_2023_H5N1_2023-08-05_FarmA
A_mink_Finland_710_2023_H5N1_2023-08-05_FarmA
A_arctic-fox_Finland_684_2023_H5N1_2023-08-04_FarmA
A_mink_Finland_731_2023_H5N1_2023-08-07_FarmA
A_arctic-fox_Finland_685_2023_H5N1_2023-08-04_FarmA
A_otter_Finland_1387_2023_H5N1_2023-08-13
A_arctic-fox_Finland_1840_2023_H5N1_2023-08-17_FarmG
A_arctic-fox_Finland_783_2023_H5N1_2023-08-08_FarmB
A_raccoon-dog_Finland_623_2023_H5N1_2023-07-24_FarmT
A_raccoon-dog_Finland_625_2023_H5N1_2023-07-24_FarmT
A_Falco_peregrinus_Belgium_04695_0002_2023_H5N1_2023-05-07
A_Black-headed_Gull_Netherlands_81_2023_H5N1_2023-06-10
A_arctic-fox_Finland_566_2023_H5N1_2023-07-17_FarmK
A_black-headed_gull_Finland_8254_23VIR7026-19_2023_H5N1_2023-07-21
A_black-headed_gull_Finland_8241_2023_H5N1_2023-07-20
A_silver-fox_Finland_554_2023_H5N1_2023-07-17_FarmI
A_black-headed_gull_Finland_LI7827_23_23VIR6803-12_2023_H5N1_2023-07-08
A_red-fox_Finland_553_2023_H5N1_2023-07-17_FarmI
A_black-headed_gull_Finland_LI7826_23_23VIR6803-11_2023_H5N1_2023-07-08
A_black-headed_gull_Finland_8250_2023_H5N1_2023-07-20
A_arctic-fox_Finland_1842_2023_H5N1_2023-08-17_FarmG
A_arctic-fox_Finland_559_2023_H5N1_2023-07-17_FarmJ
A_arctic-fox_Finland_573_2023_H5N1_2023-08-01_FarmY
A_arctic-fox_Finland_542_2023_H5N1_2023-07-14_FarmG
A_arctic-fox_Finland_622_2023_H5N1_2023-07-24_FarmT
A_arctic-fox_Finland_576_2023_H5N1_2023-07-18_FarmL
A_Mediterranean_Gull_Netherlands_3_2023_H5N1_2023-05-26
A_Falco_peregrinus_Belgium_03518_0004_2023_H5N1_2023-04-11
A_black-headed_gull_Finland_8077_23VIR6928-9_2023_H5N1_2023-07-12
A_black-headed_gull_Finland_8078_23VIR6928-10_2023_H5N1_2023-07-12
A_black-headed_gull_Finland_8079_23VIR6928-11_2023_H5N1_2023-07-12
A_black-headed_gull_Finland_8081_23VIR6928-12_2023_H5N1_2023-07-12
A_Vulpes_Vulpes_Belgium_04016_0001_2023_H5N1_2023-04-24
A_black-headed_gull_Finland_8257_2023_H5N1_2023-07-18
A_arctic-fox_Finland_736_2023_H5N1_2023-08-07_FarmE
A_arctic-fox_Finland_752_2023_H5N1_2023-08-07_FarmE
A_black-headed_gull_Finland_9418_2023_H5N1_2023-08-10
A_Vulpes_vulpes_Belgium_03117_0004_2023_H5N1_2023-03-29
A_arctic-fox_Finland_545_2023_H5N1_2023-07-14_FarmG
A_black-headed_gull_Finland_8247_2023_H5N1_2023-07-18
A_arctic-fox_Finland_670_2023_H5N1_2023-07-31_FarmX
A_arctic-fox_Finland_618_2023_H5N1_2023-07-24_FarmS
A_arctic-fox_Finland_536_2023_H5N1_2023-07-14_FarmF
A_arctic-fox_Finland_856_2023_H5N1_2023-08-10_FarmE
A_arctic-fox_Finland_615_2023_H5N1_2023-07-24_FarmS
A_Chroicocephalus_ridibundus_Belgium_00816_0001_2023_H5N1_2023-01-26
A_Black-headed_Gull_Netherlands_66_2023_H5N1_2023-05-19
A_Black-headed_gull_Netherlands_43_2023_H5N1_2023-04-29
A_Common_Tern_Netherlands_4_2023_H5N1_2023-05-26
A_Sandwich_Tern_Netherlands_2_2023_H5N1_2023-05-24
A_black-headed_gull_Finland_7477_2023_H5N1_2023-06-25
A_common_tern_Finland_7479_2023_H5N1_2023-06-25
A_Black-headed_gull_Netherlands_42_2023_H5N1_2023-04-29
A_Eurasian_Sparrowhawk_Netherlands_23006788-001_2023_H5N1_2023-04-11
A_Vulpes_Vulpes_Belgium_03703_0007_2023_H5N1_2023-04-14
A_black-headed_gull_Spain_171-1_23VIR1687-20_2023_H5N1_2023-01-09
A_black-headed_gull_Czech_Republic_5152-2_2023_H5N1_2023-04-04
A_peregrine_falcon_Czech_Republic_7459-1_2023_H5N1_2023-05-15
A_black-headed_gull_Czech_Republic_5695-8_2023_H5N1_2023-04-13
A_black-headed_gull_Czech_Republic_5695-7_2023_H5N1_2023-04-13
A_black-headed_gull_Czech_Republic_5695-1_2023_H5N1_2023-04-13
A_black-headed_gull_Czech_Republic_5695-5_2023_H5N1_2023-04-13
A_black-headed_gull_Czech_Republic_5425-1_2023_H5N1_2023-04-11
A_black-headed_gull_Czech_Republic_5695-3_2023_H5N1_2023-04-13
A_black-headed_gull_Czech_Republic_5152-5_2023_H5N1_2023-04-04
A_black-headed_gull_Czech_Republic_5425-3_2023_H5N1_2023-04-11
A_peregrine_falcon_Czech_Republic_7459-3_2023_H5N1_2023-05-15
A_black-headed_gull_Czech_Republic_6634-2_2023_H5N1_2023-04-24
A_black-headed_gull_Czech_Republic_5152-3_2023_H5N1_2023-04-04
A_canada_goose_Finland_8109_23VIR7026-20_2023_H5N1_2023-07-18
A_great_crested_grebe_Finland_8111_23VIR7026-21_2023_H5N1_2023-07-18
A_black-headed_gull_Finland_LI7728_23_23VIR6803-9_2023_H5N1_2023-07-03
A_Black-Headed_Gull_England_259369_2023_H5N1_2023-04-18
A_common_tern_Czech_Republic_8807-5_2023_H5N1_2023-05-05
A_Vulpes_vulpes_Belgium_03117_0001_2023_H5N1_2023-03-29
A_black-headed_gull_Czech_Republic_6947-4_2023_H5N1_2023-05-03
A_black-headed_gull_Czech_Republic_5152-4_2023_H5N1_2023-04-04
A_Black-Headed_Gull_England_064254_2023_H5N1_2023-05-02
A_Anser_anser_Belgium_00286_0004_2023_H5N1_2023-01-02
A_Black-headed_Gull_Netherlands_68_2023_H5N1_2023-05-05
A_Black-headed_gull_Netherlands_38_2023_H5N1_2023-04-17
A_black-headead_gull_Spain_824-9_23VIR1687-24_2023_H5N1_2023-02-09
A_Common_Tern_Netherlands_9_2023_H5N1_2023-06-02
A_Chroicocephalus_ridibundus_Belgium_01145_0006_2023_H5N1_2023-02-07
A_Vulpes_vulpes_Belgium_01784_0004_2023_H5N1_2023-02-27
A_Black-Headed_Gull_England_259288_2023_H5N1_2023-04-14
A_Falco_peregrinus_Belgium_02423_0004_2023_H5N1_2023-03-15
A_Falco_peregrinus_Belgium_01145_0004_2023_H5N1_2023-02-07
A_Black-headed_Gull_Netherlands_77_2023_H5N1_2023-02-11
A_black-headed_gull_Czech_Republic_5452-1_2023_H5N1_2023-04-11
A_Black-Headed_Gull_England_399997_2023_H5N1_2023-04-13
A_Chroicocephalus_ridibundus_Belgium_00132_0002_2022_H5N1_2022-12-28
A_Chroicocephalus_ridibundus_Belgium_00226_0001_2023_H5N1_2023-01-02
A_Reed_Warbler_England_125961_2023_H5N1_2023-05-16
A_Numenius_arquata_Belgium_01456_0003_2023_H5N1_2023-02-16
A_Gallus_gallus_Belgium_01434_0001_2023_H5N1_2023-02-15
A_Black-Headed_Gull_England_350132_2023_H5N1_2023-05-09
A_Peregrine_Falcon_England_260225_2023_H5N1_2023-05-17
A_Common_Tern_Netherlands_7_2023_H5N1_2023-06-03
A_Black-Headed_Gull_England_076101_2023_H5N1_2023-05-25
A_Pavo_cristatus_Belgium_02559_0001_2023_H5N1_2023-03-17
A_Eurasian_oystercatcher_Netherlands_1_2023_H5N1_2023-02-26
A_Black-Headed_Gull_England_259311_2023_H5N1_2023-04-14
A_Vulpes_vulpes_Belgium_02359_0002_2023_H5N1_2023-03-14
A_black-headead_gull_Spain_416-11_23VIR1687-6_2023_H5N1_2023-01-16
A_Falco_peregrinus_Belgium_02026_0003_2023_H5N1_2023-03-07
A_Chroicocephalus_ridibundus_Belgium_01145_0008_2023_H5N1_2023-02-07
A_Black-headed_gull_Netherlands_37_2023_H5N1_2023-04-17
A_Falco_peregrinus_Belgium_01145_0005_2023_H5N1_2023-06-07
A_Common_Tern_Netherlands_8_2023_H5N1_2023-06-03
A_Black-headed_Gull_Netherlands_23006666-002_2023_H5N1_2023-04-17
A_Black-headed_gull_Netherlands_28_2023_H5N1_2023-03-24
A_Black-headed_Gull_Netherlands_76_2023_H5N1_2023-06-02
A_black-headed_gull_Czech_Republic_6577-3_2023_H5N1_2023-04-24
A_black-headed_gull_Czech_Republic_7334-5_2023_H5N1_2023-05-11
A_peregrine_falcon_Czech_Republic_7735-2_chymus_2023_H5N1_2023-05-17
A_black-headed_gull_Czech_Republic_6634-3_2023_H5N1_2023-04-24
A_peregrine_falcon_Czech_Republic_7735-2_2023_H5N1_2023-05-17
A_peregrine_falcon_Czech_Republic_7735-1_2023_H5N1_2023-05-17
A_black-headed_gull_Czech_Republic_6634-4_2023_H5N1_2023-04-24
A_black-headed_gull_Czech_Republic_6577-5_2023_H5N1_2023-04-24
A_Black-headed_Gull_Netherlands_89_2023_H5N1_2023-06-16
A_Mediterranean_Gull_Netherlands_4_2023_H5N1_2023-05-26
A_Eurasian_Oystercatcher_Netherlands_2_2023_H5N1_2023-05-19
A_Common_Tern_Netherlands_6_2023_H5N1_2023-05-31
A_Vulpes_vulpes_Belgium_01295_0002_2023_H5N1_2023-02-10
A_black-headed_gull_Czech_Republic_5487-4_2023_H5N1_2023-04-12
A_black-headed_gull_Czech_Republic_6947-1_2023_H5N1_2023-05-03
A_black-headed_gull_Czech_Republic_7334-4_2023_H5N1_2023-05-11
A_Gallus_gallus_Belgium_14828_0005_2022_H5N1_2022-12-27
A_Sandwich_Tern_Netherlands_1_2023_H5N1_2023-05-17
A_black-headed_gull_Italy_23VIR2383-2_2023_H5N1_2023-03-13
A_raxorbill_Finland_9072_2023_H5N1_2023-07-07_Geta
A_Gallus_gallus_Belgium_01303_0001_2023_H5N1_2023-02-15
A_Chroicocephalus_ridibundus_Belgium_03706_0002_2023_H5N1_2023-04-18
A_Black-headed_Gull_Netherlands_23006674-001_2023_H5N1_2023-04-14
A_Eurpean_herring_gull_Netherlands_10_2023_H5N1_2023-03-23
A_Black-headed_Gull_Netherlands_53_2023_H5N1_2023-03-18
A_partridge_England_097413_2023_H5N1_2023-07-23
A_herring_gull_Finland_10450_2023_H5N1_2023-08-29
A_Black-headed_gull_Netherlands_5_2023_H5N1_2023-02-08
A_black-legged_kittiwake_Finland_8772_2023_H5N1_2023-07-23
A_red_fox_Norway_2023_80_49_2_2023_H5N1_2023-06-27
A_black-legged_kittiwake_Finland_8808_2023_H5N1_2023-07-28
A_black-headed_gull_Finland_9079_2023_H5N1_2023-07-26
A_mink_Finland_3078_2023_H5N1_2023-09-20_FarmAA
A_mink_Finland_3077_2023_H5N1_2023-09-20_FarmAA
A_mink_Finland_3076_2023_H5N1_2023-09-20_FarmAA
A_mink_Finland_3081_2023_H5N1_2023-09-20_FarmAA
A_mink_Finland_3082_2023_H5N1_2023-09-20_FarmAA
A_mink_Finland_3079_2023_H5N1_2023-09-20_FarmAA
A_mink_Finland_3080_2023_H5N1_2023-09-20_FarmAA
A_herring_gull_Finland_10812_2023_H5N1_2023-09-18
A_Black-headed_Gull_Netherlands_116_2023_H5N1_2023-07-21
A_Black-headed_Gull_Netherlands_103_2023_H5N1_2023-06-26
A_Common_Tern_Netherlands_19_2023_H5N1_2023-07-07
A_Caspian_gull_Netherlands_4_2023_H5N1_2023-06-09
A_Black-headed_Gull_Netherlands_55_2023_H5N1_2023-05-12
A_Black-headed_Gull_Netherlands_101_2023_H5N1_2023-06-23
H5N1 Finland 2021
H5N8 Finland 2021
A_black-headed_gull_Finland_8107_23VIR7026-7_2023_H5N1_2023-07-20
A_white-tailed_eagle_Finland_LI7800_23_23VIR6803-10_2023_H5N1_2023-06-28
A_white-tailed_eagle_Finland_10644_2023_H5N1_2023-09-02
A_white-tailed_eagle_Finland_8154_23VIR7026-22_2023_H5N1_2023-07-18
A_Mute_Swan_Scotland_015644_2023_H5N1_2023-01-23
A_pheasant_Wales_062T49_2023_H5N1_2023-04-26
A_Domestic_duck_Wales_020076_2023_H5N1_2023-02-05
A_chicken_Wales_057026_2023_H5N1_2023-04-12
A_chicken_Wales_023780_2023_H5N1_2023-02-11
A_chicken_Wales_059861_2023_H5N1_2023-04-22
A_Turkey_England_155093_2022_H5N1_2022-11-20
A_Chicken_England_155942_2022_H5N1_2022-11-20
100
93
100
99
99
89
98
95
0.02

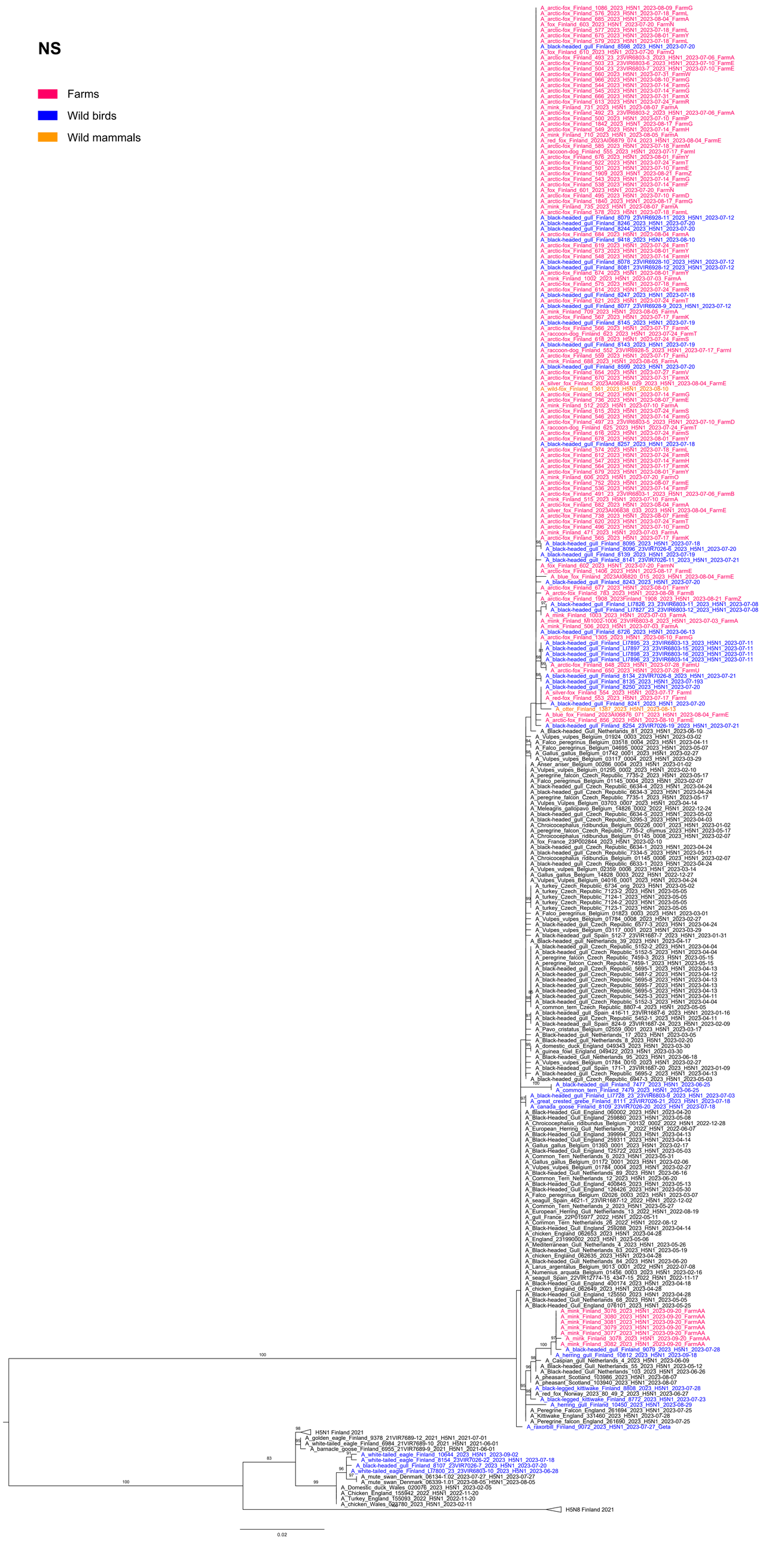
NS
Farms
Wild birds
Wild mammals
A_arctic-fox_Finland_1086_2023_H5N1_2023-08-09_FarmG
A_arctic-fox_Finland_576_2023_H5N1_2023-07-18_FarmL
A_arctic-fox_Finland_685_2023_H5N1_2023-08-04_FarmA
A_fox_Finland_603_2023_H5N1_2023-07-29_FarmN
A_arctic-fox_Finland_577_2023_H5N1_2023-07-18_FarmL
A_arctic-fox_Finland_675_2023_H5N1_2023-08-01_FarmY
A_arctic-fox_Finland_579_2023_H5N1_2023-07-18_FarmL
A_black-headed_gull_Finland_8598_2023_H5N1_2023-07-20
A_fox_Finland_610_2023_H5N1_2023-07-20_FarmQ
A_arctic-fox_Finland_493_23_23VIR6803-3_2023_H5N1_2023-07-06_FarmA
A_arctic-fox_Finland_503_23_23VIR6803-6_2023_H5N1_2023-07-10_FarmE
A_arctic-fox_Finland_504_23_23VIR6803-7_2023_H5N1_2023-07-10_FarmE
A_arctic-fox_Finland_660_2023_H5N1_2023-07-31_FarmW
A_arctic-fox_Finland_966_2023_H5N1_2023-08-10_FarmG
A_arctic-fox_Finland_544_2023_H5N1_2023-07-14_FarmG
A_arctic-fox_Finland_545_2023_H5N1_2023-07-14_FarmG
A_arctic-fox_Finland_666_2023_H5N1_2023-07-31_FarmX
A_arctic-fox_Finland_613_2023_H5N1_2023-07-24_FarmR
A_mink_Finland_731_2023_H5N1_2023-08-07_FarmA
A_arctic-fox_Finland_492_23_23VIR6803-2_2023_H5N1_2023-07-06_FarmA
A_arctic-fox_Finland_500_2023_H5N1_2023-07-10_FarmP
A_arctic-fox_Finland_1842_2023_H5N1_2023-08-17_FarmG
A_arctic-fox_Finland_549_2023_H5N1_2023-07-14_FarmH
A_mink_Finland_710_2023_H5N1_2023-08-05_FarmA
A_red_fox_Finland_2023AI06879_074_2023_H5N1_2023-08-04_FarmE
A_arctic-fox_Finland_585_2023_H5N1_2023-07-18_FarmM
A_raccoon-dog_Finland_555_2023_H5N1_2023-07-17_FarmI
A_arctic-fox_Finland_676_2023_H5N1_2023-08-01_FarmY
A_arctic-fox_Finland_622_2023_H5N1_2023-07-24_FarmT
A_arctic-fox_Finland_501_2023_H5N1_2023-07-10_FarmE
A_arctic-fox_Finland_1909_2023_H5N1_2023-08-21_FarmZ
A_arctic-fox_Finland_543_2023_H5N1_2023-07-14_FarmG
A_arctic-fox_Finland_538_2023_H5N1_2023-07-14_FarmF
A_fox_Finland_601_2023_H5N1_2023-07-20_FarmN
A_arctic-fox_Finland_495_2023_H5N1_2023-07-10_FarmD
A_arctic-fox_Finland_1840_2023_H5N1_2023-08-17_FarmG
A_mink_Finland_735_2023_H5N1_2023-08-07_FarmA
A_arctic-fox_Finland_578_2023_H5N1_2023-07-18_FarmL
A_black-headed_gull_Finland_8079_23VIR6928-11_2023_H5N1_2023-07-12
A_black-headed_gull_Finland_8246_2023_H5N1_2023-07-20
A_black-headed_gull_Finland_8244_2023_H5N1_2023-07-20
A_arctic-fox_Finland_894_2023_H5N1_2023-08-04_FarmA
A_black-headed_gull_Finland_9418_2023_H5N1_2023-08-10
A_arctic-fox_Finland_619_2023_H5N1_2023-07-24_FarmT
A_arctic-fox_Finland_673_2023_H5N1_2023-08-01_FarmY
A_arctic-fox_Finland_548_2023_H5N1_2023-07-14_FarmH
A_black-headed_gull_Finland_8078_23VIR6928-10_2023_H5N1_2023-07-12
A_black-headed_gull_Finland_8081_23VIR6928-12_2023_H5N1_2023-07-12
A_arctic-fox_Finland_674_2023_H5N1_2023-08-01_FarmY
A_mink_Finland_1002_2023_H5N1_2023-07-03_FarmA
A_arctic-fox_Finland_575_2023_H5N1_2023-07-18_FarmL
A_arctic-fox_Finland_614_2023_H5N1_2023-07-24_FarmR
A_black-headed_gull_Finland_8247_2023_H5N1_2023-07-18
A_arctic-fox_Finland_621_2023_H5N1_2023-07-24_FarmT
A_black-headed_gull_Finland_8077_23VIR6928-9_2023_H5N1_2023-07-12
A_mink_Finland_709_2023_H5N1_2023-08-05_FarmA
A_arctic-fox_Finland_567_2023_H5N1_2023-07-17_FarmK
A_black-headed_gull_Finland_8145_2023_H5N1_2023-07-19
A_arctic-fox_Finland_566_2023_H5N1_2023-07-17_FarmK
A_raccoon-dog_Finland_623_2023_H5N1_2023-07-24_FarmT
A_arctic-fox_Finland_618_2023_H5N1_2023-07-24_FarmS
A_black-headed_gull_Finland_8143_2023_H5N1_2023-07-19
A_raccoon-dog_Finland_552_23VIR6928-5_2023_H5N1_2023-07-17_FarmI
A_arctic-fox_Finland_559_2023_H5N1_2023-07-17_FarmJ
A_mink_Finland_688_2023_H5N1_2023-08-05_FarmA
A_black-headed_gull_Finland_8599_2023_H5N1_2023-07-20
A_arctic-fox_Finland_654_2023_H5N1_2023-07-27_FarmV
A_arctic-fox_Finland_670_2023_H5N1_2023-07-31_FarmX
A_silver_fox_Finland_2023AI06834_029_2023_H5N1_2023-08-04_FarmE
A_wild-fox_Finland_1361_2023_H5N1_2023-08-10
A_arctic-fox_Finland_542_2023_H5N1_2023-07-14_FarmG
A_arctic-fox_Finland_736_2023_H5N1_2023-08-07_FarmE
A_mink_Finland_512_2023_H5N1_2023-07-10_FarmA
A_arctic-fox_Finland_615_2023_H5N1_2023-07-24_FarmS
A_arctic-fox_Finland_546_2023_H5N1_2023-07-14_FarmG
A_arctic-fox_Finland_497_23_23VIR6803-5_2023_H5N1_2023-07-10_FarmD
A_raccoon-dog_Finland_625_2023_H5N1_2023-07-24_FarmT
A_arctic-fox_Finland_616_2023_H5N1_2023-07-24_FarmS
A_arctic-fox_Finland_678_2023_H5N1_2023-08-01_FarmY
A_black-headed_gull_Finland_8257_2023_H5N1_2023-07-18
A_arctic-fox_Finland_574_2023_H5N1_2023-07-18_FarmL
A_arctic-fox_Finland_612_2023_H5N1_2023-07-24_FarmR
A_arctic-fox_Finland_547_2023_H5N1_2023-07-14_FarmH
A_arctic-fox_Finland_564_2023_H5N1_2023-07-17_FarmK
A_arctic-fox_Finland_679_2023_H5N1_2023-08-01_FarmY
A_mink_Finland_606_2023_H5N1_2023-07-20_FarmQ
A_arctic-fox_Finland_752_2023_H5N1_2023-08-07_FarmE
A_arctic-fox_Finland_536_2023_H5N1_2023-07-14_FarmF
A_arctic-fox_Finland_491_23_23VIR6803-1_2023_H5N1_2023-07-06_FarmB
A_mink_Finland_515_2023_H5N1_2023-07-10_FarmA
A_arctic-fox_Finland_682_2023_H5N1_2023-08-04_FarmA
A_silver_fox_Finland_2023AI06838_033_2023_H5N1_2023-08-04_FarmE
A_arctic-fox_Finland_738_2023_H5N1_2023-08-07_FarmE
A_arctic-fox_Finland_620_2023_H5N1_2023-07-24_FarmT
A_arctic-fox_Finland_496_2023_H5N1_2023-07-10_FarmD
A_mink_Finland_471_2023_H5N1_2023-07-03_FarmA
A_arctic-fox_Finland_565_2023_H5N1_2023-07-17_FarmK
A_black-headed_gull_Finland_8095_2023_H5N1_2023-07-18
A_black-headed_gull_Finland_8096_23VIR7026-8_2023_H5N1_2023-07-20
A_black-headed_gull_Finland_8139_2023_H5N1_2023-07-19
A_black-headed_gull_Finland_8141_23VIR7026-11_2023_H5N1_2023-07-21
A_fox_Finland_602_2023_H5N1_2023-07-20_FarmN
A_arctic-fox_Finland_1406_2023_H5N1_2023-08-17_FarmE
A_blue_fox_Finland_2023AI06820_015_2023_H5N1_2023-08-04_FarmE
A_black-headed_gull_Finland_8243_2023_H5N1_2023-07-20
A_arctic-fox_Finland_677_2023_H5N1_2023-08-01_FarmY
A_arctic-fox_Finland_783_2023_H5N1_2023-08-08_FarmB
A_arctic-fox_Finland_1908_2023Finland_1908_2023_H5N1_2023-08-21_FarmZ
A_black-headed_gull_Finland_LI7826_23_23VIR6803-11_2023_H5N1_2023-07-08
A_black-headed_gull_Finland_LI7827_23_23VIR6803-12_2023_H5N1_2023-07-08
A_mink_Finland_1003_2023_H5N1_2023-07-03_FarmA
A_mink_Finland_MI1002-1006_23VIR6803-8_2023_H5N1_2023-07-03_FarmA
A_mink_Finland_506_2023_H5N1_2023-07-03_FarmA
A_black-headed_gull_Finland_6726_2023_H5N1_2023-06-13
A_arctic-fox_Finland_1305_2023_H5N1_2023-08-10_FarmG
A_black-headed_gull_Finland_LI7895_23_23VIR6803-13_2023_H5N1_2023-07-11
A_black-headed_gull_Finland_LI7897_23_23VIR6803-15_2023_H5N1_2023-07-11
A_black-headed_gull_Finland_LI7898_23_23VIR6803-16_2023_H5N1_2023-07-11
A_black-headed_gull_Finland_LI7896_23_23VIR6803-14_2023_H5N1_2023-07-11
A_arctic-fox_Finland_648_2023_H5N1_2023-07-28_FarmU
A_arctic-fox_Finland_650_2023_H5N1_2023-07-28_FarmU
A_black-headed_gull_Finland_8134_23VIR7026-6_2023_H5N1_2023-07-21
A_black-headed_gull_Finland_8135_2023_H5N1_2023-07-193
A_black-headed_gull_Finland_8250_2023_H5N1_2023-07-20
A_silver-fox_Finland_554_2023_H5N1_2023-07-17_FarmI
A_red-fox_Finland_553_2023_H5N1_2023-07-17_FarmI
A_black-headed_gull_Finland_8241_2023_H5N1_2023-07-20
A_otter_Finland_1387_2023_H5N1_2023-08-13
A_blue_fox_Finland_2023AI06876_071_2023_H5N1_2023-08-04_FarmE
A_arctic-fox_Finland_856_2023_H5N1_2023-08-10_FarmE
A_black-headed_gull_Finland_8254_23VIR7026-19_2023_H5N1_2023-07-21
A_Black-headed_Gull_Netherlands_81_2023_H5N1_2023-06-10
A_Vulpes_vulpes_Belgium_01924_0003_2023_H5N1_2023-03-02
A_Falco_peregrinus_Belgium_03518_0004_2023_H5N1_2023-04-11
A_Falco_peregrinus_Belgium_04695_0002_2023_H5N1_2023-05-07
A_Gallus_gallus_Belgium_01742_0001_2023_H5N1_2023-02-27
A_Vulpes_vulpes_Belgium_03117_0004_2023_H5N1_2023-03-29
A_Anser_anser_Belgium_00286_0004_2023_H5N1_2023-01-02
A_Vulpes_vulpes_Belgium_01295_0002_2023_H5N1_2023-02-10
A_peregrine_falcon_Czech_Republic_7735-2_2023_H5N1_2023-05-17
A_Falco_peregrinus_Belgium_01145_0004_2023_H5N1_2023-02-07
A_black-headed_gull_Czech_Republic_6634-4_2023_H5N1_2023-04-24
A_black-headed_gull_Czech_Republic_6634-3_2023_H5N1_2023-04-24
A_peregrine_falcon_Czech_Republic_7735-1_2023_H5N1_2023-05-17
A_Vulpes_Vulpes_Belgium_03703_0007_2023_H5N1_2023-04-14
A_Meleagris_gallopavo_Belgium_14826_0002_2022_H5N1_2022-12-24
A_black-headed_gull_Czech_Republic_6634-5_2023_H5N1_2023-05-02
A_black-headed_gull_Czech_Republic_5295-3_2023_H5N1_2023-04-03
A_Chroicocephalus_ridibundus_Belgium_00226_0001_2023_H5N1_2023-01-02
A_peregrine_falcon_Czech_Republic_7735-2_chymus_2023_H5N1_2023-05-17
A_Chroicocephalus_ridibundus_Belgium_01145_0008_2023_H5N1_2023-02-07
A_fox_France_23P002844_2023_H5N1_2023-02-10
A_black-headed_gull_Czech_Republic_6634-1_2023_H5N1_2023-04-24
A_black-headed_gull_Czech_Republic_7334-5_2023_H5N1_2023-05-11
A_Chroicocephalus_ridibundus_Belgium_01145_0006_2023_H5N1_2023-02-07
A_black-headed_gull_Czech_Republic_6633-1_2023_H5N1_2023-04-24
A_Vulpes_vulpes_Belgium_02359_0006_2023_H5N1_2023-03-14
A_Gallus_gallus_Belgium_14828_0003_2022_H5N1_2022-12-27
A_Vulpes_Vulpes_Belgium_04016_0001_2023_H5N1_2023-04-24
A_turkey_Czech_Republic_6734_orig_2023_H5N1_2023-05-02
A_turkey_Czech_Republic_7123-2_2023_H5N1_2023-05-05
A_turkey_Czech_Republic_7124-1_2023_H5N1_2023-05-05
A_turkey_Czech_Republic_7124-2_2023_H5N1_2023-05-05
A_turkey_Czech_Republic_7123-1_2023_H5N1_2023-05-05
A_Falco_peregrinus_Belgium_01823_0003_2023_H5N1_2023-03-01
A_Vulpes_vulpes_Belgium_01784_0008_2023_H5N1_2023-02-27
A_black-headed_gull_Czech_Republic_6577-3_2023_H5N1_2023-04-24
A_Vulpes_vulpes_Belgium_03117_0001_2023_H5N1_2023-03-29
A_black-headed_gull_Spain_512-7_23VIR1687-7_2023_H5N1_2023-01-31
A_Black-headed_gull_Netherlands_39_2023_H5N1_2023-04-17
A_black-headed_gull_Czech_Republic_5152-2_2023_H5N1_2023-04-04
A_black-headed_gull_Czech_Republic_5152-5_2023_H5N1_2023-04-04
A_peregrine_falcon_Czech_Republic_7459-3_2023_H5N1_2023-05-15
A_peregrine_falcon_Czech_Republic_7459-1_2023_H5N1_2023-05-15
A_black-headed_gull_Czech_Republic_5695-1_2023_H5N1_2023-04-13
A_black-headed_gull_Czech_Republic_5487-2_2023_H5N1_2023-04-12
A_black-headed_gull_Czech_Republic_5695-8_2023_H5N1_2023-04-13
A_black-headed_gull_Czech_Republic_5695-7_2023_H5N1_2023-04-13
A_black-headed_gull_Czech_Republic_5695-5_2023_H5N1_2023-04-13
A_black-headed_gull_Czech_Republic_5425-3_2023_H5N1_2023-04-11
A_black-headed_gull_Czech_Republic_5152-3_2023_H5N1_2023-04-04
A_common_tern_Czech_Republic_8807-4_2023_H5N1_2023-05-05
A_black-headead_gull_Spain_416-11_23VIR1687-6_2023_H5N1_2023-01-16
A_black-headed_gull_Czech_Republic_5452-1_2023_H5N1_2023-04-11
A_black-headead_gull_Spain_824-9_23VIR1687-24_2023_H5N1_2023-02-09
A_Pavo_cristatus_Belgium_02559_0001_2023_H5N1_2023-03-17
A_Black-headed_gull_Netherlands_17_2023_H5N1_2023-03-05
A_Black-headed_gull_Netherlands_8_2023_H5N1_2023-02-20
A_domestic_duck_England_049343_2023_H5N1_2023-03-30
A_guinea_fowl_England_049422_2023_H5N1_2023-03-30
A_Black-headed_Gull_Netherlands_95_2023_H5N1_2023-06-18
A_Vulpes_vulpes_Belgium_01784_0010_2023_H5N1_2023-02-27
A_black-headead_gull_Spain_171-1_23VIR1687-20_2023_H5N1_2023-01-09
A_black-headed_gull_Czech_Republic_5695-2_2023_H5N1_2023-04-13
A_black-headed_gull_Czech_Republic_6947-3_2023_H5N1_2023-05-03
A_black-headed_gull_Finland_7477_2023_H5N1_2023-06-25
A_common_tern_Finland_7479_2023_H5N1_2023-06-25
A_black-headed_gull_Finland_LI7728_23_23VIR6803-9_2023_H5N1_2023-07-03
A_great_crested_grebe_Finland_8111_23VIR7026-21_2023_H5N1_2023-07-18
A_canada_goose_Finland_8109_23VIR7026-20_2023_H5N1_2023-07-18
A_Black-headed_Gull_England_060002_2023_H5N1_2023-04-20
A_Black-headed_Gull_England_259880_2023_H5N1_2023-05-08
A_Chroicocephalus_ridibundus_Belgium_00132_0002_2022_H5N1_2022-12-28
A_European_Herring_Gull_Netherlands_7_2022_H5N1_2022-06-07
A_Black-headed_Gull_England_399994_2023_H5N1_2023-04-13
A_Black-headed_Gull_England_259311_2023_H5N1_2023-04-14
A_Gallus_gallus_Belgium_01393_0001_2023_H5N1_2023-02-17
A_Black-headed_Gull_England_125722_2023_H5N1_2023-05-03
A_Common_Tern_Netherlands_6_2023_H5N1_2023-05-31
A_Gallus_gallus_Belgium_01172_0001_2023_H5N1_2023-02-06
A_Vulpes_vulpes_Belgium_01784_0004_2023_H5N1_2023-02-27
A_Black-headed_Gull_Netherlands_89_2023_H5N1_2023-06-16
A_Common_Tern_Netherlands_12_2023_H5N1_2023-06-20
A_Black-headed_Gull_England_400845_2023_H5N1_2023-05-13
A_Black-headed_Gull_England_126426_2023_H5N1_2023-05-30
A_Falco_peregrinus_Belgium_02026_0003_2023_H5N1_2023-03-07
A_seagull_Spain_4621-1_23VIR1687-12_2022_H5N1_2022-12-02
A_Common_Tern_Netherlands_2_2023_H5N1_2023-05-27
A_European_Herring_Gull_Netherlands_13_2022_H5N1_2022-08-19
A_gull_France_22P015977_2022_H5N1_2022-05-11
A_Common_Tern_Netherlands_28_2022_H5N1_2022-08-12
A_Black-headed_Gull_England_259288_2023_H5N1_2023-04-14
A_chicken_England_062653_2023_H5N1_2023-04-28
A_England_231990002_2023_H5N1_2023-05-06
A_Mediterranean_Gull_Netherlands_4_2023_H5N1_2023-05-26
A_Black-headed_Gull_Netherlands_63_2023_H5N1_2023-05-19
A_chicken_England_062635_2023_H5N1_2023-04-28
A_Black-headed_Gull_Netherlands_84_2023_H5N1_2023-06-20
A_Larus_argentatus_Belgium_9013_0001_2022_H5N1_2022-07-08
A_Numenius_arquata_Belgium_01456_0003_2023_H5N1_2023-02-16
A_seagull_Spain_22VIR12774-15_4347-15_2022_H5N1_2022-11-17
A_Black-Headed_Gull_England_400174_2023_H5N1_2023-04-18
A_chicken_England_062649_2023_H5N1_2023-04-28
A_Black-Headed_Gull_England_125550_2023_H5N1_2023-04-28
A_Black-headed_Gull_Netherlands_68_2023_H5N1_2023-05-05
A_Black-Headed_Gull_England_076101_2023_H5N1_2023-05-25
A_mink_Finland_3076_2023_H5N1_2023-09-20_FarmAA
A_mink_Finland_3080_2023_H5N1_2023-09-20_FarmAA
A_mink_Finland_3081_2023_H5N1_2023-09-20_FarmAA
A_mink_Finland_3079_2023_H5N1_2023-09-20_FarmAA
A_mink_Finland_3077_2023_H5N1_2023-09-20_FarmAA
A_mink_Finland_3078_2023_H5N1_2023-09-20_FarmAA
A_mink_Finland_3082_2023_H5N1_2023-09-20_FarmAA
A_black-headed_gull_Finland_9079_2023_H5N1_2023-07-28
A_herring_gull_Finland_10812_2023_H5N1_2023-09-18
A_Caspian_gull_Netherlands_4_2023_H5N1_2023-06-09
A_Black-headed_Gull_Netherlands_55_2023_H5N1_2023-05-12
A_Black-headed_Gull_Netherlands_103_2023_H5N1_2023-06-26
A_pheasant_Scotland_103940_2023_H5N1_2023-08-07
A_pheasant_Scotland_103940_2023_H5N1_2023-08-07
A_black-legged_kittiwake_Finland_8808_2023_H5N1_2023-07-28
A_red_fox_Norway_2023_80_49_2_2023_H5N1_2023-06-27
A_black-legged_kittiwake_Finland_8772_2023_H5N1_2023-07-23
A_herring_gull_Finland_10450_2023_H5N1_2023-08-29
A_Peregrine_Falcon_England_261694_2023_H5N1_2023-07-25
A_Kittiwake_England_331460_2023_H5N1_2023-07-28
A_Peregrine_falcon_England_261690_2023_H5N1_2023-07-25
A_raxorbill_Finland_9072_2023_H5N1_2023-07-27_Geta
H5N1 Finland 2021
A_golden_eagle_Finland_9378_21VIR7689-12_2021_H5N1_2021-07-01
A_white-tailed_eagle_Finland_6984_21VIR7689-10_2021_H5N1_2021-06-01
A_barnacle_goose_Finland_6955_21VIR7689-9_2021_H5N1_2021-06-01
A_white-tailed_eagle_Finland_10644_2023_H5N1_2023-09-02
A_white-tailed_eagle_Finland_8154_23VIR7026-22_2023_H5N1_2023-07-18
A_black-headed_gull_Finland_8107_23VIR7026-7_2023_H5N1_2023-07-20
A_white-tailed_eagle_Finland_L17800_23_23VIR6803-10_2023_H5N1_2023-06-28
A_mute_swan_Denmark_06134-1-02_2023-07-27_H5N1_2023-07-27
A_mute_swan_Denmark_06339-1-01_2023-08-05_H5N1_2023-08-05
A_Domestic_duck_Wales_020076_2023_H5N1_2023-02-05
A_Chicken_England_155942_2022_H5N1_2022-11-20
A_Turkey_England_155093_2022_H5N1_2022-11-20
A_chicken_Wales_022780_2023_H5N1_2023-02-11
H5N8 Finland 2021
0.02

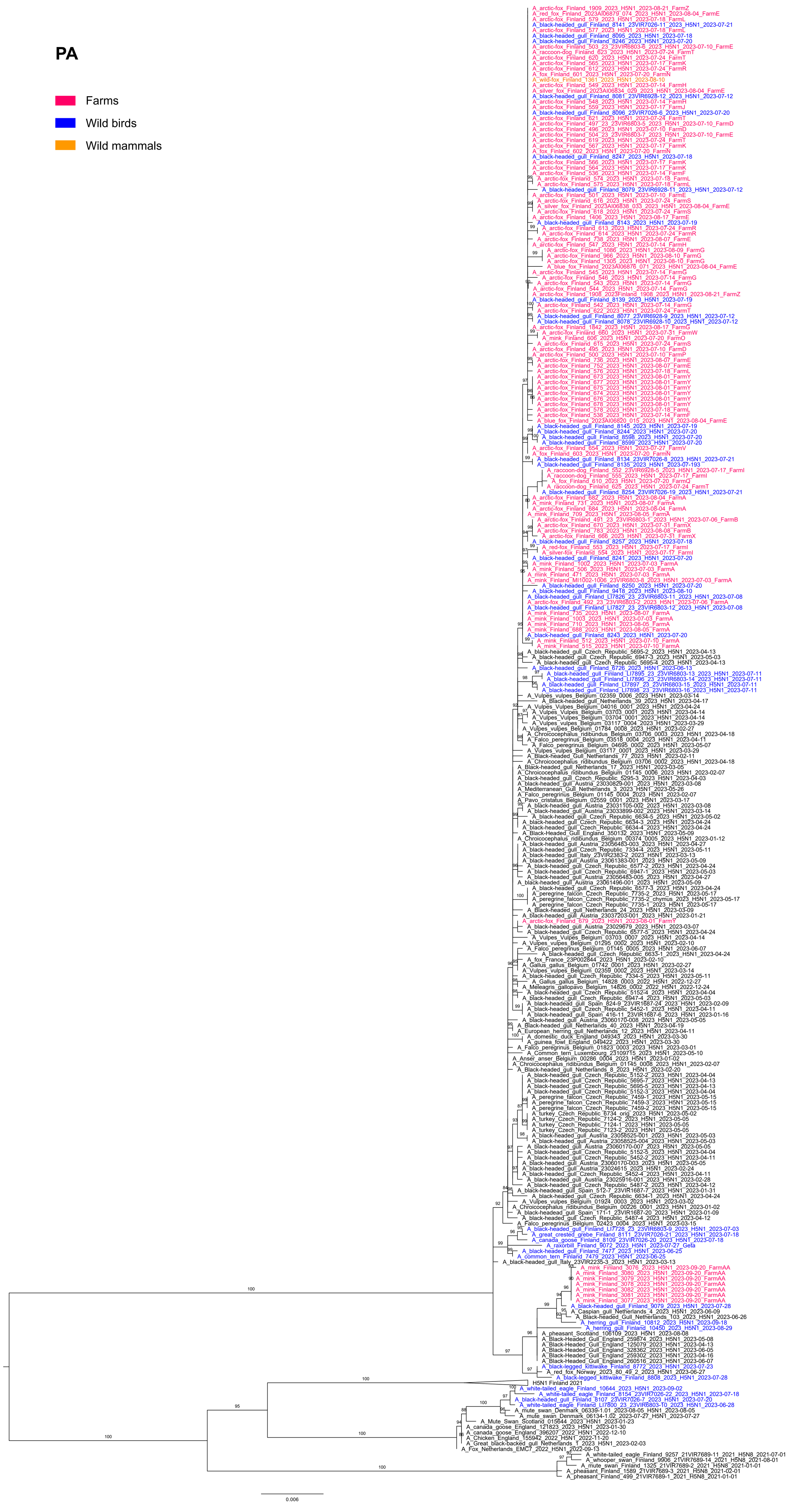
PA
Farms
Wild birds
Wild mammals
A_arctic-fox_Finland_1909_2023_H5N1_2023-08-21_FarmZ
A_red_fox_Finland_2023AI06879_074_2023_H5N1_2023-08-04_FarmE
A_arctic-fox_Finland_579_2023_H5N1_2023-07-18_FarmL
A_black-headed_gull_Finland_8141_23VIR7026-11_2023_H5N1_2023-07-21
A_arctic-fox_Finland_577_2023_H5N1_2023-07-18_FarmL
A_black-headed_gull_Finland_8095_2023_H5N1_2023-07-18
A_black-headed_gull_Finland_8246_2023_H5N1_2023-07-20
A_arctic-fox_Finland_503_23_23VIR6803-9_2023_H5N1_2023-07-10_FarmE
A_raccoon-dog_Finland_623_2023_H5N1_2023-07-24_FarmT
A_arctic-fox_Finland_620_2023_H5N1_2023-07-24_FarmT
A_arctic-fox_Finland_565_2023_H5N1_2023-07-17_FarmK
A_arctic-fox_Finland_612_2023_H5N1_2023-07-24_FarmR
A_fox_Finland_601_2023_H5N1_2023-07-20_FarmN
A_wild-fox_Finland_1361_2023_H5N1_2023-08-10
A_arctic-fox_Finland_549_2023_H5N1_2023-07-14_FarmH
A_silver_fox_Finland_2023AI06834_023_2023_H5N1_2023-08-04_FarmE
A_black-headed_gull_Finland_8081_23VIR6928-12_2023_H5N1_2023-07-12
A_arctic-fox_Finland_548_2023_H5N1_2023-07-14_FarmH
A_arctic-fox_Finland_559_2023_H5N1_2023-07-17_FarmJ
A_black-headed_gull_Finland_8096_23VIR7026-6_2023_H5N1_2023-07-20
A_arctic-fox_Finland_621_2023_H5N1_2023-07-24_FarmT
A_arctic-fox_Finland_497_23_23VIR6803-5_2023_H5N1_2023-07-10_FarmD
A_arctic-fox_Finland_496_2023_H5N1_2023-07-10_FarmD
A_arctic-fox_Finland_504_23_23VIR6803-7_2023_H5N1_2023-07-10_FarmE
A_arctic-fox_Finland_619_2023_H5N1_2023-07-24_FarmT
A_arctic-fox_Finland_567_2023_H5N1_2023-07-17_FarmK
A_fox_Finland_602_2023_H5N1_2023-07-20_FarmN
A_black-headed_gull_Finland_8247_2023_H5N1_2023-07-18
A_arctic-fox_Finland_566_2023_H5N1_2023-07-17_FarmK
A_arctic-fox_Finland_564_2023_H5N1_2023-07-17_FarmK
A_arctic-fox_Finland_536_2023_H5N1_2023-07-14_FarmF
A_arctic-fox_Finland_574_2023_H5N1_2023-07-18_FarmL
A_arctic-fox_Finland_575_2023_H5N1_2023-07-18_FarmL
A_black-headed_gull_Finland_8079_23VIR6928-11_2023_H5N1_2023-07-12
A_arctic-fox_Finland_501_2023_H5N1_2023-07-10_FarmE
A_arctic-fox_Finland_676_2023_H5N1_2023-07-24_FarmS
A_silver_fox_Finland_2023AI06838_033_2023_H5N1_2023-08-04_FarmE
A_arctic-fox_Finland_818_2023_H5N1_2023-07-24_FarmS
A_arctic-fox_Finland_1406_2023_H5N1_2023-08-17_FarmE
A_black-headed_gull_Finland_8143_2023_H5N1_2023-07-19
A_arctic-fox_Finland_613_2023_H5N1_2023-07-24_FarmR
A_arctic-fox_Finland_614_2023_H5N1_2023-07-24_FarmR
A_arctic-fox_Finland_738_2023_H5N1_2023-08-07_FarmE
A_arctic-fox_Finland_547_2023_H5N1_2023-07-14_FarmH
A_arctic-fox_Finland_1086_2023_H5N1_2023-08-09_FarmG
A_arctic-fox_Finland_966_2023_H5N1_2023-08-10_FarmG
A_arctic-fox_Finland_1305_2023_H5N1_2023-08-10_FarmG
A_blue_fox_Finland_2023AI06876_071_2023_H5N1_2023-08-04_FarmE
A_arctic-fox_Finland_545_2023_H5N1_2023-07-14_FarmG
A_arctic-fox_Finland_546_2023_H5N1_2023-07-14_FarmG
A_arctic-fox_Finland_543_2023_H5N1_2023-07-14_FarmG
A_arctic-fox_Finland_544_2023_H5N1_2023-07-14_FarmG
A_arctic-fox_Finland_1908_2023Finland_1908_2023_H5N1_2023-08-21_FarmZ
A_black-headed_gull_Finland_8139_2023_H5N1_2023-07-19
A_arctic-fox_Finland_542_2023_H5N1_2023-07-14_FarmG
A_arctic-fox_Finland_622_2023_H5N1_2023-07-24_FarmT
A_black-headed_gull_Finland_8077_23VIR6928-9_2023_H5N1_2023-07-12
A_black-headed_gull_Finland_8078_23VIR6928-10_2023_H5N1_2023-07-12
A_arctic-fox_Finland_1842_2023_H5N1_2023-08-17_FarmG
A_arctic-fox_Finland_660_2023_H5N1_2023-07-31_FarmW
A_mink_Finland_606_2023_H5N1_2023-07-20_FarmO
A_arctic-fox_Finland_615_2023_H5N1_2023-07-24_FarmS
A_arctic-fox_Finland_499_2023_H5N1_2023-07-10_FarmD
A_arctic-fox_Finland_500_2023_H5N1_2023-07-10_FarmP
A_arctic-fox_Finland_736_2023_H5N1_2023-08-07_FarmE
A_arctic-fox_Finland_752_2023_H5N1_2023-08-07_FarmE
A_arctic-fox_Finland_576_2023_H5N1_2023-07-18_FarmL
A_arctic-fox_Finland_673_2023_H5N1_2023-08-01_FarmY
A_arctic-fox_Finland_677_2023_H5N1_2023-08-01_FarmY
A_arctic-fox_Finland_675_2023_H5N1_2023-08-01_FarmY
A_arctic-fox_Finland_674_2023_H5N1_2023-08-01_FarmY
A_arctic-fox_Finland_676_2023_H5N1_2023-08-01_FarmY
A_arctic-fox_Finland_678_2023_H5N1_2023-08-01_FarmY
A_arctic-fox_Finland_578_2023_H5N1_2023-07-18_FarmL
A_arctic-fox_Finland_538_2023_H5N1_2023-07-14_FarmF
A_blue_fox_Finland_2023AI06820_015_2023_H5N1_2023-08-04_FarmE
A_black-headed_gull_Finland_8145_2023_H5N1_2023-07-19
A_black-headed_gull_Finland_8244_2023_H5N1_2023-07-20
A_black-headed_gull_Finland_8598_2023_H5N1_2023-07-20
A_black-headed_gull_Finland_8599_2023_H5N1_2023-07-20
A_arctic-fox_Finland_554_2023_H5N1_2023-07-27_FarmV
A_fox_Finland_603_2023_H5N1_2023-07-20_FarmN
A_black-headed_gull_Finland_8134_23VIR7026-8_2023_H5N1_2023-07-21
A_black-headed_gull_Finland_8135_2023_H5N1_2023-07-193
A_raccoon-dog_Finland_552_23VIR6928-5_2023_H5N1_2023-07-17_FarmI
A_raccoon-dog_Finland_555_2023_H5N1_2023-07-17_FarmI
A_fox_Finland_610_2023_H5N1_2023-07-20_FarmQ
A_raccoon-dog_Finland_625_2023_H5N1_2023-07-24_FarmT
A_black-headed_gull_Finland_8254_23VIR7026-19_2023_H5N1_2023-07-21
A_arctic-fox_Finland_682_2023_H5N1_2023-08-04_FarmA
A_mink_Finland_731_2023_H5N1_2023-08-07_FarmA
A_arctic-fox_Finland_684_2023_H5N1_2023-08-04_FarmA
A_mink_Finland_709_2023_H5N1_2023-08-05_FarmA
A_arctic-fox_Finland_491_23_23VIR6803-1_2023_H5N1_2023-07-06_FarmB
A_arctic-fox_Finland_670_2023_H5N1_2023-07-31_FarmX
A_arctic-fox_Finland_783_2023_H5N1_2023-08-08_FarmB
A_arctic-fox_Finland_666_2023_H5N1_2023-07-31_FarmX
A_black-headed_gull_Finland_8257_2023_H5N1_2023-07-18
A_red-fox_Finland_553_2023_H5N1_2023-07-17_FarmI
A_silver-fox_Finland_554_2023_H5N1_2023-07-17_FarmI
A_black-headed_gull_Finland_8241_2023_H5N1_2023-07-20
A_mink_Finland_1002_2023_H5N1_2023-07-03_FarmA
A_mink_Finland_506_2023_H5N1_2023-07-03_FarmA
A_mink_Finland_471_2023_H5N1_2023-07-03_FarmA
A_mink_Finland_MI1002-1006_23VIR6803-8_2023_H5N1_2023-07-03_FarmA
A_black-headed_gull_Finland_8250_2023_H5N1_2023-07-20
A_black-headed_gull_Finland_9418_2023_H5N1_2023-08-10
A_black-headed_gull_Finland_LI7826_23_23VIR6803-11_2023_H5N1_2023-07-08
A_arctic-fox_Finland_492_23_23VIR6803-2_2023_H5N1_2023-07-06_FarmA
A_black-headed_gull_Finland_LI7827_23_23VIR6803-12_2023_H5N1_2023-07-08
A_mink_Finland_735_2023_H5N1_2023-08-07_FarmA
A_mink_Finland_1003_2023_H5N1_2023-07-03_FarmA
A_mink_Finland_710_2023_H5N1_2023-08-05_FarmA
A_mink_Finland_698_2023_H5N1_2023-08-05_FarmA
A_black-headed_gull_Finland_8243_2023_H5N1_2023-07-20
A_mink_Finland_512_2023_H5N1_2023-07-10_FarmA
A_mink_Finland_515_2023_H5N1_2023-07-10_FarmA
A_black-headed_gull_Czech_Republic_5695-2_2023_H5N1_2023-04-13
A_black-headed_gull_Czech_Republic_6947-3_2023_H5N1_2023-05-03
A_black-headed_gull_Czech_Republic_5695-4_2023_H5N1_2023-04-13
A_black-headed_gull_Finland_8726_2023_H5N1_2023-06-13
A_black-headed_gull_Finland_LI7895_23_23VIR6803-13_2023_H5N1_2023-07-11
A_black-headed_gull_Finland_LI7896_23_23VIR6803-14_2023_H5N1_2023-07-11
A_black-headed_gull_Finland_LI7897_23_23VIR6803-15_2023_H5N1_2023-07-11
A_black-headed_gull_Finland_LI7898_23_23VIR6803-16_2023_H5N1_2023-07-11
A_Vulpes_vulpes_Belgium_02359_0006_2023_H5N1_2023-05-14
A_Black-headed_gull_Netherlands_39_2023_H5N1_2023-04-17
A_Vulpes_Vulpes_Belgium_04016_0001_2023_H5N1_2023-04-24
A_Vulpes_Vulpes_Belgium_03703_0001_2023_H5N1_2023-04-14
A_Vulpes_Vulpes_Belgium_03704_0001_2023_H5N1_2023-04-14
A_Vulpes_vulpes_Belgium_03117_0004_2023_H5N1_2023-03-29
A_Vulpes_vulpes_Belgium_01784_0008_2023_H5N1_2023-02-27
A_Chroicocephalus_ridibundus_Belgium_03706_0003_2023_H5N1_2023-04-18
A_Falco_peregrinus_Belgium_03518_0004_2023_H5N1_2023-04-11
A_Falco_peregrinus_Belgium_04695_0002_2023_H5N1_2023-05-07
A_Vulpes_vulpes_Belgium_03117_0001_2023_H5N1_2023-03-29
A_Black-headed_Gull_Netherlands_77_2023_H5N1_2023-02-11
A_Chroicocephalus_ridibundus_Belgium_03706_0002_2023_H5N1_2023-04-18
A_Black-headed_gull_Netherlands_17_2023_H5N1_2023-03-05
A_Chroicocephalus_ridibundus_Belgium_01145_0006_2023_H5N1_2023-02-07
A_black-headed_gull_Czech_Republic_5295-3_2023_H5N1_2023-04-03
A_black-headed_gull_Austria_23030829-001_2023_H5N1_2023-03-08
A_Mediterranean_Gull_Netherlands_3_2023_H5N1_2023-05-26
A_Falco_peregrinus_Belgium_01145_0004_2023_H5N1_2023-02-07
A_Pavo_cristatus_Belgium_02559_0001_2023_H5N1_2023-03-17
A_black-headed_gull_Austria_23031105-002_2023_H5N1_2023-03-08
A_black-headed_gull_Austria_23033899-002_2023_H5N1_2023-03-14
A_black-headed_gull_Czech_Republic_6634-5_2023_H5N1_2023-05-02
A_black-headed_gull_Czech_Republic_6634-3_2023_H5N1_2023-04-24
A_black-headed_gull_Czech_Republic_6634-4_2023_H5N1_2023-04-24
A_Black-headed_Gull_England_350132_2023_H5N1_2023-05-09
A_Chroicocephalus_ridibundus_Belgium_00374_0005_2023_H5N1_2023-01-12
A_black-headed_gull_Austria_23056483-003_2023_H5N1_2023-04-27
A_black-headed_gull_Czech_Republic_7334-4_2023_H5N1_2023-05-11
A_black-headed_gull_Italy_23VIR2383-2_2023_H5N1_2023-03-13
A_black-headed_gull_Austria_23061383-001_2023_H5N1_2023-05-09
A_black-headed_gull_Czech_Republic_6577-2_2023_H5N1_2023-04-24
A_black-headed_gull_Czech_Republic_6947-1_2023_H5N1_2023-05-03
A_black-headed_gull_Austria_23056483-005_2023_H5N1_2023-04-27
A_black-headed_gull_Austria_23061496-001_2023_H5N1_2023-05-09
A_black-headed_gull_Czech_Republic_6577-3_2023_H5N1_2023-04-24
A_peregrine_falcon_Czech_Republic_7735-2_2023_H5N1_2023-05-17
A_peregrine_falcon_Czech_Republic_7735-2_chymis_2023_H5N1_2023-05-17
A_peregrine_falcon_Czech_Republic_7735-1_2023_H5N1_2023-05-17
A_Black-headed_gull_Netherlands_24_2023_H5N1_2023-03-09
A_black-headed_gull_Austria_23037203-001_2023_H5N1_2023-01-21
A_arctic-fox_Finland_679_2023_H5N1_2023-08-01_FarmY
A_black-headed_gull_Austria_23029679_2023_H5N1_2023-03-07
A_black-headed_gull_Czech_Republic_6577-5_2023_H5N1_2023-04-24
A_Vulpes_Vulpes_Belgium_03703_0007_2023_H5N1_2023-04-14
A_Vulpes_vulpes_Belgium_01295_0002_2023_H5N1_2023-02-10
A_Falco_peregrinus_Belgium_01145_0005_2023_H5N1_2023-06-07
A_black-headed_gull_Czech_Republic_6633-1_2023_H5N1_2023-04-24
A_fox_France_23P002844_2023_H5N1_2023-02-10
A_Gallus_gallus_Belgium_01742_0001_2023_H5N1_2023-02-27
A_Vulpes_vulpes_Belgium_02359_0002_2023_H5N1_2023-03-14
A_black-headed_gull_Czech_Republic_7334-5_2023_H5N1_2023-05-11
A_Gallus_gallus_Belgium_14828_0003_2022_H5N1_2022-12-27
A_Meleagris_gallopavo_Belgium_14826_0002_2022_H5N1_2022-12-24
A_black-headed_gull_Czech_Republic_5152-4_2023_H5N1_2023-04-04
A_black-headed_gull_Czech_Republic_6947-4_2023_H5N1_2023-05-03
A_black-headed_gull_Spain_824-9_23VIR1687-24_2023_H5N1_2023-02-09
A_black-headed_gull_Czech_Republic_5452-1_2023_H5N1_2023-04-11
A_black-headed_gull_Spain_416-11_23VIR1687-6_2023_H5N1_2023-01-16
A_black-headed_gull_Austria_23060170-008_2023_H5N1_2023-05-05
A_Black-headed_gull_Netherlands_40_2023_H5N1_2023-04-19
A_European_herring_gull_Netherlands_12_2023_H5N1_2023-04-11
A_domestic_duck_England_049343_2023_H5N1_2023-03-30
A_guinea_fowl_England_049422_2023_H5N1_2023-03-30
A_Falco_peregrinus_Belgium_01823_0003_2023_H5N1_2023-03-01
A_Common_tern_Luxembourg_23109715_2023_H5N1_2023-05-10
A_Anser_anser_Belgium_00286_0004_2023_H5N1_2023-01-02
A_Chroicocephalus_ridibundus_Belgium_01145_0008_2023_H5N1_2023-02-07
A_Black-headed_gull_Netherlands_8_2023_H5N1_2023-02-20
A_black-headed_gull_Czech_Republic_5152-2_2023_H5N1_2023-04-04
A_black-headed_gull_Czech_Republic_5695-7_2023_H5N1_2023-04-13
A_black-headed_gull_Czech_Republic_5695-5_2023_H5N1_2023-04-13
A_black-headed_gull_Czech_Republic_5152-3_2023_H5N1_2023-04-04
A_peregrine_falcon_Czech_Republic_7459-1_2023_H5N1_2023-05-15
A_peregrine_falcon_Czech_Republic_7459-3_2023_H5N1_2023-05-15
A_peregrine_falcon_Czech_Republic_7459-2_2023_H5N1_2023-05-15
A_turkey_Czech_Republic_6734_orig_2023_H5N1_2023-05-02
A_turkey_Czech_Republic_7124-2_2023_H5N1_2023-05-05
A_turkey_Czech_Republic_7124-1_2023_H5N1_2023-05-05
A_turkey_Czech_Republic_7123-2_2023_H5N1_2023-05-05
A_black-headed_gull_Austria_23058525-001_2023_H5N1_2023-05-03
A_black-headed_gull_Austria_23058525-004_2023_H5N1_2023-05-03
A_black-headed_gull_Austria_23060170-007_2023_H5N1_2023-05-05
A_black-headed_gull_Czech_Republic_5152-5_2023_H5N1_2023-04-04
A_black-headed_gull_Czech_Republic_5452-2_2023_H5N1_2023-04-11
A_black-headed_gull_Austria_23060170-003_2023_H5N1_2023-05-05
A_black-headed_gull_Austria_23024615_2023_H5N1_2023-02-24
A_black-headed_gull_Czech_Republic_5452-4_2023_H5N1_2023-04-11
A_black-headed_gull_Austria_23025916-001_2023_H5N1_2023-02-28
A_black-headed_gull_Czech_Republic_5487-2_2023_H5N1_2023-04-12
A_black-headed_gull_Spain_512-7_23VIR1687-7_2023_H5N1_2023-01-31
A_black-headed_gull_Czech_Republic_6634-1_2023_H5N1_2023-04-24
A_Vulpes_vulpes_Belgium_01924_0003_2023_H5N1_2023-03-02
A_Chroicocephalus_ridibundus_Belgium_00226_0001_2023_H5N1_2023-01-02
A_black-headed_gull_Spain_171-1_23VIR1687-20_2023_H5N1_2023-01-09
A_black-headed_gull_Czech_Republic_5487-4_2023_H5N1_2023-04-12
A_Falco_peregrinus_Belgium_02423_0004_2023_H5N1_2023-03-15
A_black-headed_gull_Finland_LI7728_23_23VIR6803-9_2023_H5N1_2023-07-03
A_great_crested_grebe_Finland_8111_23VIR7026-21_2023_H5N1_2023-07-18
A_canada_goose_Finland_8109_23VIR7026-20_2023_H5N1_2023-07-18
A_raxorbill_Finland_9072_2023_H5N1_2023-07-27_Geta
A_black-headed_gull_Finland_7477_2023_H5N1_2023-06-25
A_common_tern_Finland_7479_2023_H5N1_2023-06-25
A_black-headed_gull_Italy_23VIR2235-3_2023_H5N1_2023-03-13
A_mink_Finland_3076_2023_H5N1_2023-09-20_FarmAA
A_mink_Finland_3080_2023_H5N1_2023-09-20_FarmAA
A_mink_Finland_3079_2023_H5N1_2023-09-20_FarmAA
A_mink_Finland_3078_2023_H5N1_2023-09-20_FarmAA
A_mink_Finland_3082_2023_H5N1_2023-09-20_FarmAA
A_mink_Finland_3081_2023_H5N1_2023-09-20_FarmAA
A_mink_Finland_3077_2023_H5N1_2023-09-20_FarmAA
A_black-headed_gull_Finland_9079_2023_H5N1_2023-07-28
A_Caspian_gull_Netherlands_4_2023_H5N1_2023-06-09
A_Black-headed_Gull_Netherlands_103_2023_H5N1_2023-06-26
A_herring_gull_Finland_10812_2023_H5N1_2023-09-18
A_herring_gull_Finland_10450_2023_H5N1_2023-08-29
A_pheasant_Scotland_108109_2023_H5N1_2023-08-08
A_Black-Headed_Gull_England_259874_2023_H5N1_2023-05-08
A_Black-Headed_Gull_England_125079_2023_H5N1_2023-04-13
A_Black-Headed_Gull_England_328362_2023_H5N1_2023-06-05
A_Black-Headed_Gull_England_259302_2023_H5N1_2023-04-16
A_Black-Headed_Gull_England_260516_2023_H5N1_2023-06-07
A_black-legged_kittiwake_Finland_8772_2023_H5N1_2023-07-23
A_red_fox_Norway_2023_80_49_2_2023_H5N1_2023-08-27
A_black-legged_kittiwake_Finland_8808_2023_H5N1_2023-07-28
H5N1 Finland 2021
A_white-tailed_eagle_Finland_10644_2023_H5N1_2023-09-02
A_white-tailed_eagle_Finland_8154_23VIR7026-22_2023_H5N1_2023-07-18
A_black-headed_gull_Finland_8107_23VIR7026-7_2023_H5N1_2023-07-20
A_white-tailed_eagle_Finland_LI7800_23_23VIR6803-10_2023_H5N1_2023-06-28
A_mute_swan_Denmark_06339-1.01_2023-08-05_H5N1_2023-08-05
A_mute_swan_Denmark_06134-1.02_2023-07-27_H5N1_2023-07-27
A_Mute_Swan_Scotland_015644_2023_H5N1_2023-01-23
A_canada_goose_England_121823_2023_H5N1_2023-01-30
A_canada_goose_England_396207_2022_H5N1_2022-12-10
A_Chicken_England_155942_2022_H5N1_2022-11-20
A_Great_black-backed_gull_Netherlands_1_2023_H5N1_2023-02-03
A_Fox_Netherlands_EMC7_2022_H5N1_2022-09-13
A_white-tailed_eagle_Finland_9257_21VIR7689-11_2021_H5N8_2021-07-01
A_whooper_swan_Finland_9906_21VIR7689-14_2021_H5N8_2021-08-01
A_mute_swan_Finland_1325_21VIR7689-2_2021_H5N8_2021-01-01
A_pheasant_Finland_1589_21VIR7689-3_2021_H5N8_2021-02-01
A_pheasant_Finland_499_21VIR7689-1_2021_H5N8_2021-01-01
0.006

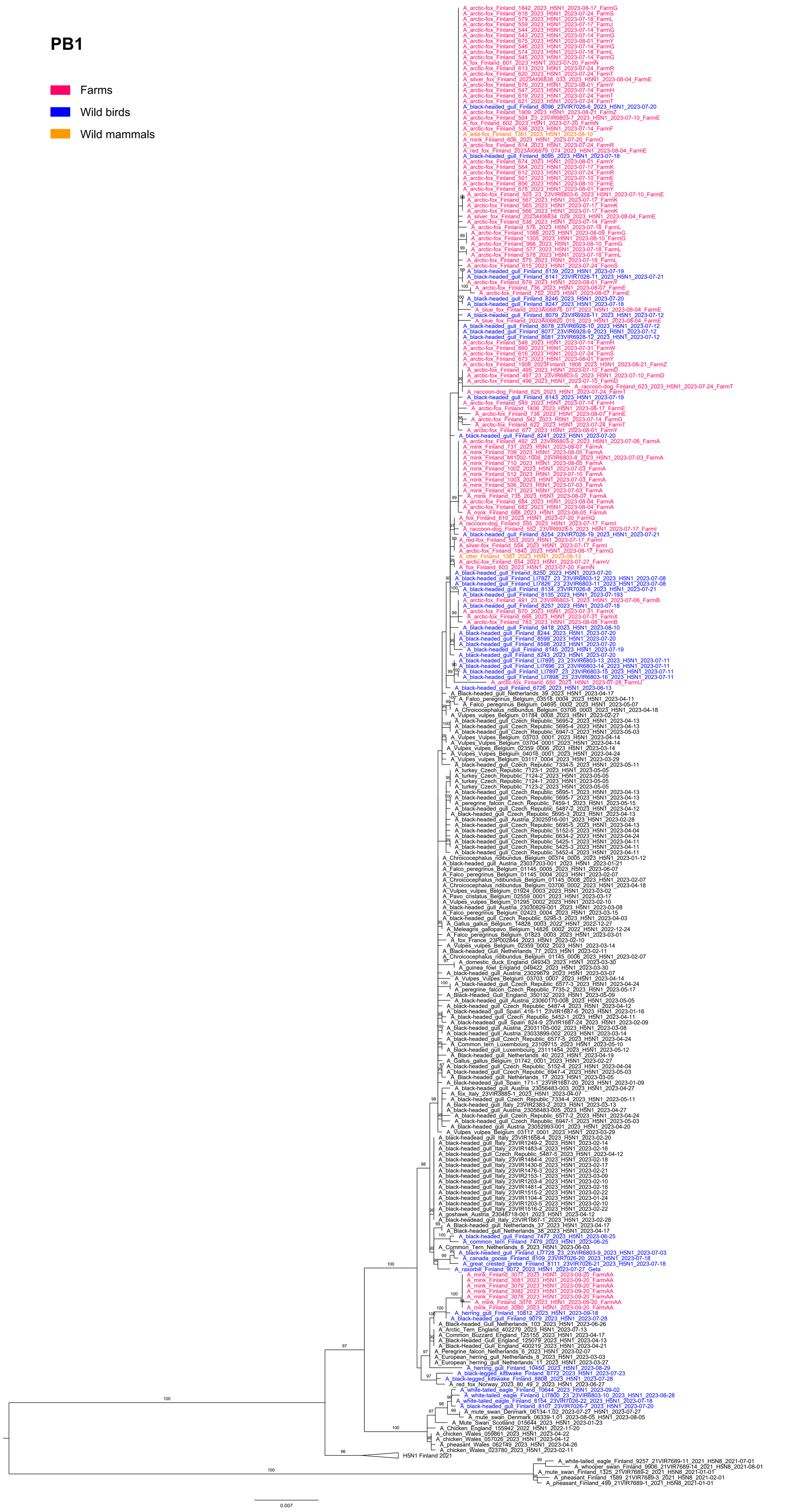
PB1
Farms
Wild birds
Wild mammals
A_arctic-fox_Finland_1842_2023_H5N1_2023-08-17_FarmG
A_arctic-fox_Finland_618_2023_H5N1_2023-07-24_FarmS
A_arctic-fox_Finland_579_2023_H5N1_2023-07-18_FarmL
A_arctic-fox_Finland_559_2023_H5N1_2023-07-17_FarmJ
A_arctic-fox_Finland_544_2023_H5N1_2023-07-14_FarmG
A_arctic-fox_Finland_543_2023_H5N1_2023-07-14_FarmG
A_arctic-fox_Finland_675_2023_H5N1_2023-08-01_FarmY
A_arctic-fox_Finland_546_2023_H5N1_2023-07-14_FarmG
A_arctic-fox_Finland_574_2023_H5N1_2023-07-18_FarmL
A_arctic-fox_Finland_545_2023_H5N1_2023-07-14_FarmG
A_fox_Finland_601_2023_H5N1_2023-07-20_FarmN
A_arctic-fox_Finland_613_2023_H5N1_2023-07-24_FarmR
A_arctic-fox_Finland_620_2023_H5N1_2023-07-24_FarmT
A_silver_fox_Finland_2023AI06838_033_2023_H5N1_2023-08-04_FarmE
A_arctic-fox_Finland_676_2023_H5N1_2023-08-01_FarmY
A_arctic-fox_Finland_547_2023_H5N1_2023-07-14_FarmH
A_arctic-fox_Finland_619_2023_H5N1_2023-07-24_FarmT
A_arctic-fox_Finland_621_2023_H5N1_2023-07-24_FarmT
A_black-headed_gull_Finland_8096_23VIR7026-6_2023_H5N1_2023-07-20
A_arctic-fox_Finland_1909_2023_H5N1_2023-08-21_FarmZ
A_arctic-fox_Finland_504_23_23VIR6803-7_2023_H5N1_2023-07-10_FarmE
A_fox_Finland_602_2023_H5N1_2023-07-20_FarmN
A_arctic-fox_Finland_536_2023_H5N1_2023-07-14_FarmF
A_wild-fox_Finland_1361_2023_H5N1_2023-08-10
A_mink_Finland_608_2023_H5N1_2023-07-20_FarmO
A_arctic-fox_Finland_614_2023_H5N1_2023-07-24_FarmR
A_red_fox_Finland_2023AI06879_074_2023_H5N1_2023-08-04_FarmE
A_black-headed_gull_Finland_8095_2023_H5N1_2023-07-18
A_arctic-fox_Finland_674_2023_H5N1_2023-08-01_FarmY
A_arctic-fox_Finland_564_2023_H5N1_2023-07-17_FarmK
A_arctic-fox_Finland_612_2023_H5N1_2023-07-24_FarmR
A_arctic-fox_Finland_501_2023_H5N1_2023-07-10_FarmE
A_arctic-fox_Finland_856_2023_H5N1_2023-08-10_FarmE
A_arctic-fox_Finland_678_2023_H5N1_2023-08-01_FarmY
A_arctic-fox_Finland_503_23_23VIR6803-6_2023_H5N1_2023-07-10_FarmE
A_arctic-fox_Finland_567_2023_H5N1_2023-07-17_FarmK
A_arctic-fox_Finland_565_2023_H5N1_2023-07-17_FarmK
A_arctic-fox_Finland_566_2023_H5N1_2023-07-17_FarmK
A_silver_fox_Finland_2023AI06834_029_2023_H5N1_2023-08-04_FarmE
A_arctic-fox_Finland_538_2023_H5N1_2023-07-14_FarmF
A_arctic-fox_Finland_576_2023_H5N1_2023-07-18_FarmL
A_arctic-fox_Finland_1088_2023_H5N1_2023-08-09_FarmG
A_arctic-fox_Finland_1305_2023_H5N1_2023-08-10_FarmG
A_arctic-fox_Finland_966_2023_H5N1_2023-08-10_FarmG
A_arctic-fox_Finland_577_2023_H5N1_2023-07-18_FarmL
A_arctic-fox_Finland_578_2023_H5N1_2023-07-18_FarmL
A_arctic-fox_Finland_575_2023_H5N1_2023-07-18_FarmL
A_arctic-fox_Finland_615_2023_H5N1_2023-07-24_FarmS
A_black-headed_gull_Finland_8139_2023_H5N1_2023-07-19
A_black-headed_gull_Finland_8141_23VIR7026-11_2023_H5N1_2023-07-21
A_arctic-fox_Finland_679_2023_H5N1_2023-08-01_FarmY
A_arctic-fox_Finland_736_2023_H5N1_2023-08-07_FarmE
A_arctic-fox_Finland_752_2023_H5N1_2023-08-07_FarmE
A_black-headed_gull_Finland_8246_2023_H5N1_2023-07-20
A_black-headed_gull_Finland_8247_2023_H5N1_2023-07-18
A_blue_fox_Finland_2023AI06876_077_2023_H5N1_2023-08-04_FarmE
A_black-headed_gull_Finland_8079_23VIR6928-11_2023_H5N1_2023-07-12
A_blue_fox_Finland_2023AI06820_015_2023_H5N1_2023-08-04_FarmE
A_black-headed_gull_Finland_8078_23VIR6928-10_2023_H5N1_2023-07-12
A_black-headed_gull_Finland_8077_23VIR6928-9_2023_H5N1_2023-07-12
A_black-headed_gull_Finland_8081_23VIR6928-12_2023_H5N1_2023-07-12
A_arctic-fox_Finland_548_2023_H5N1_2023-07-14_FarmH
A_arctic-fox_Finland_660_2023_H5N1_2023-07-31_FarmW
A_arctic-fox_Finland_616_2023_H5N1_2023-07-24_FarmS
A_arctic-fox_Finland_673_2023_H5N1_2023-08-01_FarmY
A_arctic-fox_Finland_1908_2023Finland_1908_2023_H5N1_2023-08-21_FarmZ
A_arctic-fox_Finland_495_2023_H5N1_2023-07-10_FarmD
A_arctic-fox_Finland_497_23_23VIR6803-5_2023_H5N1_2023-07-10_FarmD
A_arctic-fox_Finland_496_2023_H5N1_2023-07-10_FarmD
A_raccoon-dog_Finland_623_2023_H5N1_2023-07-24_FarmT
A_raccoon-dog_Finland_625_2023_H5N1_2023-07-24_FarmT
A_black-headed_gull_Finland_8143_2023_H5N1_2023-07-19
A_arctic-fox_Finland_549_2023_H5N1_2023-07-14_FarmH
A_arctic-fox_Finland_1406_2023_H5N1_2023-08-17_FarmE
A_arctic-fox_Finland_738_2023_H5N1_2023-08-07_FarmE
A_arctic-fox_Finland_542_2023_H5N1_2023-07-14_FarmG
A_arctic-fox_Finland_622_2023_H5N1_2023-07-24_FarmT
A_arctic-fox_Finland_677_2023_H5N1_2023-08-01_FarmY
A_black-headed_gull_Finland_8241_2023_H5N1_2023-07-20
A_arctic-fox_Finland_492_23_23VIR6803-2_2023_H5N1_2023-07-06_FarmA
A_mink_Finland_731_2023_H5N1_2023-08-07_FarmA
A_mink_Finland_709_2023_H5N1_2023-08-05_FarmA
A_mink_Finland_MI1002-1006_23VIR6803-8_2023_H5N1_2023-07-03_FarmA
A_mink_Finland_710_2023_H5N1_2023-08-05_FarmA
A_mink_Finland_1002_2023_H5N1_2023-07-03_FarmA
A_mink_Finland_512_2023_H5N1_2023-07-10_FarmA
A_mink_Finland_1003_2023_H5N1_2023-07-03_FarmA
A_mink_Finland_506_2023_H5N1_2023-07-03_FarmA
A_mink_Finland_471_2023_H5N1_2023-07-03_FarmA
A_mink_Finland_735_2023_H5N1_2023-08-07_FarmA
A_arctic-fox_Finland_684_2023_H5N1_2023-08-04_FarmA
A_arctic-fox_Finland_682_2023_H5N1_2023-08-04_FarmA
A_mink_Finland_688_2023_H5N1_2023-08-05_FarmA
A_fox_Finland_610_2023_H5N1_2023-07-20_FarmO
A_raccoon-dog_Finland_555_2023_H5N1_2023-07-17_FarmI
A_raccoon-dog_Finland_552_23VIR6928-5_2023_H5N1_2023-07-17_FarmI
A_black-headed_gull_Finland_8254_23VIR7026-19_2023_H5N1_2023-07-21
A_red-fox_Finland_553_2023_H5N1_2023-07-17_FarmI
A_silver-fox_Finland_554_2023_H5N1_2023-07-17_FarmI
A_arctic-fox_Finland_1840_2023_H5N1_2023-08-17_FarmG
A_otter_Finland_1387_2023_H5N1_2023-08-13
A_arctic-fox_Finland_654_2023_H5N1_2023-07-27_FarmV
A_fox_Finland_603_2023_H5N1_2023-07-20_FarmN
A_black-headed_gull_Finland_8250_2023_H5N1_2023-07-20
A_black-headed_gull_Finland_LI7827_23_23VIR6803-12_2023_H5N1_2023-07-08
A_black-headed_gull_Finland_LI7826_23_23VIR6803-11_2023_H5N1_2023-07-08
A_black-headed_gull_Finland_8134_23VIR7026-8_2023_H5N1_2023-07-21
A_black-headed_gull_Finland_8135_2023_H5N1_2023-07-193
A_arctic-fox_Finland_491_23_23VIR6803-1_2023_H5N1_2023-07-06_FarmB
A_black-headed_gull_Finland_8257_2023_H5N1_2023-07-18
A_arctic-fox_Finland_670_2023_H5N1_2023-07-31_FarmX
A_arctic-fox_Finland_668_2023_H5N1_2023-07-31_FarmX
A_arctic-fox_Finland_783_2023_H5N1_2023-08-08_FarmE
A_black-headed_gull_Finland_9418_2023_H5N1_2023-08-10
A_black-headed_gull_Finland_8244_2023_H5N1_2023-07-20
A_black-headed_gull_Finland_8599_2023_H5N1_2023-07-20
A_black-headed_gull_Finland_8598_2023_H5N1_2023-07-20
A_black-headed_gull_Finland_8145_2023_H5N1_2023-07-19
A_black-headed_gull_Finland_8243_2023_H5N1_2023-07-20
A_black-headed_gull_Finland_LI7895_23_23VIR6803-13_2023_H5N1_2023-07-11
A_black-headed_gull_Finland_LI7896_23_23VIR6803-14_2023_H5N1_2023-07-11
A_black-headed_gull_Finland_LI7897_23_23VIR6803-15_2023_H5N1_2023-07-11
A_black-headed_gull_Finland_LI7898_23_23VIR6803-16_2023_H5N1_2023-07-11
A_arctic-fox_Finland_650_2023_H5N1_2023-07-28_FarmU
A_black-headed_gull_Finland_6726_2023_H5N1_2023-06-13
A_Black-headed_gull_Netherlands_39_2023_H5N1_2023-04-17
A_Falco_peregrinus_Belgium_03518_0004_2023_H5N1_2023-04-11
A_Falco_peregrinus_Belgium_04695_0002_2023_H5N1_2023-05-07
A_Chroicocephalus_ridibundus_Belgium_03706_0003_2023_H5N1_2023-04-18
A_Vulpes_vulpes_Belgium_01784_0008_2023_H5N1_2023-02-27
A_black-headed_gull_Czech_Republic_5695-2_2023_H5N1_2023-04-13
A_black-headed_gull_Czech_Republic_5695-4_2023_H5N1_2023-04-13
A_black-headed_gull_Czech_Republic_6947-3_2023_H5N1_2023-05-03
A_Vulpes_Vulpes_Belgium_03703_0001_2023_H5N1_2023-04-14
A_Vulpes_Vulpes_Belgium_03704_0001_2023_H5N1_2023-04-14
A_Vulpes_vulpes_Belgium_02359_0006_2023_H5N1_2023-03-14
A_Vulpes_Vulpes_Belgium_04016_0001_2023_H5N1_2023-04-24
A_Vulpes_vulpes_Belgium_03117_0004_2023_H5N1_2023-03-29
A_black-headed_gull_Czech_Republic_7334-5_2023_H5N1_2023-05-11
A_turkey_Czech_Republic_7123-1_2023_H5N1_2023-05-05
A_turkey_Czech_Republic_7124-2_2023_H5N1_2023-05-05
A_turkey_Czech_Republic_7124-1_2023_H5N1_2023-05-05
A_turkey_Czech_Republic_7123-2_2023_H5N1_2023-05-05
A_black-headed_gull_Czech_Republic_5695-1_2023_H5N1_2023-04-13
A_black-headed_gull_Czech_Republic_5695-7_2023_H5N1_2023-04-13
A_peregrine_falcon_Czech_Republic_7459-1_2023_H5N1_2023-05-15
A_black-headed_gull_Czech_Republic_5487-2_2023_H5N1_2023-04-12
A_black-headed_gull_Czech_Republic_5695-3_2023_H5N1_2023-04-13
A_black-headed_gull_Austria_23025916-001_2023_H5N1_2023-02-28
A_black-headed_gull_Czech_Republic_5695-5_2023_H5N1_2023-04-13
A_black-headed_gull_Czech_Republic_5152-5_2023_H5N1_2023-04-04
A_black-headed_gull_Czech_Republic_6634-2_2023_H5N1_2023-04-24
A_black-headed_gull_Czech_Republic_5425-1_2023_H5N1_2023-04-11
A_black-headed_gull_Czech_Republic_5425-3_2023_H5N1_2023-04-11
A_black-headed_gull_Czech_Republic_5452-4_2023_H5N1_2023-04-11
A_Chroicocephalus_ridibundus_Belgium_00374_0005_2023_H5N1_2023-01-12
A_black-headed_gull_Austria_23037203-001_2023_H5N1_2023-01-21
A_Falco_peregrinus_Belgium_01145_0005_2023_H5N1_2023-08-07
A_Falco_peregrinus_Belgium_01145_0004_2023_H5N1_2023-02-07
A_Chroicocephalus_ridibundus_Belgium_01145_0008_2023_H5N1_2023-02-07
A_Chroicocephalus_ridibundus_Belgium_03706_0002_2023_H5N1_2023-04-18
A_Vulpes_vulpes_Belgium_01924_0003_2023_H5N1_2023-03-02
A_Pavo_cristatus_Belgium_02559_0001_2023_H5N1_2023-03-17
A_Vulpes_vulpes_Belgium_01295_0002_2023_H5N1_2023-02-10
A_black-headed_gull_Austria_23030829-001_2023_H5N1_2023-03-08
A_Falco_peregrinus_Belgium_02423_0004_2023_H5N1_2023-03-15
A_black-headed_gull_Czech_Republic_5295-3_2023_H5N1_2023-04-03
A_Gallus_gallus_Belgium_14828_0003_2022_H5N1_2022-12-27
A_Meleagris_gallopavo_Belgium_14826_0002_2022_H5N1_2022-12-24
A_Falco_peregrinus_Belgium_01823_0003_2023_H5N1_2023-03-01
A_fox_France_23P002844_2023_H5N1_2023-02-10
A_Vulpes_vulpes_Belgium_02359_0002_2023_H5N1_2023-03-14
A_Black-headed_Gull_Netherlands_77_2023_H5N1_2023-02-11
A_Chroicocephalus_ridibundus_Belgium_01145_0006_2023_H5N1_2023-02-07
A_domestic_duck_England_049343_2023_H5N1_2023-03-30
A_guinea_fowl_England_049422_2023_H5N1_2023-03-30
A_black-headed_gull_Austria_23029679_2023_H5N1_2023-03-07
A_Vulpes_Vulpes_Belgium_03703_0007_2023_H5N1_2023-04-14
A_black-headed_gull_Czech_Republic_6577-3_2023_H5N1_2023-04-24
A_peregrine_falcon_Czech_Republic_7735-2_2023_H5N1_2023-05-17
A_Black-Headed_Gull_England_350132_2023_H5N1_2023-05-09
A_black-headed_gull_Austria_23080170-008_2023_H5N1_2023-05-05
A_black-headed_gull_Czech_Republic_5487-4_2023_H5N1_2023-04-12
A_black-headead_gull_Spain_416-11_23VIR1687-6_2023_H5N1_2023-01-16
A_black-headed_gull_Czech_Republic_5452-1_2023_H5N1_2023-04-11
A_black-headead_gull_Spain_824-9_23VIR1687-24_2023_H5N1_2023-02-09
A_black-headed_gull_Austria_23031105-002_2023_H5N1_2023-03-08
A_black-headed_gull_Austria_23033899-002_2023_H5N1_2023-03-14
A_black-headed_gull_Czech_Republic_6577-5_2023_H5N1_2023-04-24
A_Common_tern_Luxembourg_23109715_2023_H5N1_2023-05-10
A_black-headed_gull_Luxembourg_23111454_2023_H5N1_2023-05-12
A_Black-headed_gull_Netherlands_40_2023_H5N1_2023-04-19
A_Gallus_gallus_Belgium_01742_0001_2023_H5N1_2023-02-27
A_black-headed_gull_Czech_Republic_5152-4_2023_H5N1_2023-04-04
A_black-headed_gull_Czech_Republic_6947-4_2023_H5N1_2023-05-03
A_Black-headed_gull_Netherlands_17_2023_H5N1_2023-03-05
A_black-headed_gull_Spain_171-1_23VIR1687-20_2023_H5N1_2023-01-09
A_black-headed_gull_Austria_23056483-003_2023_H5N1_2023-04-27
A_fox_Italy_23VIR3885-1_2023_H5N1_2023-04-07
A_black-headed_gull_Czech_Republic_7334-4_2023_H5N1_2023-05-11
A_black-headed_gull_Italy_23VIR2383-2_2023_H5N1_2023-03-13
A_black-headed_gull_Austria_23056483-005_2023_H5N1_2023-04-27
A_black-headed_gull_Czech_Republic_6577-2_2023_H5N1_2023-04-24
A_black-headed_gull_Czech_Republic_6947-1_2023_H5N1_2023-05-03
A_black-headed_gull_Austria_23052993-001_2023_H5N1_2023-04-20
A_Vulpes_vulpes_Belgium_03117_0001_2023_H5N1_2023-03-29
A_black-headead_gull_Italy_23VIR1658-4_2023_H5N1_2023-02-20
A_black-headed_gull_Italy_23VIR1249-2_2023_H5N1_2023-02-14
A_black-headed_gull_Italy_23VIR1483-4_2023_H5N1_2023-02-16
A_black-headed_gull_Czech_Republic_5487-5_2023_H5N1_2023-04-12
A_black-headed_gull_Italy_23VIR1484-4_2023_H5N1_2023-02-18
A_black-headed_gull_Italy_23VIR1430-8_2023_H5N1_2023-02-17
A_black-headed_gull_Italy_23VIR1476-3_2023_H5N1_2023-02-21
A_black-headed_gull_Italy_23VIR2153-1_2023_H5N1_2023-03-09
A_black-headed_gull_Italy_23VIR1203-4_2023_H5N1_2023-02-10
A_black-headed_gull_Italy_23VIR1481-4_2023_H5N1_2023-02-18
A_black-headed_gull_Italy_23VIR1515-2_2023_H5N1_2023-02-22
A_black-headed_gull_Italy_23VIR1104-4_2023_H5N1_2023-01-24
A_black-headed_gull_Italy_23VIR1203-5_2023_H5N1_2023-02-10
A_black-headed_gull_Italy_23VIR1516-2_2023_H5N1_2023-02-22
A_goshawk_Austria_23048718-001_2023_H5N1_2023-04-12
A_black-headead_gull_Italy_23VIR1667-1_2023_H5N1_2023-02-28
A_Black-headed_gull_Netherlands_37_2023_H5N1_2023-04-17
A_Black-headed_gull_Netherlands_38_2023_H5N1_2023-04-17
A_black-headed_gull_Finland_7477_2023_H5N1_2023-06-25
A_common_tern_Finland_7479_2023_H5N1_2023-06-25
A_Common_Tern_Netherlands_8_2023_H5N1_2023-06-03
A_black-headed_gull_Finland_LI7728_23_23VIR6803-9_2023_H5N1_2023-07-03
A_canada_goose_Finland_8109_23VIR7026-20_2023_H5N1_2023-07-18
A_great_crested_grebe_Finland_8111_23VIR7026-21_2023_H5N1_2023-07-18
A_raxorbill_Finland_9072_2023_H5N1_2023-07-27_Geta
A_mink_Finland_3077_2023_H5N1_2023-09-20_FarmAA
A_mink_Finland_3081_2023_H5N1_2023-09-20_FarmAA
A_mink_Finland_3079_2023_H5N1_2023-09-20_FarmAA
A_mink_Finland_3082_2023_H5N1_2023-09-20_FarmAA
A_mink_Finland_3078_2023_H5N1_2023-09-20_FarmAA
A_mink_Finland_3076_2023_H5N1_2023-09-20_FarmAA
A_mink_Finland_3080_2023_H5N1_2023-09-20_FarmAA
A_herring_gull_Finland_10812_2023_H5N1_2023-09-18
A_black-headed_gull_Finland_9079_2023_H5N1_2023-07-28
A_Black-headed_Gull_Netherlands_103_2023_H5N1_2023-06-26
A_Arctic_Tern_England_402279_2023_H5N1_2023-07-13
A_Common_Buzzard_England_125155_2023_H5N1_2023-04-17
A_Black-Headed_Gull_England_125079_2023_H5N1_2023-04-13
A_Black-Headed_Gull_England_400219_2023_H5N1_2023-04-21
A_Peregrine_falcon_Netherlands_6_2023_H5N1_2023-02-07
A_European_herring_gull_Netherlands_8_2023_H5N1_2023-03-03
A_European_herring_gull_Netherlands_11_2023_H5N1_2023-03-27
A_herring_gull_Finland_10450_2023_H5N1_2023-08-29
A_black-legged_kittiwake_Finland_8772_2023_H5N1_2023-07-23
A_black-legged_kittiwake_Finland_8808_2023_H5N1_2023-07-28
A_red_fox_Norway_2023_80_49_2_2023_H5N1_2023-06-27
A_white-tailed_eagle_Finland_10644_2023_H5N1_2023-09-02
A_white-tailed_eagle_Finland_LI7800_23_23VIR6803-10_2023_H5N1_2023-06-28
A_white-tailed_eagle_Finland_8154_23VIR7026-22_2023_H5N1_2023-07-18
A_black-headed_gull_Finland_8107_23VIR7026-7_2023_H5N1_2023-07-20
A_mute_swan_Denmark_06134-1.02_2023-07-27_H5N1_2023-07-27
A_mute_swan_Denmark_06339-1.01_2023-08-05_H5N1_2023-08-05
A_Mute_Swan_Scotland_015644_2023_H5N1_2023-01-23
A_Chicken_England_155942_2022_H5N1_2022-11-20
A_chicken_Wales_059861_2023_H5N1_2023-04-22
A_chicken_Wales_057026_2023_H5N1_2023-04-12
A_pheasant_Wales_062149_2023_H5N1_2023-04-26
A_chicken_Wales_023780_2023_H5N1_2023-02-11
H5N1 Finland 2021
A_white-tailed_eagle_Finland_9257_21VIR7689-11_2021_H5N8_2021-07-01
A_whooper_swan_Finland_9906_21VIR7689-14_2021_H5N8_2021-08-01
A_mute_swan_Finland_1325_21VIR7688-2_2021_H5N8_2021-01-01
A_pheasant_Finland_1589_21VIR7689-3_2021_H5N8_2021-02-01
A_pheasant_Finland_499_21VIR7689-1_2021_H5N8_2021-01-01
0.007

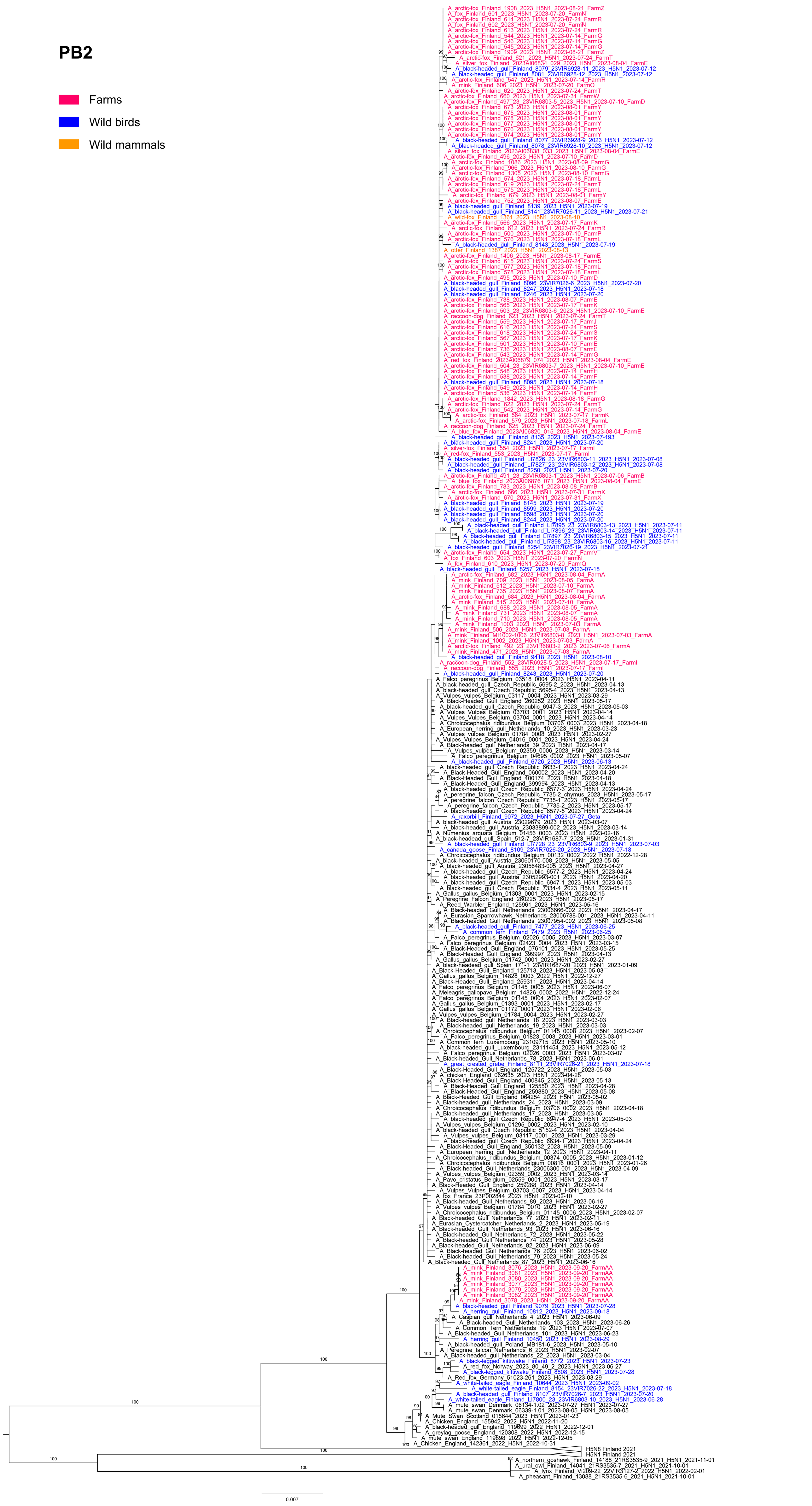
PB2
Farms
Wild birds
Wild mammals
A_arctic-fox_Finland_1908_2023_H5N1_2023-08-21_FarmZ
A_fox_Finland_601_2023_H5N1_2023-07-20_FarmN
A_arctic-fox_Finland_614_2023_H5N1_2023-07-24_FarmR
A_fox_Finland_602_2023_H5N1_2023-07-20_FarmN
A_arctic-fox_Finland_613_2023_H5N1_2023-07-24_FarmR
A_arctic-fox_Finland_544_2023_H5N1_2023-07-14_FarmG
A_arctic-fox_Finland_546_2023_H5N1_2023-07-14_FarmG
A_arctic-fox_Finland_545_2023_H5N1_2023-07-14_FarmG
A_arctic-fox_Finland_1909_2023_H5N1_2023-08-21_FarmZ
A_arctic-fox_Finland_621_2023_H5N1_2023-07-24_FarmT
A_silver_fox_Finland_2023AI06834_029_2023_H5N1_2023-08-04_FarmE
A_black-headed_gull_Finland_8079_23VIR6928-11_2023_H5N1_2023-07-12
A_black-headed_gull_Finland_8081_23VIR6928-12_2023_H5N1_2023-07-12
A_arctic-fox_Finland_547_2023_H5N1_2023-07-14_FarmH
A_mink_Finland_606_2023_H5N1_2023-07-20_FarmO
A_arctic-fox_Finland_620_2023_H5N1_2023-07-24_FarmT
A_arctic-fox_Finland_660_2023_H5N1_2023-07-31_FarmW
A_arctic-fox_Finland_497_23_23VIR6803-5_2023_H5N1_2023-07-10_FarmD
A_arctic-fox_Finland_673_2023_H5N1_2023-08-01_FarmY
A_arctic-fox_Finland_675_2023_H5N1_2023-08-01_FarmY
A_arctic-fox_Finland_678_2023_H5N1_2023-08-01_FarmY
A_arctic-fox_Finland_677_2023_H5N1_2023-08-01_FarmY
A_arctic-fox_Finland_676_2023_H5N1_2023-08-01_FarmY
A_arctic-fox_Finland_674_2023_H5N1_2023-08-01_FarmY
A_black-headed_gull_Finland_8077_23VIR6928-9_2023_H5N1_2023-07-12
A_black-headed_gull_Finland_8078_23VIR6928-10_2023_H5N1_2023-07-12
A_silver_fox_Finland_2023AI06838_033_2023_H5N1_2023-08-04_FarmE
A_arctic-fox_Finland_496_2023_H5N1_2023-07-10_FarmD
A_arctic-fox_Finland_1086_2023_H5N1_2023-08-09_FarmG
A_arctic-fox_Finland_966_2023_H5N1_2023-08-10_FarmG
A_arctic-fox_Finland_1305_2023_H5N1_2023-08-10_FarmG
A_arctic-fox_Finland_574_2023_H5N1_2023-07-18_FarmL
A_arctic-fox_Finland_619_2023_H5N1_2023-07-24_FarmT
A_arctic-fox_Finland_575_2023_H5N1_2023-07-18_FarmL
A_arctic-fox_Finland_679_2023_H5N1_2023-08-01_FarmY
A_arctic-fox_Finland_752_2023_H5N1_2023-08-07_FarmE
A_black-headed_gull_Finland_8139_2023_H5N1_2023-07-19
A_black-headed_gull_Finland_8141_23VIR7026-11_2023_H5N1_2023-07-21
A_wild-fox_Finland_1361_2023_H5N1_2023-08-10
A_arctic-fox_Finland_566_2023_H5N1_2023-07-17_FarmK
A_arctic-fox_Finland_612_2023_H5N1_2023-07-24_FarmR
A_arctic-fox_Finland_500_2023_H5N1_2023-07-10_FarmP
A_arctic-fox_Finland_576_2023_H5N1_2023-07-18_FarmL
A_black-headed_gull_Finland_8143_2023_H5N1_2023-07-19
A_otter_Finland_1387_2023_H5N1_2023-08-13
A_arctic-fox_Finland_1406_2023_H5N1_2023-08-17_FarmE
A_arctic-fox_Finland_615_2023_H5N1_2023-07-24_FarmS
A_arctic-fox_Finland_577_2023_H5N1_2023-07-18_FarmL
A_arctic-fox_Finland_578_2023_H5N1_2023-07-18_FarmL
A_arctic-fox_Finland_495_2023_H5N1_2023-07-10_FarmD
A_black-headed_gull_Finland_8096_23VIR7026-6_2023_H5N1_2023-07-20
A_black-headed_gull_Finland_8247_2023_H5N1_2023-07-18
A_black-headed_gull_Finland_8246_2023_H5N1_2023-07-20
A_arctic-fox_Finland_738_2023_H5N1_2023-08-07_FarmE
A_arctic-fox_Finland_565_2023_H5N1_2023-07-17_FarmK
A_arctic-fox_Finland_503_23_23VIR6803-8_2023_H5N1_2023-07-10_FarmE
A_raccoon-dog_Finland_623_2023_H5N1_2023-07-24_FarmT
A_arctic-fox_Finland_559_2023_H5N1_2023-07-17_FarmJ
A_arctic-fox_Finland_616_2023_H5N1_2023-07-24_FarmS
A_arctic-fox_Finland_618_2023_H5N1_2023-07-24_FarmS
A_arctic-fox_Finland_567_2023_H5N1_2023-07-17_FarmK
A_arctic-fox_Finland_501_2023_H5N1_2023-07-10_FarmE
A_arctic-fox_Finland_736_2023_H5N1_2023-08-07_FarmE
A_arctic-fox_Finland_543_2023_H5N1_2023-07-14_FarmG
A_red_fox_Finland_2023AI06879_074_2023_H5N1_2023-08-04_FarmE
A_arctic-fox_Finland_504_23_23VIR6803-7_2023_H5N1_2023-07-10_FarmE
A_arctic-fox_Finland_548_2023_H5N1_2023-07-14_FarmH
A_arctic-fox_Finland_536_2023_H5N1_2023-07-14_FarmF
A_black-headed_gull_Finland_8095_2023_H5N1_2023-07-18
A_arctic-fox_Finland_549_2023_H5N1_2023-07-14_FarmH
A_arctic-fox_Finland_536_2023_H5N1_2023-07-14_FarmF
A_arctic-fox_Finland_1842_2023_H5N1_2023-08-18_FarmG
A_arctic-fox_Finland_622_2023_H5N1_2023-07-24_FarmT
A_arctic-fox_Finland_542_2023_H5N1_2023-07-14_FarmG
A_arctic-fox_Finland_564_2023_H5N1_2023-07-17_FarmK
A_arctic-fox_Finland_579_2023_H5N1_2023-07-18_FarmL
A_raccoon-dog_Finland_625_2023_H5N1_2023-07-24_FarmT
A_blue_fox_Finland_2023AI06820_015_2023_H5N1_2023-08-04_FarmE
A_black-headed_gull_Finland_8135_2023_H5N1_2023-07-193
A_black-headed_gull_Finland_8241_2023_H5N1_2023-07-20
A_silver-fox_Finland_554_2023_H5N1_2023-07-17_FarmI
A_red-fox_Finland_553_2023_H5N1_2023-07-17_FarmI
A_black-headed_gull_Finland_LI7826_23_23VIR6803-11_2023_H5N1_2023-07-08
A_black-headed_gull_Finland_LI7827_23_23VIR6803-12_2023_H5N1_2023-07-08
A_black-headed_gull_Finland_8250_2023_H5N1_2023-07-20
A_arctic-fox_Finland_491_23_23VIR6803-1_2023_H5N1_2023-07-06_FarmB
A_blue_fox_Finland_2023AI06876_071_2023_H5N1_2023-08-04_FarmE
A_arctic-fox_Finland_783_2023_H5N1_2023-08-08_FarmB
A_arctic-fox_Finland_666_2023_H5N1_2023-07-31_FarmX
A_arctic-fox_Finland_670_2023_H5N1_2023-07-31_FarmX
A_black-headed_gull_Finland_8145_2023_H5N1_2023-07-19
A_black-headed_gull_Finland_8599_2023_H5N1_2023-07-20
A_black-headed_gull_Finland_8598_2023_H5N1_2023-07-20
A_black-headed_gull_Finland_8244_2023_H5N1_2023-07-20
A_black-headed_gull_Finland_LI7895_23_23VIR6803-13_2023_H5N1_2023-07-11
A_black-headed_gull_Finland_LI7896_23_23VIR6803-14_2023_H5N1_2023-07-11
A_black-headed_gull_Finland_LI7897_23_23VIR6803-15_2023_H5N1_2023-07-11
A_black-headed_gull_Finland_LI7898_23_23VIR6803-16_2023_H5N1_2023-07-11
A_black-headed_gull_Finland_8254_23VIR7026-19_2023_H5N1_2023-07-21
A_arctic-fox_Finland_654_2023_H5N1_2023-07-27_FarmV
A_fox_Finland_603_2023_H5N1_2023-07-20_FarmN
A_fox_Finland_610_2023_H5N1_2023-07-20_FarmQ
A_black-headed_gull_Finland_8257_2023_H5N1_2023-07-18
A_arctic-fox_Finland_682_2023_H5N1_2023-08-04_FarmA
A_mink_Finland_709_2023_H5N1_2023-08-05_FarmA
A_mink_Finland_512_2023_H5N1_2023-07-10_FarmA
A_mink_Finland_735_2023_H5N1_2023-08-07_FarmA
A_arctic-fox_Finland_894_2023_H5N1_2023-08-04_FarmA
A_mink_Finland_515_2023_H5N1_2023-07-10_FarmA
A_mink_Finland_688_2023_H5N1_2023-08-05_FarmA
A_mink_Finland_731_2023_H5N1_2023-08-07_FarmA
A_mink_Finland_710_2023_H5N1_2023-08-05_FarmA
A_mink_Finland_1003_2023_H5N1_2023-07-03_FarmA
A_mink_Finland_508_2023_H5N1_2023-07-03_FarmA
A_mink_Finland_MI1002-1006_23VIR6803-8_2023_H5N1_2023-07-03_FarmA
A_mink_Finland_1002_2023_H5N1_2023-07-03_FarmA
A_arctic-fox_Finland_492_23_23VIR6803-2_2023_2023-07-06_FarmA
A_mink_Finland_471_2023_H5N1_2023-07-03_FarmA
A_black-headed_gull_Finland_9418_2023_H5N1_2023-08-10
A_raccoon-dog_Finland_552_23VIR6928-5_2023_H5N1_2023-07-17_FarmI
A_raccoon-dog_Finland_555_2023_H5N1_2023-07-17_FarmI
A_black-headed_gull_Finland_8243_2023_H5N1_2023-07-20
A_Falco_peregrinus_Belgium_03518_0004_2023_H5N1_2023-04-11
A_black-headed_gull_Czech_Republic_5695-2_2023_H5N1_2023-04-13
A_black-headed_gull_Czech_Republic_5695-4_2023_H5N1_2023-04-13
A_Vulpes_vulpes_Belgium_03117_0004_2023_H5N1_2023-03-29
A_Black-headed_Gull_England_260252_2023_H5N1_2023-05-17
A_black-headed_gull_Czech_Republic_6947-3_2023_H5N1_2023-05-03
A_Vulpes_Vulpes_Belgium_03703_0001_2023_H5N1_2023-04-14
A_Vulpes_Vulpes_Belgium_03704_0001_2023_H5N1_2023-04-14
A_Chroicocephalus_ridibundus_Belgium_03706_0003_2023_H5N1_2023-04-18
A_European_herring_gull_Netherlands_10_2023_H5N1_2023-03-23
A_Vulpes_vulpes_Belgium_01784_0008_2023_H5N1_2023-02-27
A_Vulpes_Vulpes_Belgium_04016_0001_2023_H5N1_2023-04-24
A_Black-headed_gull_Netherlands_39_2023_H5N1_2023-04-17
A_Vulpes_vulpes_Belgium_02359_0006_2023_H5N1_2023-03-14
A_Falco_peregrinus_Belgium_04695_0002_2023_H5N1_2023-05-07
A_black-headed_gull_Finland_6726_2023_H5N1_2023-08-13
A_Black-Headed_Gull_England_060002_2023_H5N1_2023-04-20
A_Black-Headed_Gull_England_400174_2023_H5N1_2023-04-18
A_Black-Headed_Gull_England_399994_2023_H5N1_2023-04-13
A_black-headed_gull_Czech_Republic_6577-3_2023_H5N1_2023-04-24
A_peregrine_falcon_Czech_Republic_7735-2_chymus_2023_H5N1_2023-05-17
A_peregrine_falcon_Czech_Republic_7735-1_2023_H5N1_2023-05-17
A_peregrine_falcon_Czech_Republic_7735-2_2023_H5N1_2023-05-17
A_black-headed_gull_Czech_Republic_6577-5_2023_H5N1_2023-04-24
A_raxorbill_Finland_9072_2023_H5N1_2023-07-27_Geta
A_black-headed_gull_Austria_23029679_2023_H5N1_2023-03-07
A_black-headed_gull_Austria_23033899-002_2023_H5N1_2023-03-14
A_Numenius_arquata_Belgium_01456_0003_2023_H5N1_2023-02-16
A_black-headed_gull_Spain_512-7_23VIR1687-7_2023_H5N1_2023-01-31
A_black-headed_gull_Finland_LI7728_23_23VIR6803-9_2023_H5N1_2023-07-03
A_canada_goose_Finland_8109_23VIR7026-20_2023_H5N1_2023-07-18
A_Chroicocephalus_ridibundus_Belgium_00132_0002_2022_H5N1_2022-12-28
A_black-headed_gull_Austria_23060170-008_2023_H5N1_2023-05-05
A_black-headed_gull_Austria_23056483-005_2023_H5N1_2023-04-27
A_black-headed_gull_Czech_Republic_6577-2_2023_H5N1_2023-04-24
A_black-headed_gull_Austria_23052993-001_2023_H5N1_2023-04-20
A_black-headed_gull_Czech_Republic_6947-1_2023_H5N1_2023-05-03
A_black-headed_gull_Czech_Republic_7334-4_2023_H5N1_2023-05-11
A_Gallus_gallus_Belgium_01303_0001_2023_H5N1_2023-02-15
A_Peregrine_Falcon_England_260225_2023_H5N1_2023-05-17
A_Reed_Warbler_England_125961_2023_H5N1_2023-05-16
A_Black-headed_Gull_Netherlands_23006666-002_2023_H5N1_2023-04-17
A_Eurasian_Sparrowhawk_Netherlands_23006798-001_2023_H5N1_2023-04-11
A_Black-headed_Gull_Netherlands_23007954-002_2023_H5N1_2023-05-08
A_black-headed_gull_Finland_7477_2023_H5N1_2023-06-25
A_common_tern_Finland_7479_2023_H5N1_2023-06-25
A_Falco_peregrinus_Belgium_02026_0005_2023_H5N1_2023-03-07
A_Falco_peregrinus_Belgium_02423_0004_2023_H5N1_2023-03-15
A_Black-Headed_Gull_England_076101_2023_H5N1_2023-05-25
A_Black-Headed_Gull_England_399997_2023_H5N1_2023-04-13
A_Gallus_gallus_Belgium_01742_0001_2023_H5N1_2023-02-27
A_black-headed_gull_Spain_177-1_23VIR1687-20_2023_H5N1_2023-01-09
A_Black-Headed_Gull_England_125713_2023_H5N1_2023-05-03
A_Gallus_gallus_Belgium_14828_0003_2022_H5N1_2022-12-27
A_Black-Headed_Gull_England_259311_2023_H5N1_2023-04-14
A_Falco_peregrinus_Belgium_01145_0005_2023_H5N1_2023-06-07
A_Meleagris_gallopavo_Belgium_14826_0002_2022_H5N1_2022-12-24
A_Falco_peregrinus_Belgium_01145_0004_2023_H5N1_2023-02-07
A_Gallus_gallus_Belgium_01393_0001_2023_H5N1_2023-02-17
A_Gallus_gallus_Belgium_01172_0001_2023_H5N1_2023-02-06
A_Vulpes_vulpes_Belgium_01784_0004_2023_H5N1_2023-02-27
A_Black-headed_gull_Netherlands_18_2023_H5N1_2023-03-03
A_Black-headed_gull_Netherlands_19_2023_H5N1_2023-03-03
A_Chroicocephalus_ridibundus_Belgium_01145_0008_2023_H5N1_2023-02-07
A_Falco_peregrinus_Belgium_01823_0003_2023_H5N1_2023-03-01
A_Common_tern_Luxembourg_23109715_2023_H5N1_2023-05-10
A_Black-headed_gull_Luxembourg_23111454_2023_H5N1_2023-05-12
A_Falco_peregrinus_Belgium_02026_0003_2023_H5N1_2023-03-07
A_Black-headed_Gull_Netherlands_78_2023_H5N1_2023-06-01
A_great_crested_grebe_Finland_8111_23VIR7026-21_2023_H5N1_2023-07-18
A_Black-Headed_Gull_England_125722_2023_H5N1_2023-05-03
A_chicken_England_082635_2023_H5N1_2023-04-28
A_Black-Headed_Gull_England_400845_2023_H5N1_2023-05-13
A_Black-Headed_Gull_England_125550_2023_H5N1_2023-04-28
A_Black-Headed_Gull_England_259880_2023_H5N1_2023-05-08
A_Black-Headed_Gull_England_064254_2023_H5N1_2023-05-02
A_Black-headed_gull_Netherlands_24_2023_H5N1_2023-03-09
A_Chroicocephalus_ridibundus_Belgium_03706_0002_2023_H5N1_2023-04-18
A_Black-headed_gull_Netherlands_17_2023_H5N1_2023-03-05
A_black-headed_gull_Czech_Republic_6947-4_2023_H5N1_2023-05-03
A_Vulpes_vulpes_Belgium_01295_0002_2023_H5N1_2023-02-10
A_black-headed_gull_Czech_Republic_5152-4_2023_H5N1_2023-04-04
A_Vulpes_vulpes_Belgium_03117_0001_2023_H5N1_2023-03-29
A_black-headed_gull_Czech_Republic_6634-1_2023_H5N1_2023-04-24
A_Black-Headed_Gull_England_350132_2023_H5N1_2023-05-09
A_European_herring_gull_Netherlands_12_2023_H5N1_2023-04-11
A_Chroicocephalus_ridibundus_Belgium_00374_0005_2023_H5N1_2023-01-12
A_Chroicocephalus_ridibundus_Belgium_00816_0001_2023_H5N1_2023-01-26
A_Black-headed_Gull_Netherlands_23006300-001_2023_H5N1_2023-04-09
A_Vulpes_vulpes_Belgium_02359_0002_2023_H5N1_2023-03-14
A_Pavo_cristatus_Belgium_02559_0001_2023_H5N1_2023-03-17
A_Black-Headed_Gull_England_260268_2023_H5N1_2023-04-14
A_Vulpes_Vulpes_Belgium_03703_0007_2023_H5N1_2023-04-14
A_fox_France_23P002844_2023_H5N1_2023-02-10
A_Black-headed_Gull_Netherlands_89_2023_H5N1_2023-06-16
A_Vulpes_vulpes_Belgium_01784_0010_2023_H5N1_2023-02-27
A_Chroicocephalus_ridibundus_Belgium_01145_0006_2023_H5N1_2023-02-07
A_Black-headed_Gull_Netherlands_77_2023_H5N1_2023-02-11
A_Eurasian_Oystercatcher_Netherlands_2_2023_H5N1_2023-05-19
A_Black-headed_Gull_Netherlands_93_2023_H5N1_2023-06-16
A_Black-headed_Gull_Netherlands_72_2023_H5N1_2023-05-22
A_Black-headed_Gull_Netherlands_74_2023_H5N1_2023-05-28
A_Black-headed_Gull_Netherlands_82_2023_H5N1_2023-06-09
A_Black-headed_Gull_Netherlands_76_2023_H5N1_2023-06-02
A_Black-headed_Gull_Netherlands_79_2023_H5N1_2023-05-24
A_Black-headed_Gull_Netherlands_87_2023_H5N1_2023-06-16
A_mink_Finland_3076_2023_H5N1_2023-09-20_FarmAA
A_mink_Finland_3081_2023_H5N1_2023-09-20_FarmAA
A_mink_Finland_3080_2023_H5N1_2023-09-20_FarmAA
A_mink_Finland_3077_2023_H5N1_2023-09-20_FarmAA
A_mink_Finland_3079_2023_H5N1_2023-09-20_FarmAA
A_mink_Finland_3082_2023_H5N1_2023-09-20_FarmAA
A_mink_Finland_3078_2023_H5N1_2023-09-20_FarmAA
A_black-headed_gull_Finland_9079_2023_H5N1_2023-07-28
A_herring_gull_Finland_10812_2023_H5N1_2023-09-18
A_Caspian_gull_Netherlands_4_2023_H5N1_2023-06-09
A_Black-headed_Gull_Netherlands_103_2023_H5N1_2023-06-26
A_Common_Tern_Netherlands_19_2023_H5N1_2023-07-07
A_Black-headed_Gull_Netherlands_101_2023_H5N1_2023-06-23
A_herring_gull_Finland_10450_2023_H5N1_2023-08-29
A_black-headed_gull_Poland_MB181-6_2023_H5N1_2023-05-10
A_Peregrine_falcon_Netherlands_6_2023_H5N1_2023-02-07
A_Black-headed_gull_Netherlands_22_2023_H5N1_2023-03-04
A_black-legged_kittiwake_Finland_8772_2023_H5N1_2023-07-23
A_red_fox_Norway_2023_80_49_2_2023_H5N1_2023-06-27
A_black-legged_kittiwake_Finland_8808_2023_H5N1_2023-07-28
A_Red_fox_Germany_51023-261_2023_H5N1_2023-03-29
A_white-tailed_eagle_Finland_10644_2023_H5N1_2023-09-02
A_white-tailed_eagle_Finland_8154_23VIR7026-22_2023_H5N1_2023-07-18
A_black-headed_gull_Finland_8107_23VIR7026-7_2023_H5N1_2023-07-20
A_white-tailed_eagle_Finland_LI7800_23_23VIR6803-10_2023_H5N1_2023-06-28
A_mute_swan_Denmark_06134-1_02_2023-07-27_H5N1_2023-07-27
A_mute_swan_Denmark_06339-1_01_2023-08-05_H5N1_2023-08-05
A_Mute_Swan_Scotland_015644_2023_H5N1_2023-01-23
A_Chicken_England_155942_2022_H5N1_2022-11-20
A_black-headed_gull_England_119899_2022_H5N1_2022-12-01
A_greylag_goose_England_120308_2022_H5N1_2022-12-15
A_mute_swan_England_119898_2022_H5N1_2022-12-05
A_Chicken_England_142361_2022_H5N1_2022-10-31
H5N8 Finland 2021
H5N1 Finland 2021
A_northern_goshawk_Finland_14188_21RS3535-9_2021_H5N1_2021-11-01
A_ural_owl_Finland_14041_21RS3535-7_2021_H5N1_2021-10-01
A_lynx_Finland_Vi209-22_22VIR31271-2_2022_H5N1_2022-02-01
A_pheasant_Finland_13088_21RS3535-6_2021_H5N1_2021-10-01
0.007
